# *Z*-Selective iridium-catalyzed cross-coupling of allylic carbonates and α-diazo esters†
†Electronic supplementary information (ESI) available: Full procedures, computational details and characterization data. See DOI: 10.1039/c7sc04283c


**DOI:** 10.1039/c7sc04283c

**Published:** 2017-10-24

**Authors:** Bryce N. Thomas, Patrick J. Moon, Shengkang Yin, Alex Brown, Rylan J. Lundgren

**Affiliations:** a Department of Chemistry , University of Alberta , Edmonton , Alberta T6G 2G2 , Canada . Email: rylan.lundgren@ualberta.ca

## Abstract

A well-defined Ir–allyl complex catalyzes the *Z*-selective cross-coupling of allyl carbonates with α-aryl diazo esters. The process overrides the large thermodynamic preference for *E*-products typically observed in metal-mediated coupling reactions to enable the synthesis of *Z*,*E*-dieneoates in good yield with selectivities consistently approaching or greater than 90 : 10. This transformation represents the first productive merger of Ir–carbene and Ir–allyl species, which are commonly encountered intermediates in allylation and cyclopropanation/E–H insertion catalysis. Potentially reactive functional groups (aryl halides, ketones, nitriles, olefins, amines) are tolerated owing to the mildness of reaction conditions. Kinetic analysis of the reaction suggests oxidative addition of the allyl carbonate to an Ir-species is rate-determining. Mechanistic studies uncovered a pathway for catalyst activation mediated by NEt_3_.

## Abstract 2

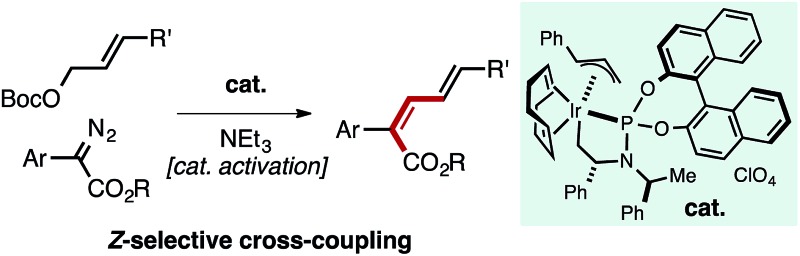
A well-defined Ir-based catalyst promotes the *Z*-selective coupling of allylic carbonates and α-diazo esters.

## Introduction

**Fig. 1:**
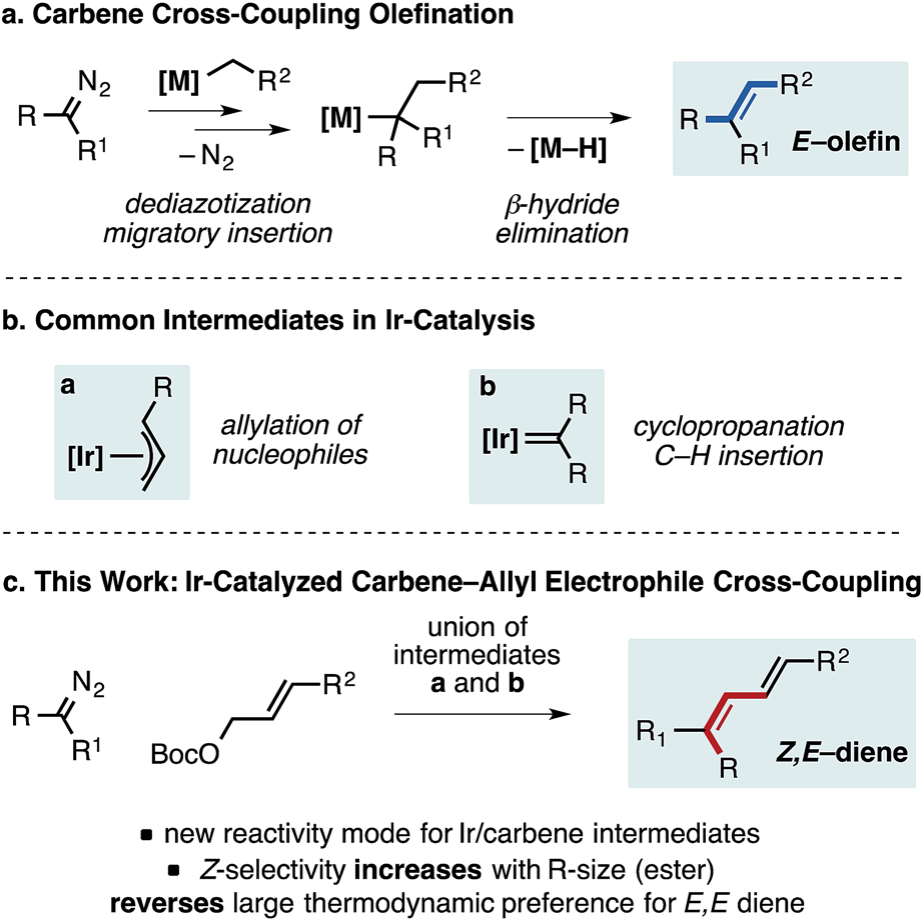
Pathway for metal-catalyzed olefination reactions of carbene precursors, common Ir-intermediates in catalysis and overview of the process reported herein to generate *Z*,*E*-dienes.

Metal-catalyzed cross-couplings involving carbene intermediates are a valuable set of transformations to generate new carbon–carbon bonds and molecular complexity from simple building blocks.^1^–^3^ A commonly exploited pathway to generate substituted olefins from carbene-precursors, such as α-diazo esters or *N*-tosylhydrazones, involves β-hydride elimination from a metal–alkyl species generated *via* a migratory insertion process. These reactions are initiated by the formation of a metal carbene intermediate, with the β-hydrogen containing substrate being either the cross-coupling partner (as depicted in Fig. 1a) or the carbene precursor.2*b* This general concept has been used in a broad series of Pd-catalyzed coupling reactions of *in situ* generated carbenes with various reaction partners to generate polysubstituted *E*-alkenes and *E*,*E*-dienes. In these processes, the stereoselectivity of the olefination is controlled by the relative energetics of species leading to *syn* β-hydride elimination, as they are in classical Heck-type processes.^4^ Wang and co-workers have demonstrated the ability of allylic halides,^5^ allenes,^6^ vinyl boronic acids,^7^ and vinyl cyclopropanes^8^ to undergo interception with various metal carbene intermediates to generate *E*-dienes.^9^ While these are powerful synthetic transformations, control over the stereochemistry of the newly formed carbon–carbon double bond that overrides the inherent preference for *E*-alkene products remains an unaddressed challenge in cross-coupling methodology involving metal carbenes.

Inspired by the rapid development of Pd-, Cu-, and Rh-catalyzed carbene cross-coupling type reactions, we questioned whether alternative transition metals could afford unique reactivity and selectivity in alkene-generating processes. Given the widespread use of both Ir–allyl and Ir–carbene intermediates in allylic substitution and carbene insertion chemistry respectively (Fig. 1b), we sought to merge these species to enable a new reactivity mode in Ir-catalysis.^10^ Herein we report that a well-defined Ir–allyl complex catalyzes the *Z*-selective olefination of allyl carbonates with α-aryl diazo esters to provide *Z*,*E*-dienoates (Fig. 1c). Increasing the steric demand of the ester substituent of the diazo compound was found to enhance the *Z*-selectivity of the reaction, overcoming the large thermodynamic preference for the formation of the *E*,*E*-diene products typically generated by conventional catalytic methods.^11^–^14^ This transformation represents a rare example of a formal migratory insertion process of an Ir–allyl intermediate^15^,^16^ and proceeds by a catalyst activation step promoted by NEt_3_ involving the generation of an allylic ammonium species. The method helps to address a methodological gap in classical olefination chemistry, as modified Horner–Wadsworth–Emmons processes fail to delivery bulky dienyl esters with appreciable selectivity.

## Results and discussion

### Reaction development

In the attempt to intercept an Ir–allyl intermediate with a carbene precursor, a range of metal catalysts and ligands were explored to promote coupling between an α-aryl diazo ester^17^ and an allylic electrophile. Table 1A provides a snapshot of the performance of various Ir- (and Rh-) complexes previously established as active allylation catalysts. Only the use of [Ir(COD)Cl]_2_ and the phosphoramidite ligand **L1** as the catalyst mixture formed an appreciable amount of product (12%). Given reports by Hartwig and Helmchen demonstrating the benefits of allyl-ligated, ligand-cyclometalated cationic Ir species featuring **L1** in allylic substitution reactions,^18^–^20^ complex **1** was prepared and used as the catalyst in the carbene coupling reaction. Use of **1** resulted in a dramatic improvement in yield of the desired product (85%) in ∼2 : 1 selectivity for the *Z*,*E*-diene isomer. Other related catalyst structures, including **L1**Ir(COD)Cl, cyclometalated Ir(i)**L1** complexes without an allyl ligand, and ligand-modified versions of the standard catalyst **1** proved considerably less reactive, as was a variant of Krische's Ir-SEGPHOS allylation catalyst.^21^*In situ* generation of **1** with [Ir(COD)Cl]_2_, **L1**, cinnamyl carbonate and AgClO_4_ did not provide significant product. Diene products arising from insertion into a branched Ir–allyl species were not observed in any case.

**Table 1:** A. Catalyst identification for the *Z*-selective cross-coupling of α-diazo esters and allylic carbonates. B. Effect of key reaction parameters and ester group on reactivity. See the ESI for additional optimization details^*a*^ ^,^^*b*^

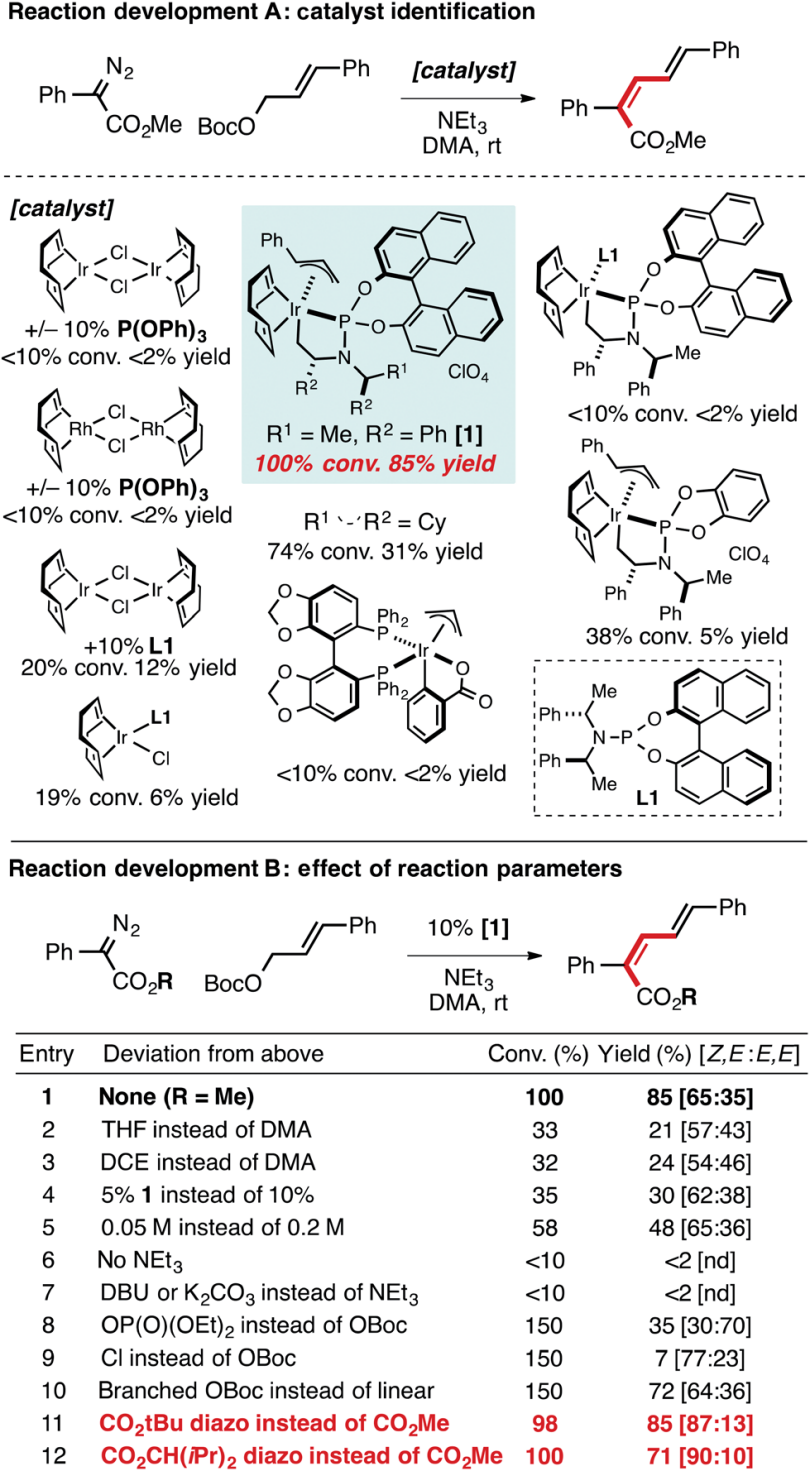

^*a*^1.5 equiv. cinnamylOBoc, 3.0 equiv. NEt_3_, 10% [catalyst], 18 h. Conv. and yields determined by calibrated ^1^H NMR, *Z*,*E*/*E*,*E* = ∼65 : 35, see ESI for full details.

^*b*^1.5 equiv. cinnamylOBoc, 3.0 equiv. NEt_3_, 18–24 h. conv. (based on 150 mol% electrophile) and yields determined by ^1^H NMR calibrated with an internal standard, see ESI for full details, bolded entries are isolated yields.

Reaction parameters important to observe high yields, including solvent, catalyst loading, and concentration are shown in Table 1B (entries 1–5). NEt_3_ is vital for product formation (entry 6); other inorganic and amine bases provided effectively no product (entry 7). Alternative leaving groups were inferior to *tert*-butyl carbonate, however a phosphonate ester substrate yielded product with a reversal in selectivity (∼2 : 1 *E*-selectivity entry 8). Branched allylic carbonates could be used as substrates, but in general provided reduced yields (entry 10). The formation of the linear carbonate over the course of the reaction was observed with branched allylic starting materials. The use of corresponding methyl carbonate substrate led to methyl ether formation instead of diene.

Increasing the steric demand of the R-group on the α-aryl diazo ester led to a significant improvement in the *Z*-selectivity of the newly generated olefin (entries 11 and 12, up to 90 : 10 selectivity). The observation of *Z*,*E*-product formation is particularly remarkable in consideration of the large thermodynamic preference for the *E*,*E* isomer over the *Z*,*E* product, with the calculated relative Δ*G* = 6.5 kJ mol^–1^ at 25 °C; *Z*,*E* : *E*,*E* ∼ 5 : 95 for product **2** where R = *t*-Bu (see ESI† for details, the thermodynamic ratio is similar for the corresponding methyl ester). These results are in direct contrast to the well-established preference for related Pd-catalyzed reactions to generate *E*-products *via* β-hydride elimination processes.^4^,^5^,^9^

In light of the scarcity of reports concerning the *Z*-selective olefination of carbonyls with α-aryl acetate-derived phosphates,^13^,^22^ the venerable Still–Gennari modification^23^ of the Horner–Wadsworth–Emmons reaction was explored in an attempt to generate similar *Z*,*E*-diene products. Olefination of cinnamaldehyde with a bulky bis(trifluoroethyl)phosphonate reagent resulted in formation of diene **2** with poor selectivity (*Z*,*E* : *E*,*E* = 42 : 58), further highlighting the difficulty associated with the stereo-controlled preparation of this class of diene (eqn (1)).^24^1
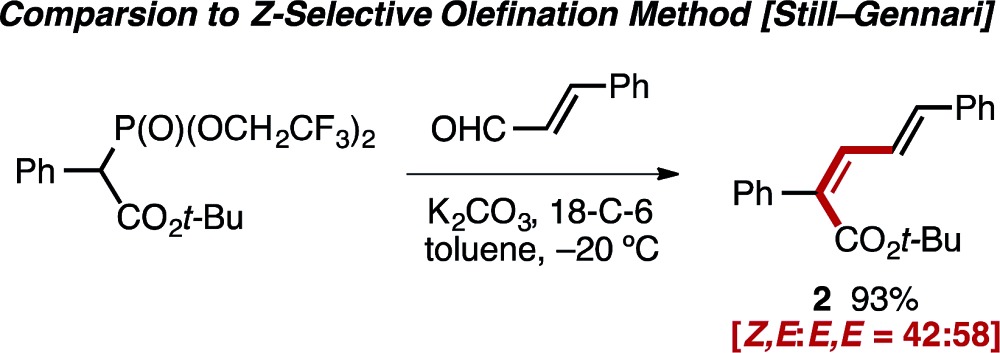



### Reaction scope and limitations

The Ir-catalyzed carbene cross-coupling reaction provides synthetically useful yields and good *Z*-selectivities across a range of aryl-substituted allylic carbonates with bulky α-diazo esters (Table 2). The reaction tolerates halogen substitution at the 2, 3, and 4-positions of cinnamyl derivatives, including potentially reactive aryl bromide groups, and proceeds with similar effectiveness for electron-rich and electron-poor aryl allylic carbonates. A dienyl electrophile substrate delivered the desired *Z*,*E*,*E* triene with excellent *Z*-selectivity (**12**, 95 : 5). Halogen groups were also tolerated on the α-aryl diazo ester partner, as were electron-withdrawing groups (keto and cyano). A notable difference in selectivity was observed when electron-withdrawing groups (keto and cyano) are placed at either the 4-position (**15**, 95 : 5 *Z*,*E* : *E*,*E*) *versus* the 3-position (**22**, 63 : 37 *Z*,*E* : *E*,*E*). Less successful substrates include alkyl-substituted allylic carbonates (**13**) and thienyl α-diazo esters (**23**, see the ESI† for additional examples).

**Table 2:** Scope of the Ir-catalyzed cross-coupling of α-aryl diazo esters and allylic carbonates^*a*^

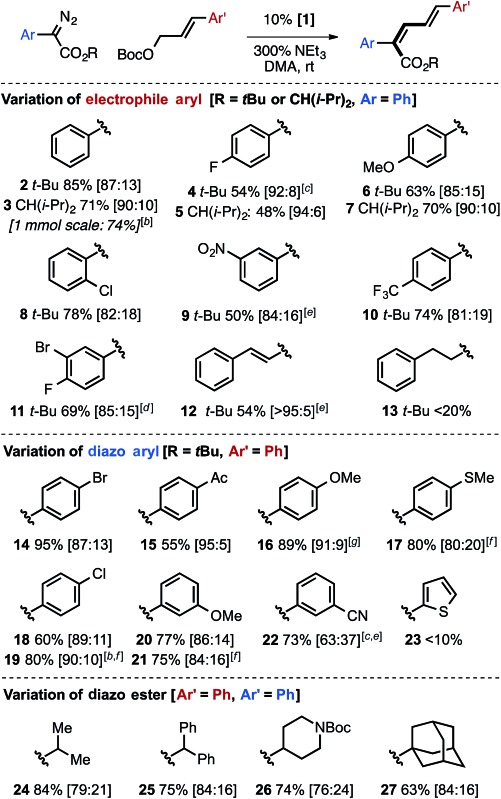

^*a*^1.5 equiv. allylic carbonate, 0.3–0.4 M 16–28 h, yields are of combined diene isomers with *Z*,*E*/*E*,*E* indicated in brackets.

^*b*^Pure *Z*,*E*-product, 7.5 mol% **1**.

^*c*^Crude ratio, isolated >95.5.

^*d*^At 35 °C.

^*e*^Determined by ^1^H NMR.

^*f*^15 mol% **1**.

^*g*^R = CH(iPr)_2_.

Variation of the ester group clearly demonstrated that an increase in the size of the alkyl group on the ester increased *Z*-selectivity, with isopropyl (**24**), diphenylmethyl (**25**), 4-*N*-Boc piperidinyl (**26**), or 1-adamantyl (**27**) groups giving better selectivity compared to methyl (Table 1B, entry 1), but less than that of the *tert*-butyl or diisopropylmethyl ester derivatives.

**Fig. 2:**
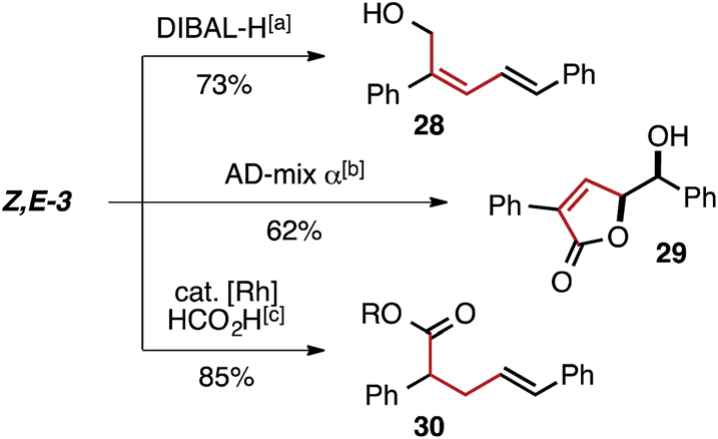
Use of *Z*,*E*-dienoate **3** as a precursor for selective transformations. (a) DIBAL-H (2.5 equiv.) CH_2_Cl_2_, rt (b) AD-mix, MeSO_2_NH_2_, *t*-BuOH/H_2_O, rt (c) 2.5 mol% [Rh(COD)Cl]_2_, 15 mol% PPh_3_, HCO_2_H/NEt_3_ (5 : 2), MeCN, 35 °C.

The selective generation of *Z*,*E*-dienyl ester products affords a useful synthetic building block, primed for further functionalization. For example, ester reduction with DIBAL-H, Sharpless' asymmetric dihydroxylation followed by lactonization, and regioselective Rh-catalyzed olefin reduction can each be used to obtain an array of new products in which the *Z*-olefin unit remains intact or directs reactivity (Fig. 2).

### Stoichiometric and kinetic studies

**Fig. 3:**
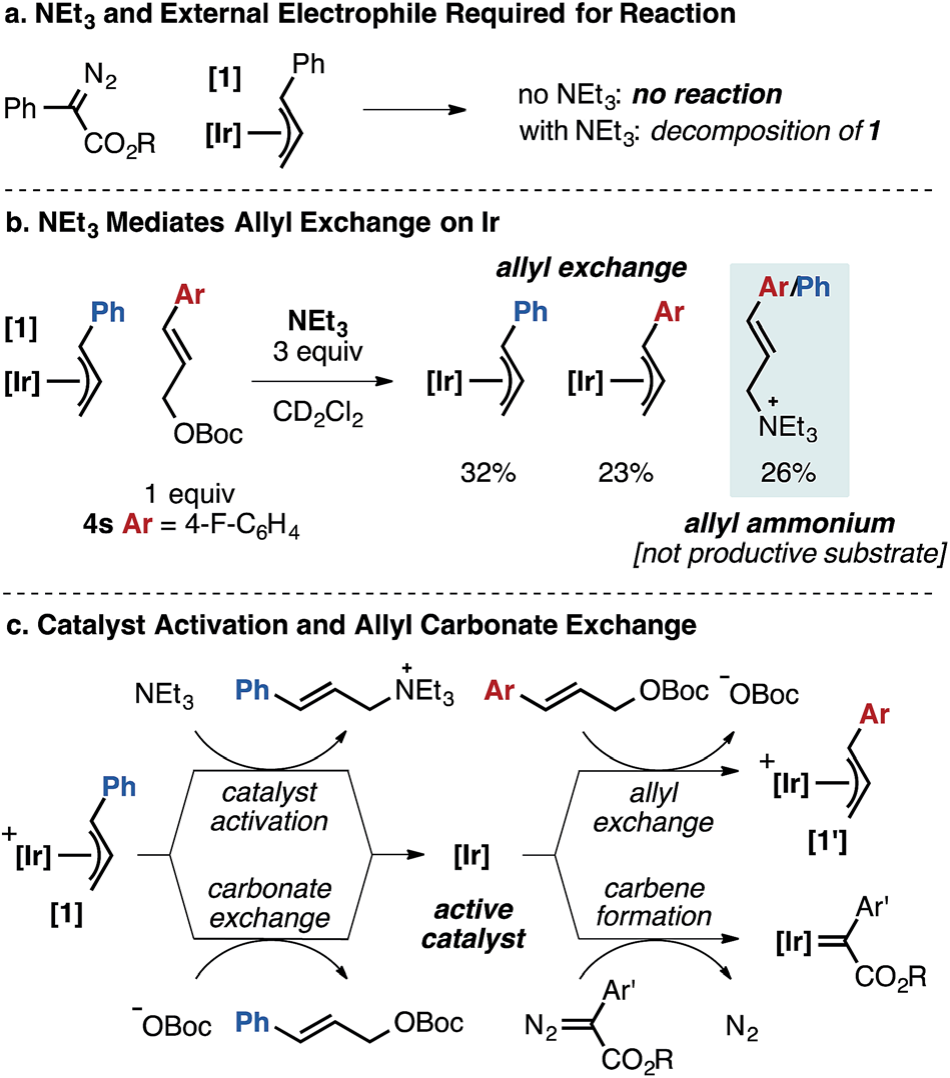
Mechanistic aspects of the Ir-catalyzed cross-coupling of α-aryl diazo esters and allylic carbonates based on stoichiometric reactions (steps drawn as irreversible for clarity).

While we do not have a complete mechanistic understanding of the Ir-catalyzed olefination process at this time, stoichiometric and kinetic experiments have provided valuable mechanistic insights. In contrast to Pd-catalyzed reactions of allyl electrophiles and α-diazo esters,^5^ direct reaction between an Ir–allyl species and the carbene precursor is not observed (Fig. 3a). When **1** is added to 1 or 10 equivalents of α-diazo ester, unreacted starting materials are recovered. The same reaction conducted in the presence of NEt_3_ leads to decomposition of **1** to a complex mixture and <5% product formation. In the absence of α-diazo ester, NEt_3_ promotes allyl exchange between complex **1** and allylic carbonate **4s**, concurrently generating allylic ammonium species (Fig. 3b).

We have observed *N*-allyl ammonium species by ^1^H NMR to form rapidly under the standard reaction conditions. Control experiments demonstrated that the allylic ammonium species is not converted into diene product under the standard conditions.

Taken together, these experiments suggest that the role of NEt_3_ is to activate Ir–allyl species **1** toward Ir–carbene formation by generating an open coordination site, likely the first mechanistic step in the product forming cycle (Fig. 3c). Notably, bulkier amine bases (DIPEA, N(*i*-Bu)_3_) fail to generate allylic ammonium species under the standard conditions and product is not observed when using these bases.^25^

**Fig. 4:**
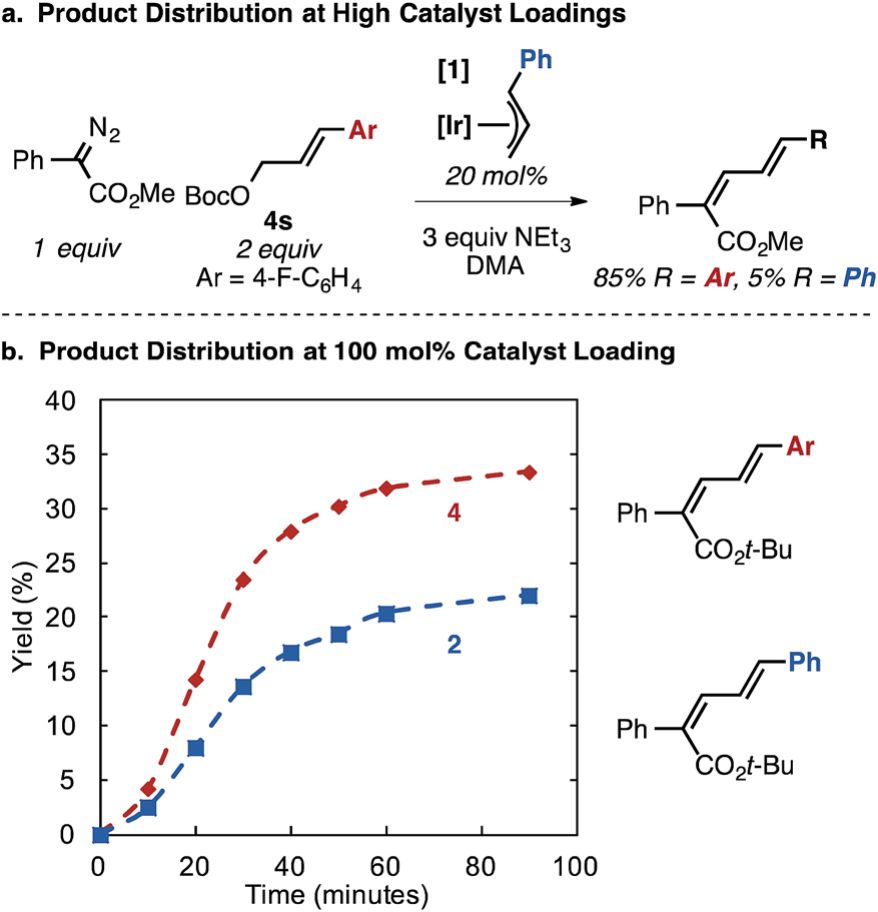
Ir–allyl/allyl carbonate cross-over experiments. At high catalyst loadings (a), small amounts of Ir–allyl crossover diene product is observed, which increases at 100 mol% catalyst (b).

In a reaction using the aryl fluoride allyl carbonate **4s** at high catalyst loading (20 mol% **1**), the catalyst cinnamyl cross-over product is observed in 5% yield along with 85% product from **4s** (Fig. 4a), supporting the series of catalyst activation steps and allylic exchange processes in Fig. 2c.^26^ Stoichiometric reactions using 1 equivalent of **1** mirror these observations, as significant crossover product is observed between the catalyst bound allyl group (20% yield) in comparison to the allyl unit originating from the carbonate substrate (35% yield). The two diene products are generated concurrently (Fig. 4b). Collectively, these results suggest allyl exchange processes at Ir occur on the time scale with product forming steps involving a putative Ir–carbene intermediate.

**Fig. 5:**
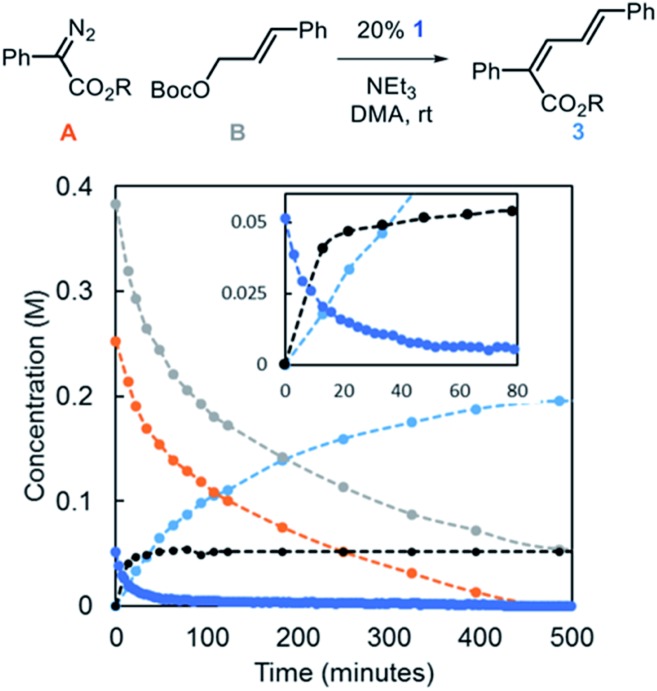
Representative kinetic profile of the Ir-catalyzed coupling of α-aryl diazo esters and allylic carbonates [R = CH(*i*-Pr)_2_].

Reaction progress kinetic analysis (RPKA) was used to gain additional insights into the nature of the mechanism.^27^ A representative RPKA plot is shown in Fig. 5. The rapid formation of allylic ammonium **D** is observed with concurrent depletion of complex **1** (inset) in line with the observations that NEt_3_ acts to activate Ir-precatalysts with bound allyl fragments. The amount of allylic ammonium **D** increases slowly after the initial burst, indicative of regeneration of Ir–allyl species in relatively low concentration. Attempts to characterize Ir-based species in solution during catalysis by ^31^P NMR were not successful.

**Fig. 6:**
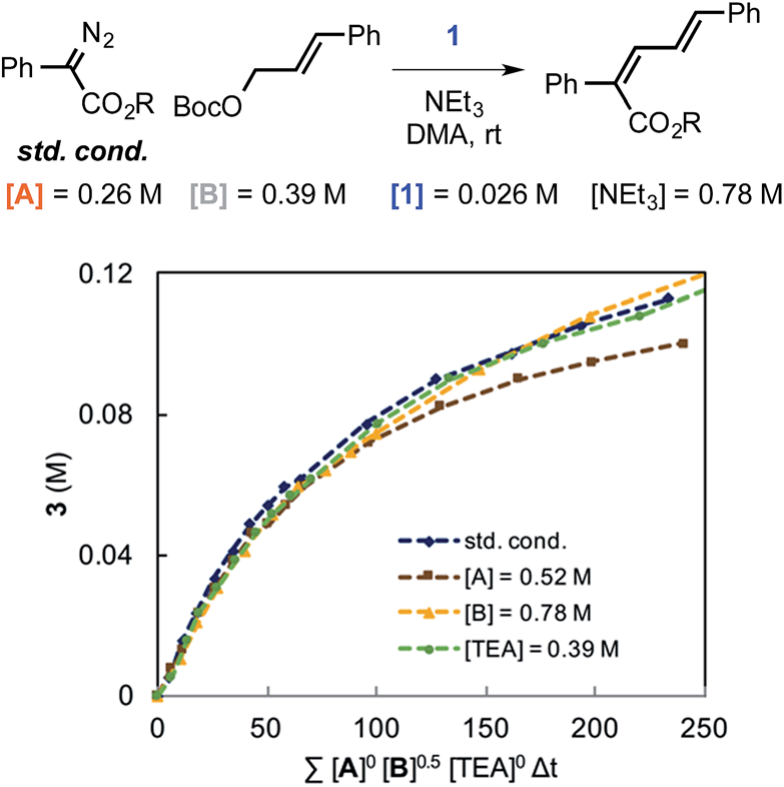
Rate law determination by variable time normalization plots for the Ir-catalyzed coupling of α-diazo esters and allylic carbonates.

Using variable time normalization plots,^28^ the rate law of the reaction was determined to be zero order in α-diazo ester and NEt_3_, while partial positive order (∼0.5) in allyl carbonate (Fig. 6, see Fig S2–S4 in ESI† for additional plots and discussion). The order in allylic carbonate could be rationalized by the electrophile being involved in a rate-determining product forming step, but also causing the regeneration of off-cycle Ir–allyl species similar to **1** (as in Fig. 3c); the slow growth of allylic ammonium salt (Fig. 5) also suggests this.

**Fig. 7:**
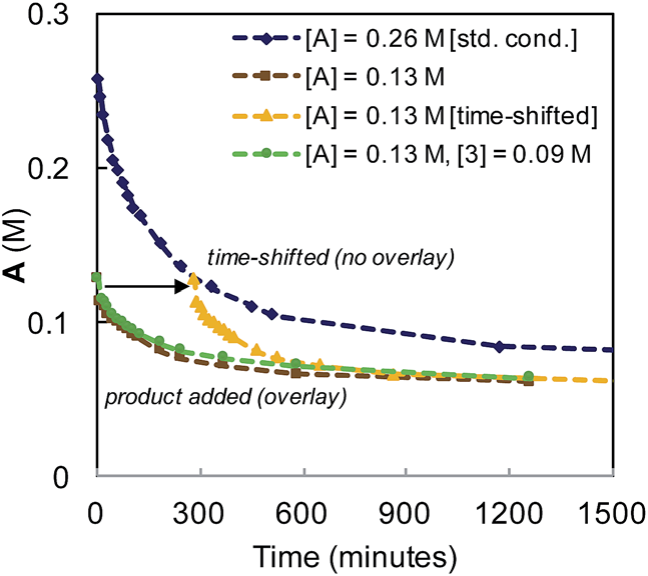
Kinetic profile for the Ir-catalyzed coupling of α-diazo esters and allylic carbonates employing varying initial concentrations of α-diazo ester (**A**), and product (**3**).

The reaction was observed to be first-order in catalyst **1** at early time points, but deviated over the course of the reaction (see Fig. S5 in the ESI†). Catalyst stability was examined by interrogating temporal concentration profiles for the process at varied initial concentrations of α-diazo ester (**A**) and diene product (**3**, Fig. 7). For reactions conducted with half the typical concentration of α-diazo ester, a lack of overlay in the time-shifted profile with the standard reaction conditions is observed (the reaction proceeds faster), indicating catalyst deactivation or substrate inhibition. Reactions conducted with an initial product concentration of 0.09 M (corresponding to ∼35% diene formation) to mimic the time-adjusted experiment resulted in direct overlay with the same experiment without added product, ruling out catalyst inhibition by diene product.

**Fig. 8:**
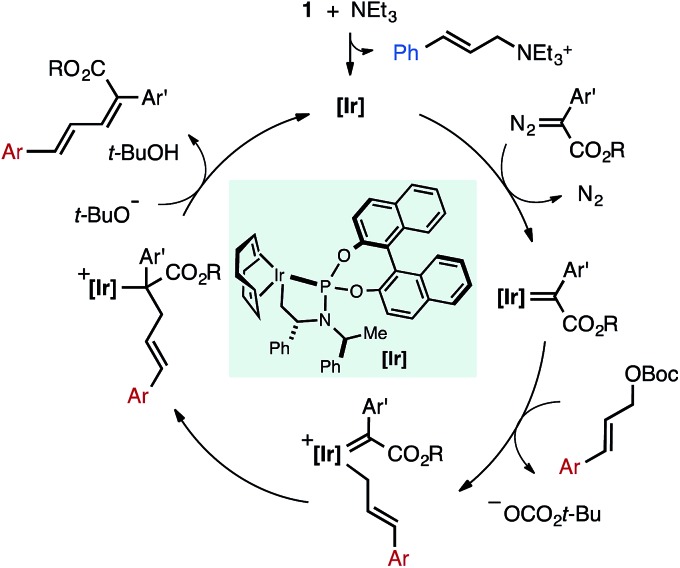
Potential mechanism for the Ir-catalyzed cross-coupling of α-aryl diazo esters and allylic carbonates.

Given these observations, we propose a potential mechanism for the Ir-catalyzed cross-coupling of allylic carbonates and α-aryl diazo esters in which NEt_3_ acts as a nucleophile to activate the pre-catalyst **1** (Fig. 8). Activation generates a coordinatively unsaturated Ir species that can engage the diazo compound to generate an Ir-carbene or react with allylic carbonate to form off-cycle species (**1′** in Fig. 2). The carbene intermediate could undergo rate-determining oxidative addition of the allylic carbonate to generate an η^1^-allyl/carbene intermediate. Migratory insertion followed by β-hydride elimination generates the diene product and liberates free catalyst. *Z*-selectivity could arise from the large coordination sphere of the catalyst to influence elimination from an otherwise unfavorable conformer, or alternatively base-induced anti-elimination could occur. The allylic leaving group impacts *Z*-selectivity (Table 1B), indicative that the resulting *tert*-butoxide or diethyl phosphate anion may play a role in selectivity determining elimination in a potential E2-type pathway.

## Conclusions

In summary, we have reported the development and mechanistic features of an Ir-catalyzed cross-coupling reaction between allylic carbonates and α-aryl diazo esters to form substituted *Z*,*E*-dienoates. *Z*-α-aryl acrylates with sterically demanding ester groups can be generated with good selectivity, suggesting this new reactivity mode may serve as a stereoselective complement to other metal-catalyzed carbene cross-coupling reactions. The *Z*,*E*-diene products are not readily accessible *via* traditional modifications of carbonyl olefination methods. Use of an Ir–allyl (pre)catalyst containing a cyclometalated ligand is essential for product formation which undergoes activation with amine base. Allylic carbonate oxidative addition appears to be the rate determining step, while catalyst deactivation processes help to explain the necessity for relatively high catalyst loadings. While the exact origin of *Z*-selectivity is not well understood at this stage, the steric demand of the ester group and the nature of the allylic leaving group, which likely plays a role as the terminal base, significantly influence the selectivity of the bond-forming process. We believe these studies set the stage for future development in the area of Ir-catalyzed carbene coupling reactions and may have broader implications in designing *Z*-selective Heck-type reactions.

## Conflicts of interest

There are no conflicts to declare.

## Supplementary Material

Supplementary information
