# Supplementary material for: Z-Selective iridium-catalyzed cross-coupling of allylic carbonates and α-diazo esters
Source: Chem Sci. 2017 Oct 24;9(1):238–44. doi: 10.1039/c7sc04283c (PMC5869292; doi:10.1039/c7sc04283c)

Electronic Supplementary Material (ESI) for Chemical Science.
This journal is © The Royal Society of Chemistry 2017

Supporting Information for

Z-Selective Iridium-Catalyzed Cross-Coupling of Allylic Carbonates and α-Diazo Esters

Bryce N. Thomas, Patrick J. Moon, Shengkang Yin, Alex Brown, Rylan J. Lundgren*

Department of Chemistry, University of Alberta, Edmonton, Alberta, T6G 2G2, Canada

rylan.lundgren@ualberta.ca

Contents

**I.**	**General Considerations**	**S-2**
**II.**	**Selected Additional Optimization Data and Scope**	**S-3**
**III**	**Allyl Competition Studies**	**S-5**
**IV.**	**Kinetic Experiments**	**S-6**
**V.**	**Synthesis of Catalyst 1**	**S-11**
**VI.**	**Cross-Coupling of α-Aryl Diazo Esters and Allylic Carbonates: Procedure, Characterization Data and Derivitizations**	**S-12**
**VII.**	**Computational Methods**	**S-23**

I. General Considerations

Unless noted, all reactions were conducted under inert atmosphere employing standard schlenk technique or by the use of an N_2-filled glovebox. All glassware was oven-dried prior to use. Flash chromatography was performed as described by Still and co-workers[S1] (SiliaFlash P60, 40-63μm, 60A silica gel, Silicycle) or by automated flash chromatography (Isolera, HP-SIL or Ultra SNAP silica cartridges, Biotage). Analytical thin-layer chromatography was performed using glass plates pre-coated with silica (SiliaPlate G TLC - Glass-Backed, 250μm, Silicycle). TLC plates were visualized by UV light and/or staining with aqueous basic potassium permanganate. Unless otherwise noted, all reagents were obtained from commercial vendors and used as supplied. Allylic carbonates [S2] and α-aryl diazo esters[S3] were prepared by reported protocols. The Still-Gennari phoshonate was prepared according to established procedures.[S4] Reaction progress kinetic analysis experiments were conducted by removing small aliquots of reaction mixtures containing an internal standard, diluting with $CDCl_3$ and analyzing by 1H NMR (700 MHz).

[S1] W. C. Still, M. Kahn, A. Mitra *J. Org. Chem.* **1978**, *43*, 2923-2925.

[S2] Y. Sumida, S. Hayashi, K. Hirano, H. Yorimitsu, K. Oshima, *Org. Lett.* **2008**, *10*, 1629-1632.

[S3] E. C. Lee, G. C. Fu *J. Am. Chem. Soc.* **2007**, *129*, 12066-12067.

[S4] (a) L. Gavara, C. Petit, J. L. Montchamp *Tetrahedron Lett.* **2012**, *53*, 5000-5003; (b) C. M. Mapes, N. S. Mani, X. Deng, C. R. Pandit, K. J. McClure, M. C. W. Pippel, C. A. Sehon, L. Gomez, S. Shinde, J. G. Breitenbucher, R. K. Jones *J. Org. Chem.* **2010**, *75*, 7950–7953.

II. Selected Additional Optimization Data

Reaction Development S1: Base

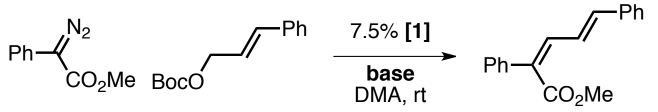

entry	base	yield (%) [*Z,E* :*E,E*]
**1**	**NEt_3**	**31 [61:39]**
2	N–Methylpyrrolidine (NMe-P)	21 [58:42]
3	$HNEt_2$	<2
4	Lutidine	<2
5	Pyridine	<2
6	DABCO	<2
7	DBU	<2
8	K_2CO_3	<2

2.0 equiv cinnamylOBoc, 3.0 equiv base, 18 h, 0.4 M. Yields determined by calibrated GC

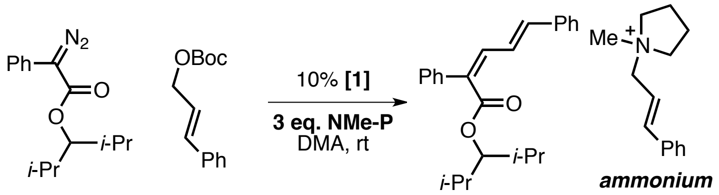

Time (h)	electrophile (% conv.)	ammonium (%)	Yield (%)
1	150	145	0
27	150	122	14

1.5 equiv cinnamylOBoc, 3.0 equiv N-methylpyrrolidine (NMe-P), 0.4 M. Yields determined by ^{1}H NMR using trimethoxybenzene as internal standard.

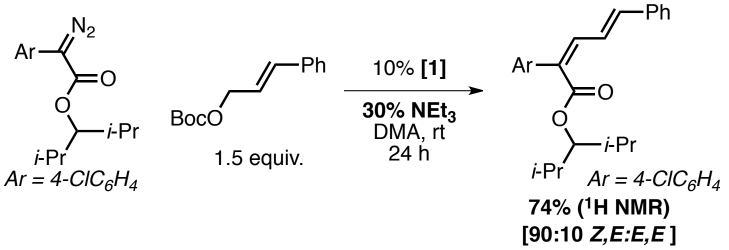

Reaction Development S2: Solvent

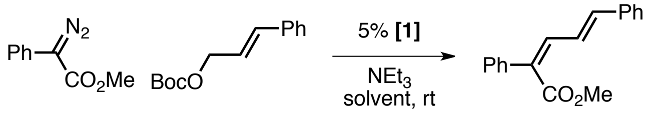

entry	solvent	yield (%) [*Z,E* :*E,E*]
**1**	**DMA**	**34 [64:36]**
2	DMF	27 [57:43]
3	DCE	14 [60:40]
4	DMSO	2
5	NMP	2
6	PhCl	10 [58:42]
7	Dioxane	9 [57:43]
8	Toluene	9 [53:47]
9	DCM	12 [53:47]

2.0 equiv cinnamylOBoc, 3.0 equiv base, 18 h, 0.4 M. Yields determined by calibrated 1H NMR

Reaction Development S3: Concentration

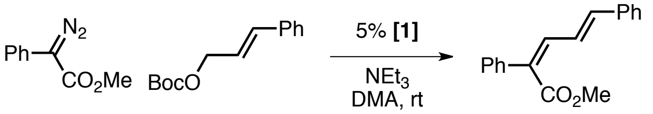

entry	concentration [M]	yield (%) [*Z,E* :*E,E*]
1	1.0	47 [64:36]
2	0.4	41 [64:36]
3	0.2	23 [65:35]
4	0.1	12 [60:40]

2.0 equiv cinnamylOBoc, 3.0 equiv base, 21 h. Yields determined by calibrated 1H NMR

Unsuccessful Diazo Partners (<20% Yield)

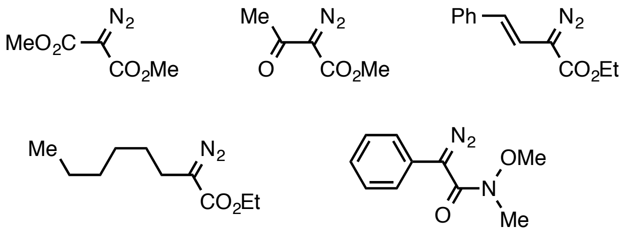

Unsuccessful Allylic Electrophiles (<20% Yield)

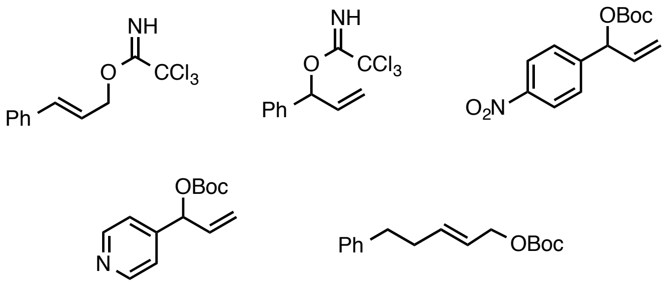

III. Allyl Competition/Crossover Studies

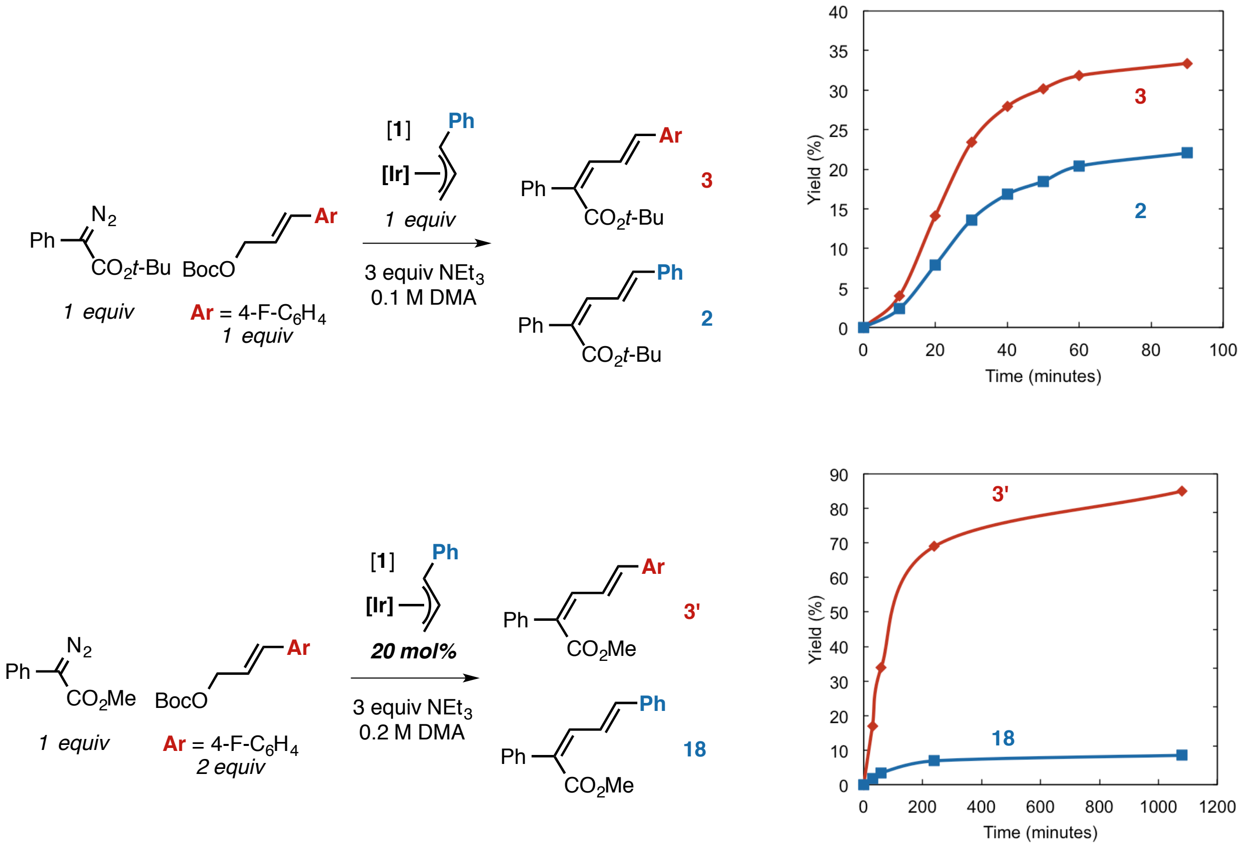

Fig S1: At high loadings of **1**, significant production formation from the initially bound allyl fragment is observed. While **1** is not the active catalyst and does not react directly with α-diazo ester, the cinnamyl group can enter the product forming cycle. Over the course of these experiments carbonate cross-over also occurs (observation of cinnmyl OBoc), which provides a pathway for cinnamyl product formation in the above reactions.

IV. Kinetic Experiments

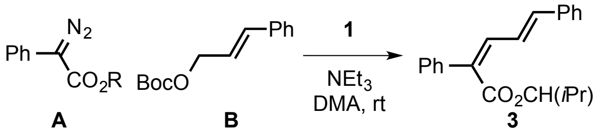

"std. cond."

[A] = 0.26 M [B] = 0.39 M [1] = 0.026 M [NEt_3] = 0.78 M

Fig S2. Variation in Diazo (**A**): Variable time normalization plots for reactions with different initial concentrations of α-diazo ester fit with approximately zero order rate dependence. A value of -0.2 provides the best fit, suggesting diazo may be involved in catalyst decomposition pathways.

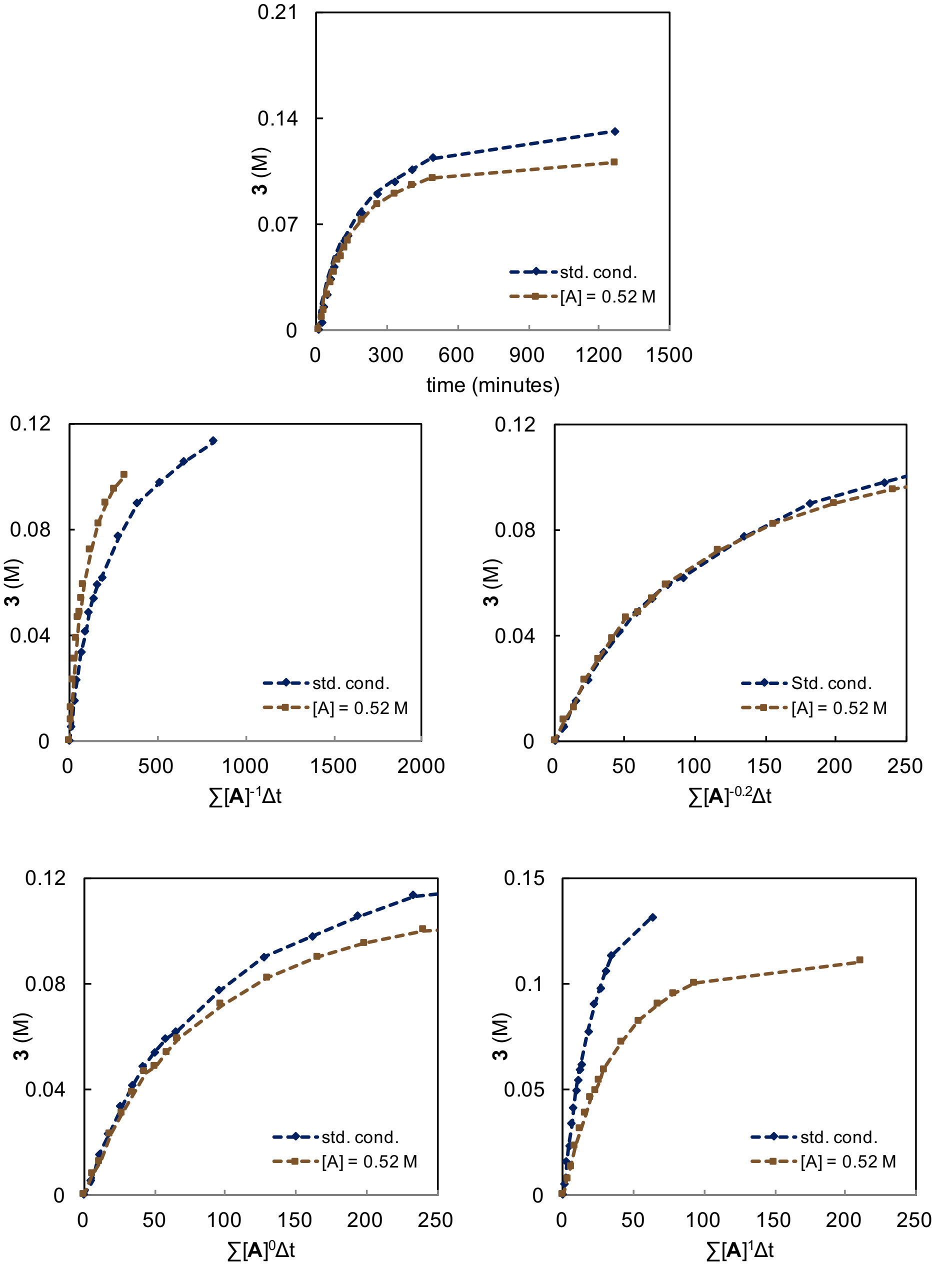

Fig S3. Variation in Allylic Carbonate (**B**): Variable time normalization plots for reactions with different initial concentrations of allylic carbonate fit with approximately 0.5 order rate dependence.

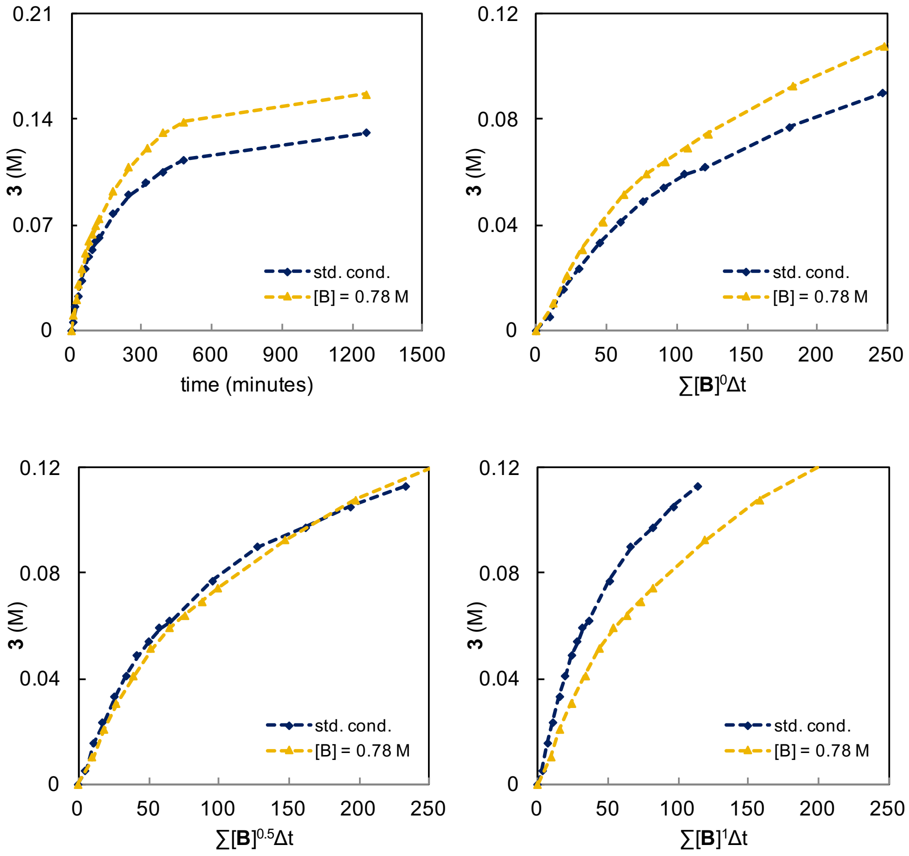

Fig S4. Variation in NEt_3: Variable time normalization plots for reactions with different initial concentrations of NEt_3 fit with approximately zero order rate dependence. Reactions with catalytic NEt_3 (30 mol%) provide similar results.

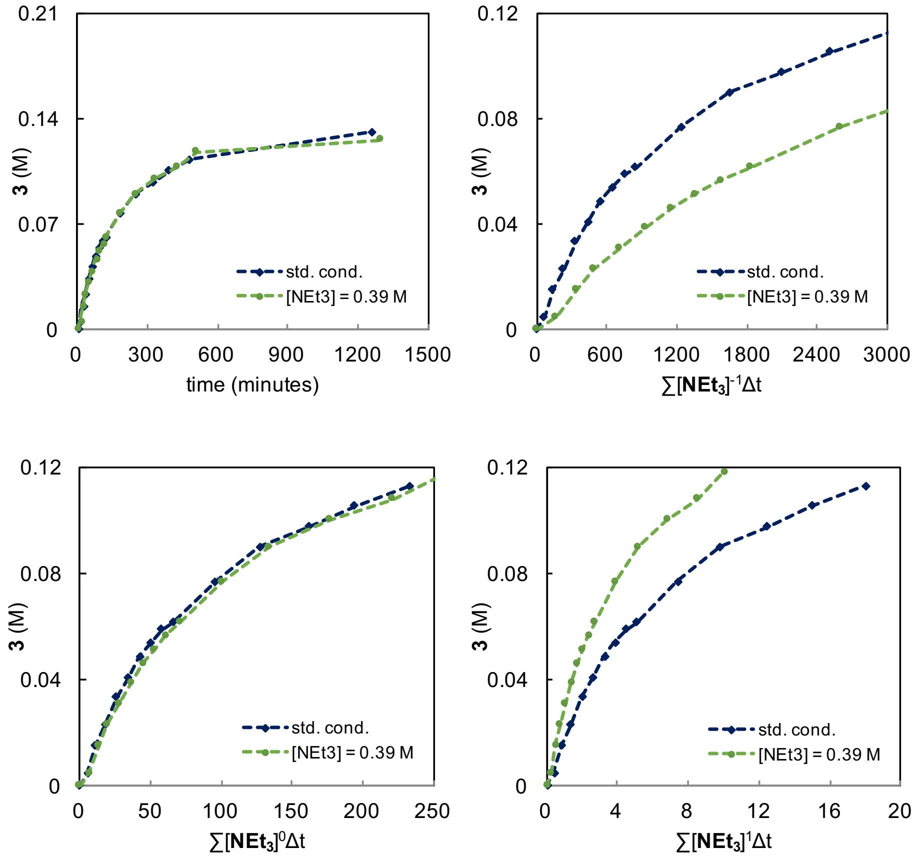

Fig S5. Variation in **1** (precatalyst): Variable time normalization plots for reactions with different initial concentrations of **1** fit with approximately first order rate dependence only in the initial periods of the reaction.

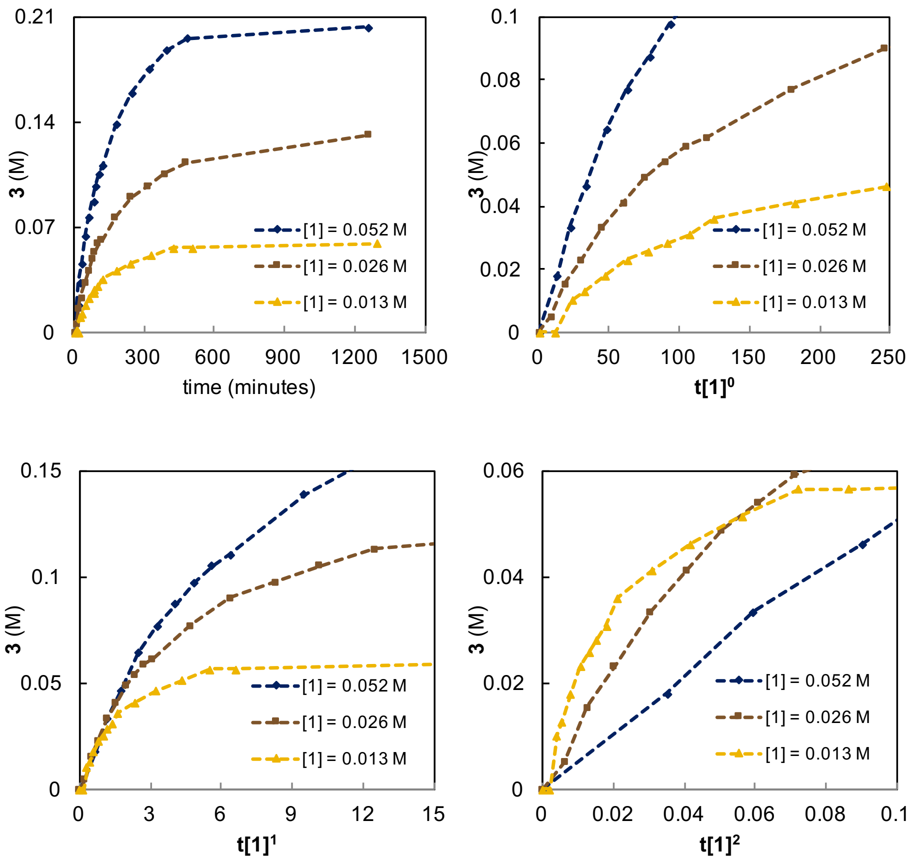

Fig S6. Evidence of Catalyst Deactivation: When comparing the standard reaction (blue trace) to an experiment with initial concentrations of all species equal to that of the standard reaction at 50% conversion, overlay between the rate of consumption of A is not observed (yellow trace, time shifted plot of green trace). When the same experiment was conducted with additional product (0.09 M), the plots overlay. From these experiments, it can be determined that the concentration of active catalyst decreases over the course of the reaction and the inhibition of the catalyst by product does not occur.

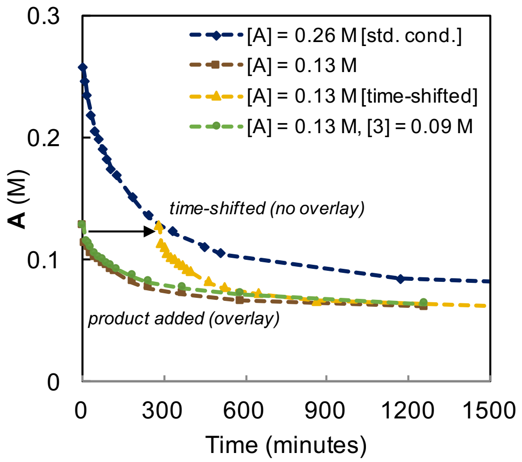

V. Synthesis of Catalyst 1

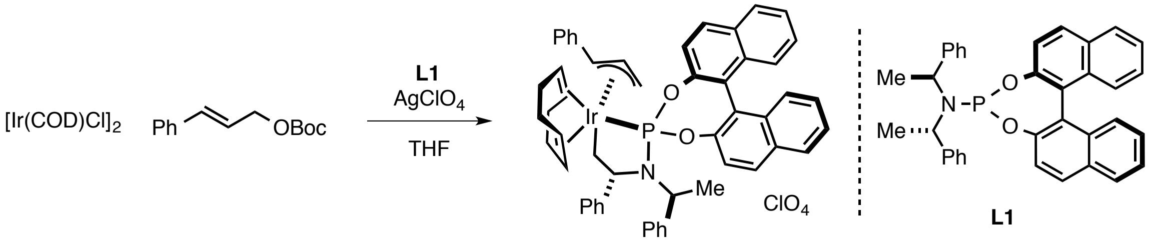

In a N_2-filled glovebox $[Ir(COD)Cl]_2$ (0.5 equiv.), O,O'-(S)-(1,1'-dinaphthyl-2,2'-diyl)-N,N-di-(S,S)-[2-phenylethylphosphoramidite] (1 equiv., prepared according to literature procedure)[5] and THF (12 mL/mmol) were added to a 4 dram vial. Cinnamyl *tert*-butyl carbonate was added as a solution in THF and the mixture was stirred for 2 minutes. $AgClO_4$ was added as a solution in THF and the reaction mixture was stirred at room temperature until complete conversion of the ligand was observed by ^{31}P NMR spectroscopy. The reaction mixture was removed from the glovebox, filtered through Celite and concentrated to a viscous brown oil. The material was triturated by adding Et_2O, (7 mL) followed by pentane (7 mL) and the resulting yellow solid crushed against the walls of the vial. The supernatant was removed, additional Et_2O (7 mL) added and the solid crushed again. The solid was re-dissolved in a minimum of CH_2Cl_2 and the above trituration steps repeated twice more. After decanting the supernatant for the final time the solid yellow powder was dried under high vacuum. Isolated yields greater than 90% are typically achieved by this method. The spectroscopic data agreed with that reported for analogous compounds.

^{1}H NMR ($CDCl_3$, 700 MHz) δ 8.29 (d, *J* = 8.8 Hz, 1H), 8.23 (d, *J* = 8.8 Hz, 1H), 8.06 (d, *J* = 8.2 Hz, 1H), 8.04 (d, *J* = 8.3 Hz, 1H) 7.84 (d, *J* = 8.8 Hz, 1H), 7.74 (d, *J* = 8.8 Hz, 1H), 7.60 (d, *J* = 7.6 Hz, 1H), 7.54 – 7.59 (m, 2H), 7.45 – 7.50 (m, 2H), 7.34 – 7.44 (m, 10H), 7.27 – 7.34 (m, 3H), 7.08 – 7.12 (m, 2H), 5.78 (t, *J* = 12.0 Hz, 1H), 5.26 (m, 1H), 4.76 (m, 1H), 4.04 (m, 1H), 3.85 – 3.96 (m, 3H), 2.93 – 2.93 (m, 2H), 2.75 (m, 1H), 2.54 (m, 1H), 2.45 (d, *J* = 10.9 Hz, 1H), 2.38 (m, 1H), 2.15 (m, 1H), 2.07 (dd, *J* = 12.7, 8.8 Hz, 1H), 1.77 (m, 1H), 1.48 – 1.72 (m, 5H), 1.11 (t, *J* = 12.2 Hz, 1H), 0.60 (d, *J* = 7.3 Hz, 3H);

^{13}C NMR ($CDCl_3$, 126 MHz) δ 148.6, 148.5, 147.4 (2), 142.6, 142.5, 139.9, 134.1, 134.0, 133.0, 132.6, 132.5, 131.9, 131.8, 131.5, 129.6, 129.5, 129.2, 128.9, 128.6, 128.5, 128.1, 127.5, 127.3, 127.1 (2), 126.4, 126.0, 122.8, 122.7, 121.4 (2), 120.8, 101.9, 97.3, 92.9, 89.7, 86.7, 86.5, 83.1, 65.7, 65.5, 60.3, 40.6, 34.8, 33.1, 28.1, 26.7, 18.4, 17.1;

^{31}P NMR ($CDCl_3$, 162 MHz) δ 119.6;

HRMS (LCMS ESI): calcd for $C_{53}H_{50}IrNO_2P$ $[M]^+$: 956.3203. Found 956.3215.

VI. Cross-Coupling of α-Aryl Diazo Esters and Allylic Carbonates

General Procedure 1 To a two dram vial containing a stirbar was added allyl *tert*-butyl carbonate (0.53 mmol, 1.5 equiv.) and **1** (0.035 mmol, 0.10 equiv.) as a 1 mL DMA solution. After stirring 5 minutes, the α-aryl diazo ester (0.35 mmol, 1.0 equiv.) was added. The resulting mixture was stirred for 2 minutes and triethylamine (1.05 mmol, 3.0 equiv.) was added. The vial was sealed with a PTFE lined septa cap, removed from the glovebox and stirred 14 to 36 hours, over which time the solution turned from orange to dark brown. Pressure develops within the vial, a headspace approximately 3 times the solution volume is necessary to prevent significant pressure buildup. Crude conversion and yield were judged by ^{1}H NMR analysis (600 or 700 MHz instrument) of a 5 μL aliquot diluted with $CDCl_3$. The DMA was removed by diluting with EtOAc (20 mL), washing with H_2O (2 x 20 mL) and brine (20 mL), followed by drying over Na_2SO_4. The mixture was concentrated and purified by column chromatography. In some cases, unreacted *tert*-butyl allylic carbonate was not readily separable from the product. In these cases, the solvent was removed and the crude reaction mixture was placed under N_2 and dissolved in anhydrous THF (1 mL). **1** (3.7 mg or approximately 0.01 equiv.) was added as a solution in THF (~100 μL) and the mixture was stirred for 5 minutes. $HNEt_2$ (3 equiv.) was added by syringe and the mixture was stirred for 4 hours allowing for facile separation (General Procedure 2). Isolated yields are reported for the combined mixture of *Z,E* and *E,E* isomers, for clarity where possible, the depicted ^{1}H and ^{13}C spectra are of the major isomer.

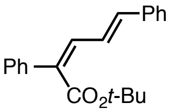

2 Prepared according to the General Procedure 2 from the corresponding α-aryl diazo ester (76.4 mg, 0.35 mmol), *tert*-butyl allylic carbonate (124.1 mg, 0.53 mmol) and **1** (36.9 mg, 0.035 mmol). Isolated in 85% yield, *Z,E*/*E,E* = 87:13, after purification by flash chromatography (Hex/EtOAc gradient) as a light brown oil. High product yields can also be obtained with the branched allylic carbonate, rapid conversion to the linear carbonate isomer is observed in these cases.

^{1}H NMR ($CDCl_3$, 700 MHz) δ 7.53 (dd, *J* = 11.0, 15.8 Hz, 1H), 7.46 – 7.50 (m, 2H), 7.41 – 7.45 (m, 2H), 7.32 – 7.38 (m, 4H), 7.27 – 7.32 (m, 2H), 6.78 – 6.85 (m, 2H), 1.57 – 1.69 (m, 9H);

^{13}C NMR ($CDCl_3$, 176 MHz) δ 167.4, 138.1, 138.0, 137.0, 135.1, 134.9, 128.8, 128.5, 128.3, 127.7, 127.3, 127.0, 125.7, 81.9, 28.4;

HRMS (EI): calcd for $C_{21}H_{22}O_2$ $[M]^+$ 306.1620, found 306.1623.

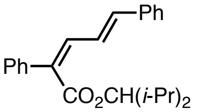

3 Prepared according to the General Procedure 2 from the corresponding α-aryl diazo ester (78 mg, 0.30 mmol), *tert*-butyl allylic carbonate (103 mg, 0.45 mmol) and **1** (31 mg, 0.030

mmol). Isolated in 71% yield, *Z,E*/*E,E* = 90:10, after purification by flash chromatography (pentane/Et_2O gradient) as thick colorless oil.

In a 1 mmol scale reaction the catalyst loading could be reduced to 7.5 mol% with higher concentration (0.80 M), pure *Z,E*-product was obtained in 74% yield (257 mg, 0.74 mmol).

^{1}H NMR ($CDCl_3$, 500 MHz) δ 7.99 (dd, *J* = 11.5, 16.0 Hz, 1H), 7.50 (d, *J* = 8.0 Hz, 2H), 7.44 – 7.42 (m, 2H), 7.38 – 7.29 (m, 6H), 6.85 (app. d, *J* = 3.0 Hz, 1H), 6.83 (app. s, 1H), 4.87 (t, *J* = 6.0 Hz, 1H), 1.97 (app sextet, *J* = 6.9 Hz, 2H), 0.97 (d, *J* = 6.9 Hz, 6H), 0.89 (d, *J* = 6.8 Hz, 6H)

^{13}C NMR ($CDCl_3$, 176 MHz) δ 167.6, 139.2, 138.8, 136.8, 133.3, 129.5, 128.7, 128.5, 128.1, 128.0, 127.5, 127.1, 125.9, 83.7, 29.6, 19.8, 17.5;

HRMS (EI): calcd for $C_{24}H_{28}O_2$ $[M]^+$ 348.2089, found 348.2086.

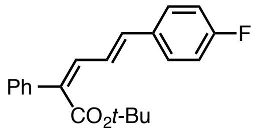

4 Prepared according to the General Procedure 1 from the corresponding α-aryl diazo ester (60.0 mg, 0.27 mmol), *tert*-butyl allylic carbonate (100 mg, 0.10 mmol) and **1** (27 mg, 0.027 mmol). Isolated in 54% yield, *Z,E*/*E,E* >95:5 (92:8 crude), after purification by preparative thin layer chromatography (20:1 Hex/EtOAc) as a pale yellow wax.

^{1}H NMR ($CDCl_3$, 500 MHz) δ 7.50 – 7.40 (m, 5H), 7.37 – 7.34 (m, 2H), 7.31 – 7.27 (m, 1H), 7.05 (t, *J* = 8.8 Hz, 2H), 6.80 – 6.74 (m, 2H), 1.61 (s, 9H);

^{13}C NMR ($CDCl_3$, 176 MHz) δ 167.2, 162.8 (d, J_{CF} = 246 Hz), 138.0, 136.7, 135.0, 134.9, 133.1 (d, J_{CF} = 3 Hz), 128.5 (d, J_{CF} = 8 Hz), 128.2, 127.6, 127.3, 125.4 (d, J_{CF} = 2 Hz), 115.8 (d, J_{CF} = 22 Hz), 81.8, 28.3;

^{19}F NMR ($CDCl_3$, 376 MHz) δ −112.7;

HRMS (EI): calcd for $C_{21}H_{21}O_2F$ $[M]^+$ 324.1526, found 324.1526.

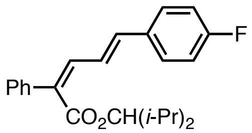

5 Prepared according to the General Procedure 2 from the corresponding α-aryl diazo ester (78 mg, 0.30 mmol), *tert*-butyl allylic carbonate (113 mg, 0.45 mmol) and **1** (31.7 mg, 0.030 mmol). Isolated in 48% yield, *Z,E*/*E,E* = 94:6, after purification by preparative thin layer chromatography.

^{1}H NMR ($CDCl_3$, 600 MHz) δ 7.71 (dd, *J* = 11.4, 15.6 Hz, 1H), 7.48 – 7.44 (m, 2H), 7.42 – 7.39 (m, 2H), 7.36 – 7.33 (m, 2H), 7.31 – 7.29 (m, 1H), 7.04 (t, *J* = 9.0 Hz, 2H), 6.82 – 6.78 (m, 2H), 4.85 (t, *J* = 6.4 Hz, 1H), 1.95 (app. sextet *J* = 6.7 Hz, 2H), 0.96 (d, *J* = 6.9 Hz, 6H), 0.87 (d, *J* = 6.7 Hz, 6H);

^{13}C NMR ($CDCl_3$, 151 MHz) δ 167.5, 162.9 (d, *J* = 249 Hz), 138.8, 137.9, 133.3, 133.0 (d, *J* = 3.1 Hz), 128.7, 128.6, 128.1, 128.0, 127.6, 125.6 (d, *J* = 2.4 Hz), 115.7 (d, *J* = 21.9 Hz), 83.7, 29.5, 19.7, 17.4;

^{19}F NMR ($CDCl_3$, 376 MHz) δ –112.4;

HRMS (EI): calcd for $C_{24}H_{27}FO_2$ $[M]^+$ 366.1995, found 366.1994.

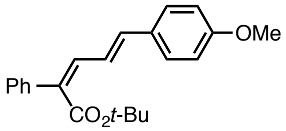

6 Prepared according to the General Procedure 1 from the corresponding α-aryl diazo ester (76.4 mg, 0.35 mmol), *tert*-butyl allylic carbonate (140 mg, 0.53 mmol) and **1** (36.9 mg, 0.035 mmol). Isolated in 63% yield, *Z,E*/*E,E* = 85:15, after purification by flash chromatography (Hex/EtOAc gradient) as pale yellow oil.

^{1}H NMR ($CDCl_3$, 700 MHz) δ 7.38 – 7.50 (m, 5H), 7.32 – 7.38 (m, 2H), 7.29 (m, 1H), 6.87 – 6.93 (m, 2H), 6.73 – 6.82 (m, 2H), 3.83 (s, 3H), 1.62 (s, 9H);

^{13}C NMR ($CDCl_3$, 176 MHz) δ 167.4, 160.0, 138.4, 138.1, 136.1, 133.6, 129.8, 128.4, 128.2, 127.5, 127.4, 123.8, 114.3. 81.6, 55.4, 28.4;

HRMS (EI): calcd for $C_{22}H_{24}O_3$ $[M]^+$ 336.1725, found 336.1728.

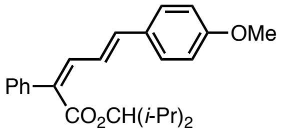

7 Prepared according to the General Procedure 1 from the corresponding α-aryl diazo ester (78 mg, 0.30 mmol), *tert*-butyl allylic carbonate (90 mg, 0.34 mmol) and **1** (31 mg, 0.030 mmol). Isolated in 70% yield, *Z,E*/*E,E* = 90:10, after purification by flash chromatography (Hex/EtOAc gradient) as thick pale yellow oil.

^{1}H NMR ($CDCl_3$, 600 MHz) δ 7.71 (dd, *J* = 11.4, 15.6 Hz, 1H), 7.46 – 7.41 (m, 4H), 7.36 (t, *J* = 7.8 Hz, 2H), 7.31 (tt, *J* = 7.2, 1.3 Hz, 1H), 6.90 (d, *J* = 8.8 Hz, 2H), 6.85 – 6.78 (m, 2H), 4.87 (t, *J* = 6.2 Hz, 1H), 3.85 (s, 3H), 1.98 (app. sextet *J* = 6.6 Hz, 2H), 0.98 (d, *J* = 6.8 Hz, 6H), 0.91 (d, *J* = 6.6 Hz, 6H);

^{13}C NMR ($CDCl_3$, 151 MHz) δ 167.7, 160.4, 139.9, 139.2, 139.1(2), 126.6, 128.6, 128.3, 128.0, 127.4, 124.0, 114.2, 83.6, 55.4, 29.6, 19.8, 17.5;

HRMS (ESI): calcd for $C_{25}H_{30}O_3Na[M+Na]^+$ 401.2087, found 401.2082.

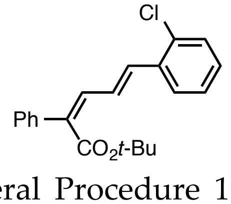

8 Prepared according to the General Procedure 1 from the corresponding α-aryl diazo ester (87.2 mg, 0.40 mmol), *tert*-butyl allylic carbonate (161 mg, 0.60 mmol) and **1** (63.3 mg, 0.060 mmol). Isolated in 78% yield, *Z,E*/*E,E* = 82:18, after purification by flash chromatography (40:1 to 20:1 Hex/EtOAc step gradient) as pale yellow oil.

^{1}H NMR ($CDCl_3$, 700 MHz) δ 7.64 (dd, *J* = 7.9, 1.5 Hz, 1H), 7.49 (dd, *J* = 15.6, 11.0 Hz, 1H), 7.42 – 7.45 (m, 2H), 7.32 – 7.40 (m, 4H), 7.30 (m, 1H), 7.19 – 7.23 (m, 2H), 6.86 (d, *J* = 11.3 Hz, 1H), 1.60 (s, 9H);

^{13}C NMR ($CDCl_3$, 126 MHz) δ 167.2, 137.7, 136.0, 134.9, 134.5, 133.4, 129.9, 129.2, 128.3, 127.9, 127.8, 127.2 (2), 126.9, 126.7, 82.0, 28.3;

HRMS (EI): calcd for $C_{21}H_{21}ClO_2$ $[M]^+$ 340.1230, found 340.1234.

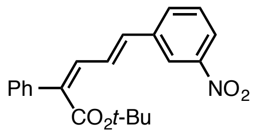

9 Prepared according to the General Procedure 1 from the corresponding α-aryl diazo ester (76.4 mg, 0.35 mmol), *tert*-butyl allylic carbonate (148 mg, 0.53 mmol) and **1** (36.9) mg, 0.035 mmol). 50% yield by ^{1}H NMR, isolated in 39% yield, *Z,E*/*E,E* = 84:16, after purification by flash chromatography (Hex/EtOAc gradient) as pale yellow oil.

^{1}H NMR ($CDCl_3$, 700 MHz) δ 8.30 (t, *J* = 2.1 Hz, 1H), 8.12 (m, 1H), 7.77 (d, *J* = 7.7 Hz, 1H), 7.61 (dd, *J* = 7.7, 15.4 Hz, 1H), 7.53 (m, 1H), 7.45 – 7.43 (m, 2H), 7.39 – 7.35 (m, 2H), 7.30 – 7.28 (m, 1H), 6.83 (d, *J* = 15.4 Hz, 1H), 6.80 (d, *J* = 11.2 Hz, 1H), 1.62 (s, 9H);

^{13}C NMR ($CDCl_3$, 176 MHz) δ 167.0, 148.7, 138.7, 137.4, 137.2, 134.6, 133.3, 132.2, 130.3, 129.6, 128.4, 128.1, 127.2, 122.6, 121.4, 82.3, 28.3;

HRMS (EI): calcd for $C_{21}H_{21}O_4N$ $[M]^+$ 351.1471, found 351.1474

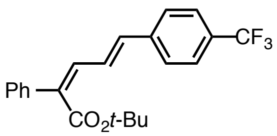

10 Prepared according to the General Procedure 1 from the corresponding α-aryl diazo ester (76.4 mg, 0.35 mmol), *tert*-butyl allylic carbonate (160.2 mg, 0.53 mmol) and **1** (36.9 mg, 0.035 mmol). Isolated in 74% yield, *Z,E*/*E,E* = 81:19 after purification by flash chromatography (Hex/Et_2O gradient) as pale yellow oil.

^{1}H NMR ($CDCl_3$, 700 MHz) δ 7.58 – 7.63 (m, 2H), 7.51 – 7.58 (m, 3H), 7.40 – 7.45 (m, 2H), 7.34 – 7.40 (m, 2H), 7.32 (m, 1H), 6.77 – 6.84 (m, 2H), 1.61 (s, 9H);

^{13}C NMR ($CDCl_3$, 176 MHz) (*E,Z* and *E,E* isomers) δ 167.3 (*Z,E*), 166.5 (*E,E*), 140.5, 138.5, 137.8, 137.7, 136.8, 136.1, 135.4, 134.0, 130.6, 130.5, 130.2, 130.0, 128.9, 128.5, 128.2, 128.1, 127.9, 127.7, 127.4 (2), 127.1, 125.9 (4), 125.8, 125.4, 123.2, 82.4 (*Z,E*), 81.3 (*E,E*), 28.6 (*Z,E*), 28.3 (*E,E*);

^{19}F NMR ($CDCl_3$, 377MHz) δ −62.6 (*Z,E*), −62.7 (*E,E*);

HRMS (EI): calcd for $C_{22}H_{21}F_3O_2$ $[M]^+$ 374.1494, found 374.1489.

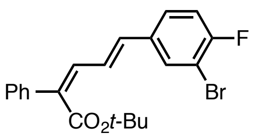

11 Prepared according to the General Procedure 2 at 35 °C from the corresponding α-aryl diazo ester (76.4 mg, 0.35 mmol), *tert*-butyl allylic carbonate (175.5 mg, 0.53 mmol) and **1** (36.9 mg, 0.035 mmol). Isolated in 69% yield, *Z,E*/*E,E* = 85:15, after purification by flash chromatography (30:1 pentane/Et_2O) as a colorless oil.

^{1}H NMR ($CDCl_3$, 700 MHz) δ 7.63 (dd, J = 6.6, 2.2 Hz, 1H), 7.27 – 7.49 (m, 7H), 7.10 (m, 1H), 6.64 – 6.78 (m, 2H), 1.60 (s, 9H);

^{13}C NMR ($CDCl_3$, 176 MHz) δ 167.1, 158.9 (J_{CF} = 249.6 Hz), 137.8, 135.8, 135.1, 134.7 (J_{CF} = 4.1 Hz), 134.5, 131.8, 128.3, 127.9, 127.3, 127.2 (J_{CF} = 7.2 Hz), 126.6 (J_{CF} = 2.5 Hz), 116.8 (J_{CF} = 22.9 Hz), 109.5 (J_{CF} = 21.5 Hz), 82.0, 28.3;

^{19}F NMR ($CDCl_3$, 176 MHz) δ – 107.4 (*Z,E*), −106.7 (*E,E*);

HRMS (EI): calcd for $C_{21}H_{20}O_2FBr$ $[M]^+$ 404.0599, found 404.0603.

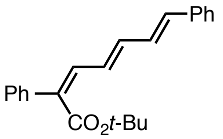

12 Prepared according to the General Procedure 1 from the corresponding α-aryl diazo ester (76.4 mg, 0.35 mmol), *tert*-butyl allylic carbonate (138.0 mg, 0.53 mmol) and **1** (60 mg, 0.056 mmol). 54% yield of *Z,E* product by calibrated ^{1}H NMR (no *E,E* product observed), product was not readily separable from unreacted *tert*-butyl allylic carbonate, 17% yield obtained by recrystallization of the crude mixture with cold pentane.

^{1}H NMR ($CDCl_3$, 500 MHz) δ 7.45 (d, J = 7.5 Hz, 2H), 7.43 – 7.40 (m, 2H), 7.37 – 7.33 (m, 4H), 7.31 – 7.25 (m, 2H), 7.08 (dd, J = 11.5, 15.0 Hz, 1H), 6.95 (dd, J = 11.0, 15.0 Hz, 1H), 6.76 – 6.63 (m, 3H) 1.61 (s, 9H);

^{13}C NMR ($CDCl_3$, 126 MHz) δ 167.3, 138.7, 138.0, 137.0, 135.0 (2), 134.5, 129.8, 128.9, 128.7, 128.2, 128.0, 127.6, 127.3, 126.7, 81.2, 28.3;

HRMS (EI): calcd for $C_{23}H_{24}O_2$ $[M]^+$ 332.1776, found 332.1774.

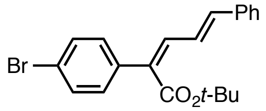

14 Prepared according to the General Procedure 2 from the corresponding α-aryl diazo ester (89.1 mg, 0.30 mmol), *tert*-butyl allylic carbonate (105.3 mg, 0.45 mmol) and **1** (31.0 mg, 0.030 mmol). Isolated in 95% yield, *Z,E*/*E,E* = 87:13, after purification by flash chromatography (Hex/EtOAc gradient) as thick pale yellow oil.

^{1}H NMR ($CDCl_3$, 500 MHz) δ 7.60 – 7.53 (m, 1H), 7.50 – 7.46 (m, 3H), 7.37 (t, J = 8.0 Hz, 2H), 7.32 – 7.27 (m, 4H), 6.84 (d, J = 15.5 Hz, 1H), 6.79 (d, J = 11.5 Hz, 1H), 1.61 (s, 9H);

^{13}C NMR ($CDCl_3$, 176 MHz) δ 166.7, 139.0, 137.2, 136.7, 136.3, 133.4, 131.3, 129.0, 128.8, 128.6, 127.0, 125.5, 121.7, 82.0, 28.3;

HRMS (EI): calcd for $C_{21}H_{21}BrO_2$ $[M]^+$ 386.0704, found 386.0708.

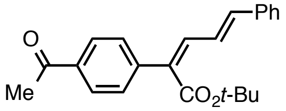

15 Prepared according to the General Procedure 1 from the corresponding α-aryl diazo ester (91 mg, 0.35 mmol), *tert*-butyl allylic carbonate (124 mg, 0.53 mmol) and **1** (37 mg, 0.035

mmol). Isolated in 55% yield, *Z,E*/*E,E* = 95:5 (crude *Z,E*/*E,E* >90:10) , after purification by flash chromatography (10:1 Hex/EtOAc) as pale yellow oil.

^{1}H NMR ($CDCl_3$, 600 MHz) δ 7.96 (d, *J* = 8.4 Hz, 2H), 7.59 (dd, *J* = 11.4, 15.6 Hz, 1H), 7.53 – 7.49 (m, 4H), 7.39 (t, *J* = 7.2 Hz, 2H), 7.33 – 7.30 (m, 1H), 6.91 – 6.88 (m, 2H), 2.63 (s, 3H), 1.63 (s, 9H);

^{13}C NMR ($CDCl_3$, 151 MHz) δ 197.6, 166.7, 142.9, 139.7, 137.3, 136.6, 136.0, 133.6, 128.8 (2), 128.2, 127.5, 127.1, 125.4, 82.2, 28.3, 26.6;

HRMS (EI): calcd for $C_{23}H_{24}O_3$ [M]$^+$ 348.1726, found 348.1722.

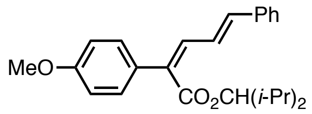

16 Prepared according to the General Procedure 1 from the corresponding α-aryl diazo ester (102 mg, 0.35 mmol), *tert*-butyl allylic carbonate (123 mg, 0.53 mmol) and **1** (36.9 mg, 0.035 mmol). Isolated in 89% yield, *Z,E*/*E,E* = 91:9, after purification by flash chromatography (pentane/Et_2O gradient) as a pale yellow oil.

^{1}H NMR ($CDCl_3$, 600 MHz) δ 7.71 (dd, *J* = 11.3, 15.5 Hz, 1H), 7.45 – 7.49 (m, 2H), 7.31 – 7.38 (m, 4H), 7.27 (m, 1H), 6.87 – 6.90 (m, 2H), 6.75 – 6.82 (m, 2H), 4.87 (t, *J* = 6.2 Hz, 1H), 3.83 (s, 3H), 1.97 (app sextet, *J* = 6.5 Hz, 2H), 0.97 (d, *J* = 7.0 Hz, 6H), 0.90 (d, *J* = 7.0 Hz, 6H).

^{13}C NMR ($CDCl_3$, 125 MHz) δ 168.1, 159.4, 138.5, 137.2, 137.1, 133.2, 131.4, 129.4, 128.8, 128.5, 127.2, 126.2, 113.7, 83.9, 55.4, 29.7, 19.9, 17.7.

HRMS (ESI): calcd for $C_{25}H_{30}NaO_3$ [M+Na]$^+$ 401.2087, found 401.2090.

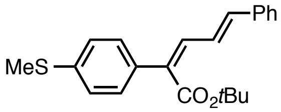

17 Prepared according to the General Procedure 2 from the corresponding α-aryl diazo ester (40 mg, 0.15 mmol), *tert*-butyl allylic carbonate (51 mg, 0.22 mmol) and **1** (24 mg, 0.023 mmol). Isolated in 80% yield, *Z,E*/*E,E* = 80:20, after purification by flash chromatography (Pentane/Et_2O gradient) as pale yellow solid.

^{1}H NMR ($CDCl_3$, 500 MHz) δ 7.54 – 7.47 (m, 3H), 7.39 – 7.36 (m, 4H), 7.31 – 7.24 (m, 3H), 6.81 (d, *J* = 11.5 Hz, 1H), 6.79 (d, *J* = 15.5 Hz, 1H), 2.52 (s, 3H), 1.63 (s, 9H);

^{13}C NMR ($CDCl_3$, 176 MHz) δ 167.2, 138.1, 137.9, 136.9, 134.7, 134.3 (2), 128.8, 128.4, 127.6, 126.9, 126.2, 125.6, 81.9, 28.3, 15.7;

HRMS (EI): calcd for $C_{22}H_{24}O_2S$ [M]$^+$ 352.1497, found 352.1497.

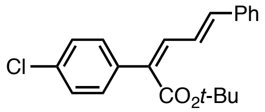

18 Prepared according to the General Procedure 1 from the corresponding α-aryl diazo ester (76 mg, 0.30 mmol), *tert*-butyl allylic carbonate (105.3 mg, 0.45 mmol) and **1** (31.0 mg,

0.030 mmol). Isolated in 60% yield, *Z,E*/*E,E* = 89:11, after purification by flash chromatography (Hex/EtOAc gradient) as pale yellow oil.

^{1}H NMR ($CDCl_3$, 500 MHz) δ 7.57 (dd, *J* = 15.5, 11.5 Hz, 1H), 7.45 – 7.50 (m, 2H) 7.20 – 7.40 (m, 7H), 6.74 – 6.86 (m, 2H), 1.60 (s, 9H);

^{13}C NMR ($CDCl_3$, 126 MHz) δ 166.8, 138.9, 136.8, 136.3, 133.5, 133.4, 131.8, 128.8 (2), 128.7, 128.4, 127.1, 125.5, 82.1, 28.4;

HRMS (EI): calcd for $C_{21}H_{21}ClO_2$ $[M]^+$ 340.1230, found 340.1227.

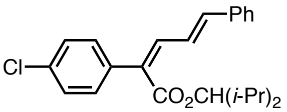

19 Prepared according to the General Procedure 1 from the corresponding α-aryl diazo ester (103 mg, 0.35 mmol), *tert*-butyl allylic carbonate (123 mg, 0.53 mmol) and **1** (36.9 mg, 0.035 mmol). Isolated in 80% yield, *Z,E*/*E,E* = 97:3, after purification by flash chromatography (pentane/Et_2O gradient) as a pale yellow oil.

^{1}H NMR ($CDCl_3$, 500 MHz) δ 7.86 (dd, *J* = 11.2, 15.8 Hz, 1H), 7.51 – 7.55 (m, 2H), 7.27 – 7.43 (m, 7H), 6.83 – 6.92 (m, 2H), 4.91 (t, *J* = 6.2 Hz, 1H), 2.01 (app sextet, *J* = 6.7 Hz, 2H), 1.00 (d, *J* = 6.7 Hz, 6H), 0.93 (d, *J* = 6.7 Hz, 6H).

^{13}C NMR ($CDCl_3$, 125 MHz) δ 167.2, 140.2, 139.8, 137.5, 136.7, 133.6, 131.9, 129.7, 128.85, 128.84, 128.3, 127.3, 125.8, 83.9, 29.7, 19.9, 17.6.

HRMS (ESI): calcd for $C_{24}H_{27}ClNaO_2$ $[M+Na]^+$ 405.1592, found 405.1596.

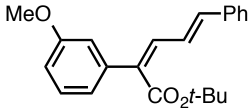

20 Prepared according to the General Procedure 1 from the corresponding α-aryl diazo ester (74 mg, 0.30 mmol), *tert*-butyl allylic carbonate (105 mg, 0.45 mmol) and **1** (30 mg, 0.030 mmol). Isolated in 77% yield, *Z,E*/*E,E* = 86:14, after purification by preparative thin layer chromatography (20:1 pentan/Et_2O) as a colorless waxy solid.

^{1}H NMR ($CDCl_3$, 700 MHz) δ 7.48 – 7.44 (m, 2H), 7.34 (t, *J* = 7.7 Hz, 2H), 7.30 – 7.24 (m, 3H), 7.00 (d, *J* = 7.7 Hz, 1H), 6.96 (t, *J* = 2.8 Hz, 1H), 6.84 (dd, *J* = 2.8, 8.4 Hz, 1H), 6.80 – 6.77 (m, 2H), 3.82 (s, 3H), 1.62 (s, 9H);

^{13}C NMR ($CDCl_3$, 176 MHz) δ 167.2, 159.4, 139.2, 138.1, 136.9, 134.8, 129.2, 128.7 (2), 128.4, 126.9, 125.5, 119.8, 113.4, 112.7, 81.9, 55.2, 28.3;

HRMS (EI): calcd for $C_{22}H_{24}O_3$ $[M]^+$ 336.1726, found 336.1724.

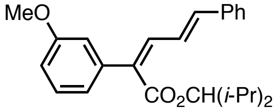

21 Prepared according to the General Procedure 2 from the corresponding α-aryl diazo ester (102 mg, 0.35 mmol), *tert*-butyl allylic carbonate (123 mg, 0.53 mmol) and **1** (36.9 mg,

0.035 mmol). Isolated in 75% yield, *Z,E*/*E,E* = 84:16, after purification by flash chromatography (pentane/Et_2O gradient) as a pale yellow oil.

[1]**H NMR** ($CDCl_3$, 500 MHz) δ 7.76 (dd, *J* = 11.4, 15.5 Hz, 1H), 7.48 – 7.52 (m, 2H), 7.34 – 7.38 (m, 2H), 7.26 – 7.31 (m, 2H), 7.04 (m, 1H), 7.00 (m, 1H), 6.85 – 6.89 (m, 2H), 6.83 (m, 1H), 4.89 (t, *J* = 6.1 Hz, 1H), 3.83 (s, 3H), 1.98 (app sextet, *J* = 6.6, 2H), 0.99 (d, *J* = 6.6 Hz, 6H), 0.91(d, *J* = 6.6 Hz, 6H);

[13]**C NMR (**$CDCl_3$, 125 MHz) δ 167.7, 159.4, 140.2, 139.4, 138.6, 136.9, 133.4, 129.1, 128.8, 128.7, 127.3, 125.9, 120.8, 113.7, 113.5, 83.9, 55.3, 29.7, 19.9, 17.6;

HRMS (ESI): calcd for $C_{25}H_{30}NaO_3$ $[M+Na]^+$ 401.2087, found 401.2087.

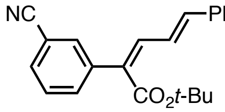

22 Prepared according to the General Procedure 1 at 35 °C from the corresponding α-aryl diazo ester (56.7 mg, 0.23 mmol), *tert*-butyl allylic carbonate (83 mg, 0.35 mmol) and **1** (37 mg, 0.035 mmol). Isolated in 73% yield, *Z,E*/*E,E* = 63:37, after purification by flash chromatography (Hex/EtOAc gradient) as pale yellow oil.

[1]**H NMR** ($CDCl_3$, 700 MHz) δ 7.65 – 7.63 (m, 1H), 7.59 – 7.57 (m, 1H), 7.52 – 7.50 (m, 2H), 7.32 – 7.26 (m, 6H), 6.99 (d, *J* = 14.7 Hz, 1H), 6.65 (dd, *J* = 11.2, 15.4 Hz, 1H); Selected *E,E* signals 6.87 (d, *J* = 15.4 Hz, 1H), 6.81 (dd, *J* = 0.7, 11.2 Hz, 1H) 1.51 (s, 9H);

[13]**C NMR (**$CDCl_3$, 176 MHz) δ 165.6, 141.7, 140.7, 137.0, 136.0, 135.0, 133.0, 131.6, 131.1, 129.2, 128.8, 127.3, 123.7, 118.7, 112.2, 81.5, 28.3, 28.1;

HRMS (EI): calcd for $C_{22}H_{21}NO_2$ $[M]^+$ 331.1572, found 331.1572.

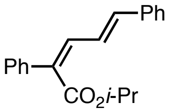

24 Prepared according to the General Procedure 1 from the corresponding α-aryl diazo ester (71.5 mg, 0.35 mmol), *tert*-butyl allylic carbonate (124 mg, 0.50 mmol) and **1** (37 mg, 0.035 mmol). Isolated in 84% yield, *Z,E*/*E,E* = 79:21, after purification by flash chromatography (20:1 pentane/EtO_2) as a pale yellow oil.

[1]**H NMR** ($CDCl_3$, 700 MHz) δ 7.56 (dd, *J* = 15.6, 11.3 Hz, 1H), 7.47 (m, 2H), 7.43 – 7.39 (m, 2H), 7.35 (m, 4H), 7.32 – 7.27 (m, 2H), 6.89 – 6.78 (m, 2H), 5.35 – 5.25 (m, 1H), 1.37 (d, *J* = 6.3 Hz, 6H);

[13]**C NMR (**$CDCl_3$, 176 MHz) δ 167.5, 138.7, 137.9, 136.8, 136.6, 133.6, 128.8, 128.6, 128.3, 127.8, 127.5, 127.1, 125.7, 68.7, 22.0;

HRMS (EI): calcd for $C_{20}H_{20}O_2$ $[M]^+$ 292.1463, found 292.1464.

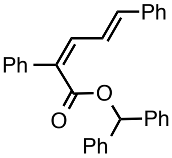

25 Prepared according to the General Procedure 1 from the corresponding α-aryl diazo ester (98.4 mg, 0.30 mmol), *tert*-butyl allylic carbonate (105.3 mg, 0.45 mmol) and **1** (31 mg, 0.030 mmol). Isolated in 75% yield, *Z,E*/*E,E* = 84:16, after purification by flash chromatography (Hex/EtOAc gradient) as thick colorless oil.

^{1}H NMR ($CDCl_3$, 500 MHz) δ 7.52 – 7.25 (m, 20H), 7.21 (s, 1H), 7.02 (m, 1H), 6.94 (d, *J* = 11.4 Hz, 1H), 6.83 (d, *J* = 15.6 Hz, 1H);

^{13}C NMR ($CDCl_3$, 126 MHz) (Mixture of *E,Z* and *E,E* Isomers) δ 166.9, 166.3, 141.0, 140.9, 140.6, 140.1, 139.2, 134.8, 134.6, 136.6, 136.3, 135.2, 132.7, 132.6, 130.5, 129.0, 128.8, 128.6 (2), 128.5, 128.3, 128.0 (2), 127.9, 127.8, 127.7, 127.3, 127.2, 127.1, 125.6, 124.8, 77.6, 77.4;

HRMS (EI): calcd for $C_{30}H_{24}O_2$ $[M]^+$ 416.1776, found 416.1773.

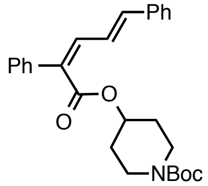

26 Prepared according to the General Procedure 1 from the corresponding α-aryl diazo ester (104 mg, 0.30 mmol), *tert*-butyl allylic carbonate (104 mg, 0.45 mmol) and **1** (31.7 mg, 0.030 mmol). Isolated in 74% yield, *Z,E*/*E,E* = 76:24, after purification by flash chromatography (8:1 to 4:1 Hex/EtOAc gradient) as thick colorless oil.

^{1}H NMR ($CDCl_3$, 500 MHz) δ 7.64 (dd, *J* = 15.5, 11.1 Hz, 1H), 7.45 – 7.51 (m, 2H), 7.26 – 7.42 (m, 6H), 6.82 – 6.90 (m, 2H), 5.21 (m, 1H), 3.57 – 3.73 (m, 2H), 3.23 – 3.34 (m, 2H), 1.90 – 2.02 (m, 2H), 1.62 – 1.79 (m, 2H), 1.54 – 1.59 (m, 2H), 1.45 (s, 9H);

^{13}C NMR ($CDCl_3$, 126 MHz) δ 167.0, 154.7, 139.6, 138.4, 138.2, 136.7, 132.8, 128.8 (2), 128.3, 127.8, 127.7, 127.2, 125.5, 79.8, 70.5, 41.0, 30.7, 28.5;

HRMS (EI): calcd for $C_{27}H_{31}NO_4$ $[M]^+$ 433.2253, found 433.2254.

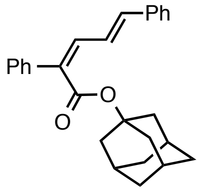

27 Prepared according to the General Procedure 2 from the corresponding α-aryl diazo ester (95 mg, 0.32 mmol), *tert*-butyl allylic carbonate (103 mg, 0.45 mmol) and **1** (31 mg, 0.030 mmol). Isolated in 63% yield, *Z,E*/*E,E* = 84:16, after purification by preparative thin layer chromatogrphy as waxy colorless solid. The product was contaminated with ~4% dioctylphthalate.

^{1}H NMR ($CDCl_3$, 700 MHz) δ 7.55 (dd, *J* = 11.2, 15.4 Hz, 1H), 7.49 (d, *J* = 7.0 Hz, 2H), 7.45 (d, *J* = 9.1 Hz, 2H), 7.38 – 7.35 (m, 4H), 7.31 – 7.29 (m, 2H), 6.82 (d, *J* = 4.9 Hz, 1H), 6.80 (s, 1H), 2.96 (s, 6H), 2.25 (s, 3H), 1.77 – 1.70 (m, 6H);

^{13}C NMR ($CDCl_3$, 176 MHz) δ 167.0, 138.0, 136.9, 134.9 (2), 129.5, 128.7, 128.4, 128.2, 127.6, 127.3, 127.0, 125.8, 82.1, 41.6, 36.2, 30.9;

HRMS (EI): calcd for $C_{27}H_{28}O_2$ $[M]^+$ 384.2089, found 384.2086.

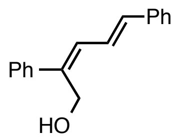

28 To a two-dram vial containing a stirbar was added dienoate **3** (50 mg, 0.14 mmol). The vial was sealed with a PTFE lined septa cap and placed under N_2 before a solution of DIBAL-H (~1.0 M in hexane, 350 μL, 0.34 mmol) was added dropwise to the reaction mixture via syringe. After 30 minutes additional DIBAL was added (100 μL, 0.10 mml). After 10 minutes the reaction was diluted with Et_2O (2 mL) and quenched with aq. KOH (0.50 mL). The mixture was stirred for 5 minutes before $MgSO_4$ was added and the solution was filtered, concentrated and purified by preparative thin layer chromatography (3:1 Hex/EtOAc) to give the *Z*-allylic alcohol in 73% yield (24 mg, 0.10 mmol) as a white solid.

^{1}H NMR ($CDCl_3$, 700 MHz) δ 7.58 (m, 2H), 7.49 (d, *J* = 7.9 Hz, 2H), 7.40 (t, *J* = 7.5 Hz, 2H), 7.36 (t, *J* = 7.2 Hz, 2H), 7.33 – 7.26 (m, 3H), 6.77 (d, *J* = 15.0 Hz, 1H), 6.72 (d, *J* = 11.3 Hz, 1H), 4.82 (d, *J* = 5.1 Hz, 2H), 1.47 (t, *J* = 5.3 Hz, 1H);

^{13}C NMR ($CDCl_3$, 176 MHz) δ 140.3, 139.2, 137.2, 135.5, 130.6, 128.8, 128.1, 127.7, 126.7, 126.2, 124.3, 59.9;

HRMS (EI): calcd for $C_{17}H_{16}O$ $[M]^+$ 236.1201, found 236.1201.

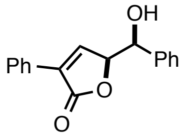

29 To a two-dram vial containing a stirbar was added dienoate **3** (14.0 mg, 0.040 mmol), methyl sulfonamide (18 mg, 0.19 mmol), AD-mix-α (59 mg, ~0.15 mmol), trimethoxybenzene (5.2 mg, 0.031 mmol) as a 1H NMR internal standard. The mixture was dissolved in 0.40 mL water/*t*-BuOH (1:1) as stirred at room temperature for 26 hours. The reaction was diluted with Et_2O (5 mL), quenched with aq. HCl (~0.5 M, 2 mL), extracted with additional Et_2O (3 x 5 mL) and dried over Na_2SO_4. The cyclized product was formed in 62% yield based on calibrated ^{1}H NMR, product was purified by preparative thin layer chromatography (3:1 Hex/EtOAc) to give the lactone in 40% yield (4.0 mg, 0.015 mmol) waxy solid. The product stereochemistry was determined by analogy to that reported by O'Doherty and co-workers.[S5]

^{1}H NMR ($CDCl_3$, 700 MHz) δ 7.78 – 7.76 (m, 2H), 7.43 – 7.41 (m, 4H), 7.39 – 7.37 (m, 4H), 7.22 (d, *J* = 1.8 Hz, 1H), 5.17 (dd, *J* = 1.9, 7.4 Hz, 1H), 4.74 (dd, *J* = 2.6, 7.4 Hz, 1H), 2.73 (d, *J* = 2.8 Hz, 1H);

^{13}C NMR ($CDCl_3$, 176 MHz) δ 171.0, 144.1, 137.8, 133.2, 129.1, 129.0, 128.9, 128.7, 127.1, 126.8, 84.2, 76.2;

HRMS (EI): calcd for $C_{17}H_{12}O_2$ $[M – H_2O]^+$ 248.0837, found 248.0831.

[S5] M. M. Ahmed, H. Cui, G. A. O'Doherty *J. Org. Chem.* **2006**, *71*, 6686-6689.

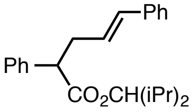

30 To a one dram vial was added **3** (18 mg, 0.052 mmol) and trimethoxybenzene (2.6 mg, 0.0152 mmol) followed by a stock solution of $[Rh(COD)Cl]_2$ and PPh_3 in MeCN (0.20 mL MeCN, Rh: 0.72 mg, 0.0026 mmol, PPh_3 2.3 mg, 0.0078 mmol). The mixture was stirred for five minutes before adding 0.050 mL of a MeCN stock solution containing HCO_2H/NEt_3 (5:2; 0.052 mmol HCO_2H). The vial was sealed with a PTFE lined septa cap and stirred at 35 ˚C. After 5.5 hours 92% conversion of starting material and 85% product was observed by 1H NMR.

^{1}H NMR ($CDCl_3$, 700 MHz) δ 7.36 – 7.35 (m, 2H), 7.32 – 7.29 (m, 2H), 7.27 – 7.23 (m, 5H), 7.18 – 7.16 (m, 1H), 6.44 (d, *J* = 15.8 Hz, 1H), 6.13 (dt, *J* = 15.8, 7.0 Hz, 1H), 4.55 (t, *J* = 6.0 Hz, 1H), 3.71 (dd, *J* = 8.7, 6.6 Hz, 1H), 3.03 (m, 1H), 2.67 (m, 1H), 1.81 (m, 2H), 0.80 (dd, *J* = 6.9, 12.1 Hz, 6H), 0.64 (dd, *J* = 6.8, 15.4 Hz, 6H);

^{13}C NMR ($CDCl_3$, 176 MHz) δ 173.4, 138.9, 137.4, 132.2, 128.5, 128.4, 127.3, 127.2, 127.1, 126.1, 83.0, 52.3, 36.4, 29.4, 19.4 (2), 17.4, 16.7;

HRMS (EI): calcd for $C_{24}H_{30}O_2$ $[M]^+$ 350.2246, found 350.2239.

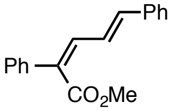

S1 Prepared according to the General Procedure 1 from the α-aryl diazo ester (61.7 mg, 0.35 mmol), *tert*-butyl allylic carbonate (124 mg, 0.50 mmol) and **1** (37 mg, 0.035 mmol). Isolated in 85% yield, *Z,E*/*E,E* = 65:35, after purification by flash chromatography (pentane/Et_2O gradient) as a clear, pale yellow oil.

^{1}H NMR ($CDCl_3$, 500 MHz) δ 7.64 (dd, *J* = 15.6, 11.3 Hz, 1H), 7.53 – 7.48 (m, 2H), 7.41 – 7.34 (m, 6H), 7.34 – 7.2 (m, 2H), 6.89 – 6.84 (m, 2H), 3.88 (s, 3H);

^{13}C NMR ($CDCl_3$, 126 MHz) δ 168.1, 139.7, 138.8, 138.2, 136.7, 132.6, 128.8, 128.7, 128.4, 127.9, 127.8, 127.3, 125.6, 52.0;

HRMS (EI): calcd for $C_{18}H_{16}O_2$ $[M]^+$ 264.1150, found 264.1148.

VII. Computational Methods

To determine the relative energies of the *E,E* and *Z,E* isomers, the ground state geometries were optimized using density functional theory (DFT) with the hybrid PBE0 functional[S6] and the cc-pVDZ basis set[S7] for all atoms. The *E,E* and *Z,E* isomers were considered for both methyl and t-butyl substituents; two possible rotamers were also examined, see Figures S1 and S2. Solvation, which was shown to play an important role, see Table 1, was accounted for in the geometry optimizations by using the integral equation formalism polarizable continuum model (IEF-PCM[S8] and universal force field (UFF) atomic radii) with parameters for N,N-dimethylacetimide (DMA); IEF-PCM will simply be designated as PCM hereafter. To confirm the structures as minima, harmonic vibrational frequencies were computed at the same level of theory. These harmonic frequencies were also used to determine the relative Gibbs free energies at 298.15 K. The DFT computations were all undertaken using Gaussian 09 (G09).[S9] The ground state geometries were optimized using the "Tight" convergence criteria as defined in G09, i.e., maximum force = 1.5×10^{-5} a.u., root mean squared (RMS) force = 1.0×10^{-5} a.u., maximum displacement = 6.0×10^{-5}, and RMS displacement = 4.0×10^{-5}. The numerical integration grid used was set to "Ultrafine" as defined in G09, i.e., a pruned grid of 99 radial shells and 590 angular points per shell.

S6 (a) C. Adamo, V. Barone, *J. Chem. Phys.* **1999**, *110*, 6158–6169; (b) J. P. Perdew, K. Burke, M. Ernzerhoff, *Phys. Rev. Lett*. **1996** *77*, 3865–3868; (Erratum) *Phys. Rev. Lett*. **1996**, *77*, 1396.

S7 T. H. Dunning, Jr. *J. Chem. Phys.* **1989**, *90*, 1007.

S8 (a) E. Cancès, B. Mennucci, J. Tomasi, *J. Chem. Phys.* **1997**, *107*, 3032–3041; (b) M. Cossi, G. Scalmani, N. Rega, V. Barone, *J. Chem. Phys.*, **2002**, *117*, 43–54.

S9 Frisch, M. J. et. Al. Gaussian 09, Revision E.01, Gaussian Inc. Wallingford CT, 2009.

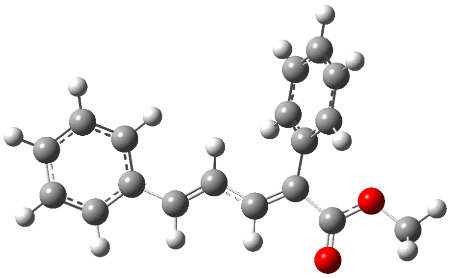

E,E-methyl ester (**18**)
Relative ΔG = 0 at 298.15 K
Relative population = 1

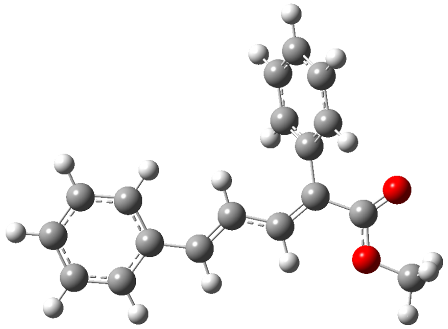

E,E-methyl (rotamer) (**18**)
Relative ΔG = 2.1 kJ/mol at 298.15 K
Relative population = 0.421

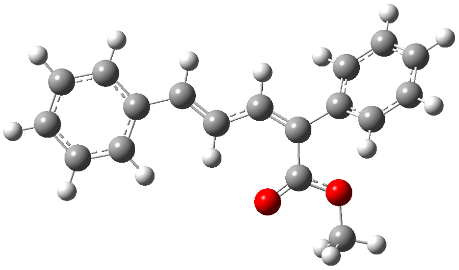

Z,E-methyl ester (**18**)
Relative ΔG = 6.5 kJ/mol at 298.15 K
Relative population = 0.074

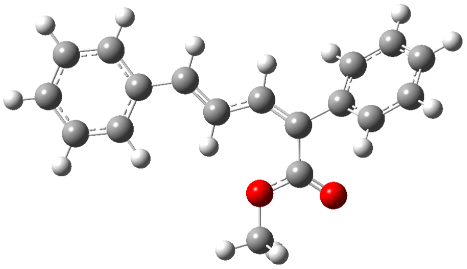

Z,E-methyl ester (rotamer) (**18**)
Relative ΔG = 10.1 kJ/mol at 298.15 K
Relative population = 0.017

Figure S1: Relative energies and populations of *E,E* and *Z,E* isomers. All results computed at PBE0/cc-pVDZ level of theory in PCM(N,N-dimethylacetimide).

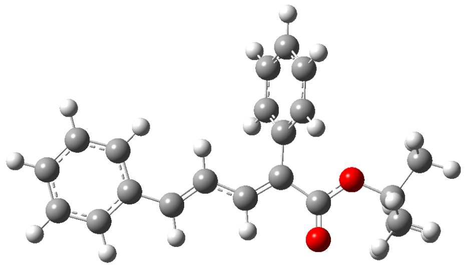

E,E-t-Bu ester (**2**)
Relative ΔG = 0 at 298.15 K
Relative population = 1

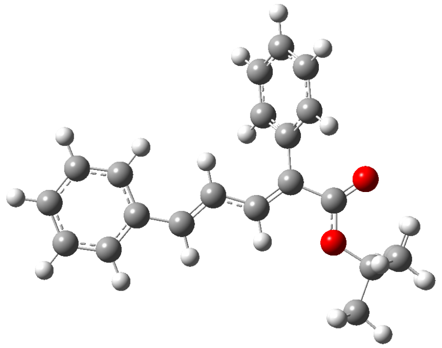

E,E-t-Bu ester (rotomer) (**2**)
Relative ΔG = 1.0 kJ/mol at 298.15 K
Relative population = 0.715

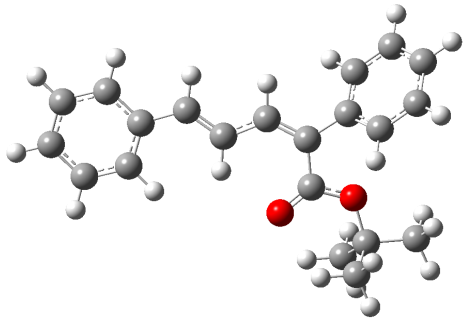

Z,E-t-Bu ester (**2**)
Relative ΔG = 6.7 kJ/mol at 298.15 K
Relative population = 0.067

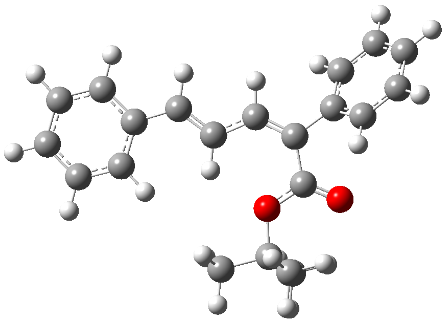

E,E-t-Bu ester (rotomer) (**2**)
Relative ΔG = 10.1 kJ/mol at 298.15 K
Relative population = 0.017

Figure S2: Relative energies and populations of *E,E* and *Z,E* isomers. All results computed at PBE0/cc-pVDZ level of theory in PCM(N,N-dimethylacetimide).

Table S1: Cartesian coordinates (in Å) for the *E,E*-methyl isomer as determined at the PBE0/cc-pVDZ level of theory in PCM(N,N-dimethylacetimide)

C,-6.8473774154,3.6532840702,1.3896283465
C,-5.4566309418,3.8580787456,1.4312550322
C,-4.9829615371,5.1756249151,1.5812205652
C,-5.8695962571,6.2404797799,1.6849611785
C,-7.249254029,6.0195578818,1.6417445442
C,-7.7349040812,4.720860182,1.4935141664
H,-7.2312106718,2.6366753782,1.2734361482
H,-3.9096826806,5.3709016471,1.6171828559
H,-5.4832644639,7.2551579872,1.8008964367
H,-7.9416437627,6.8597633682,1.7236780591
H,-8.8107793338,4.5380277156,1.458586263

C,-4.5758094261,2.7012566772,1.3174879992
H,-5.0845866853,1.7368942461,1.205833948
C,-3.2207552875,2.6983680041,1.332703092
H,-2.6649258399,3.6336073323,1.437170846
C,-2.4688879948,1.4779982435,1.2273282298
H,-3.0381192409,0.5436819366,1.1880630873
C,-1.114919513,1.3511324888,1.2011850153
C,-0.1795253796,2.5020352131,1.2027597242
C,0.7895480233,2.6499277349,2.206996496
C,-0.2609600025,3.4824696611,0.2030601672
C,1.6407248678,3.7516820807,2.2169753876
H,0.8707075332,1.8946377166,2.9907217994
C,0.5952533657,4.5833155841,0.2100343865
H,-0.9966938526,3.3709929005,-0.5965657161
C,1.5474122426,4.7229309348,1.2184273049
H,2.3832344563,3.8534869605,3.0114060942
H,0.5186258714,5.3329140117,-0.5804769786
H,2.2182060292,5.5845540403,1.225912215
C,-0.5989402451,-0.0426195754,1.149687442
O,-1.2914808159,-1.0424715198,1.1660958118
O,0.737411606,-0.0888465598,1.0681397133
C,1.3030628185,-1.3979636175,1.0148772163
H,0.9472342485,-1.93851872,0.1263594505
H,2.3872781704,-1.2512620309,0.9628516094
H,1.0372198548,-1.9735731045,1.9127605526

Table S2: Cartesian coordinates (in Å) for the *E,E*-methyl isomer (rotamer) as determined at the PBE0/cc-pVDZ level of theory in PCM(N,N-dimethylacetimide)

C,-4.5710274288,-1.4084265973,-0.0770455227
C,-3.437839491,-0.5803721656,0.0133050543
C,-3.6375481917,0.8058229176,0.1595744276
C,-4.920988803,1.3356891999,0.212866285
C,-6.0367810101,0.4985384731,0.1216391204
C,-5.8565056815,-0.8765364642,-0.0237393795
H,-4.4332308393,-2.4865682111,-0.1910445965
H,-2.7805796654,1.4778787061,0.2326228876
H,-5.0557263125,2.4132467101,0.3268292791
H,-7.0430970016,0.9199644235,0.1640634249
H,-6.7215873279,-1.5388110066,-0.0959068386
C,-2.1164467775,-1.1944608789,-0.0480055849
H,-2.1141061461,-2.2845274864,-0.1626846425
C,-0.9171372968,-0.566838775,0.0177845869
H,-0.8655117478,0.5191413311,0.1274128859
C,0.3194628376,-1.2997275641,-0.0332320502
H,0.2345418124,-2.3890042181,-0.0636122693
C,1.5771004556,-0.7780249857,-0.0101920493
C,1.8669189803,0.6754169217,-0.0271857342
C,2.6817118828,1.2641871087,0.9523056831
C,1.3289244167,1.4958535114,-1.0299764339
C,2.9307480016,2.6341212866,0.9398504018
H,3.1179340577,0.6403154395,1.733490364
C,1.5828150902,2.8668002944,-1.0453266524
H,0.7174843184,1.0485305336,-1.8168146773
C,2.3821472795,3.4413257474,-0.0582093838
H,3.559492082,3.0756713514,1.7160992597
H,1.1576529375,3.4866050534,-1.8376107082
H,2.5820538498,4.5148316226,-0.0683107472
C,2.7501045322,-1.6884135173,0.0276009906
O,3.9064789752,-1.3163614346,0.0710687901
O,2.411948614,-2.9878498763,0.0074944094
C,3.5051344739,-3.9036556453,0.0332190044
H,4.1003560102,-3.7736956721,0.9482346538
H,3.0563157255,-4.9026043155,0.0095922393
H,4.1570791478,-3.7570872978,-0.8396771473

Table S3: Cartesian coordinates (in Å) for the *Z,E*-methyl isomer as determined at the PBE0/cc-pVDZ level of theory in PCM(N,N-dimethylacetimide)

C,-6.7675251183,3.5912091444,1.0697993511
C,-5.3910395509,3.8601797489,1.1748981213
C,-4.9832385995,5.2028911374,1.2923750457
C,-5.9189434915,6.2300029282,1.3029005596
C,-7.2835185151,5.9454345036,1.1968165079
C,-7.7043000545,4.6210672711,1.0803647108
H,-7.1004673634,2.5542807783,0.9781592065
H,-3.9227019052,5.4460208262,1.3773305807
H,-5.5834285015,7.2650395662,1.394980698
H,-8.0147892664,6.7561582073,1.2056836819
H,-8.7679203,4.3886520033,0.997221895
C,-4.4554917151,2.7417183451,1.1573554049
H,-4.9172211131,1.7513561382,1.0663166833
C,-3.1035970851,2.806577158,1.2397423645
H,-2.5820592655,3.7600381314,1.3199442138
C,-2.3089460899,1.6076690128,1.2124277849
H,-2.8819601265,0.6772779528,1.127153398
C,-0.9528289117,1.465190088,1.2768605576
C,-0.3678007698,0.1006213812,1.2727286782
C,0.702832738,-0.2382974431,0.4287653531
C,-0.917277779,-0.9056202566,2.0822708207
C,1.1949235391,-1.5396889226,0.388495613
H,1.1471760269,0.5250348756,-0.2116961478
C,-0.4213683148,-2.2080133195,2.0454516158
H,-1.7334122064,-0.6576153168,2.7646346632
C,0.6360644803,-2.5313246955,1.1968122536
H,2.0213000165,-1.7827740378,-0.2829939916
H,-0.8607772202,-2.9715344899,2.6909734056
H,1.0269652783,-3.550477504,1.1671688489
C,-0.0622518856,2.6505764602,1.3815427381
O,-0.3931478444,3.8108691571,1.2229388535
O,1.1938462347,2.3076381474,1.7038497934
C,2.1114029705,3.3931553359,1.8307565705
H,1.7939090477,4.078503531,2.6293382637
H,3.075984912,2.9379742865,2.0799444922
H,2.1841046895,3.9537973197,0.8881568704

Table S4: Cartesian coordinates (in Å) for the *Z,E*-methyl isomer (rotamer) as determined at the PBE0/cc-pVDZ level of theory in PCM(N,N-dimethylacetimide)

C,-3.1208030068,0.2371527638,-0.0012484557
C,-4.0375186293,1.3015196416,-0.071797442
C,-3.5289977569,2.6099809823,-0.1831321
C,-2.1588452728,2.837653638,-0.2217602645
C,-1.2612584394,1.7682922802,-0.1505707627
C,-1.7481525876,0.4662751014,-0.040059343
H,-3.4989890254,-0.7845171703,0.0855248309
H,-4.2123939019,3.4590238896,-0.2401753228
H,-1.7835958466,3.8594267488,-0.3082458521
H,-0.1855441134,1.9525810311,-0.1812948594
H,-1.055324856,-0.3757428642,0.0162764765
C,-5.4636941112,1.0006968518,-0.0264158845
H,-5.7113974185,-0.0634052811,0.0644627256
C,-6.4880995401,1.8874873422,-0.0845209562
H,-6.2982745993,2.956272819,-0.1696558751
C,-7.8519457247,1.4311299595,-0.0341680607
H,-7.9533582569,0.3420675364,0.0353112642
C,-9.0303495133,2.1205818005,-0.0695415124
C,-10.3076443215,1.3644367195,-0.0807644483
C,-11.4011621614,1.7613447682,0.7071240398
C,-10.4397739829,0.2019800421,-0.8568546797
C,-12.57323183,1.0107216478,0.7312619631
H,-11.3280846647,2.6633193205,1.3155809479
C,-11.614641792,-0.547425717,-0.8356401042
H,-9.6160173307,-0.1080679396,-1.503707755
C,-12.6867428885,-0.1476276846,-0.0392477628
H,-13.4061262054,1.3329820749,1.3600474013
H,-11.694236454,-1.4437812536,-1.45446101
H,-13.6091293471,-0.7318551571,-0.0228609067
C,-9.1554799396,3.6008430243,-0.1421838227
O,-10.1875225548,4.1797524177,-0.4187502306
O,-8.0198884791,4.260957125,0.1308853784
C,-8.103947445,5.6841486538,0.039812824
H,-8.8422399414,6.0741708516,0.7542110182
H,-7.1028987553,6.0554661974,0.2845184501
H,-8.3902432665,5.9932502784,-0.9752043889

Table S5: Cartesian coordinates (in Å) for the *E,E*-t-butyl isomer as determined at the PBE0/cc-pVDZ level of theory in PCM(N,N-dimethylacetimide)

C,-6.8422481176,3.7541574265,1.4681652611
C,-5.4454032246,3.9165181538,1.4556614714
C,-4.9283111803,5.225573409,1.4958304174
C,-5.7790543999,6.3230967698,1.546992907
C,-7.1653882092,6.1441523348,1.5592010617
C,-7.6938584227,4.8543410942,1.5197077918
H,-7.2595239646,2.74455762,1.4366581651
H,-3.8490849272,5.3884320714,1.4854071313
H,-5.3590003928,7.3305553291,1.5771756094
H,-7.829555419,7.0098155394,1.5991724956
H,-8.7752954346,4.7039328401,1.5287196207
C,-4.6036123598,2.7265741312,1.3999367072
H,-5.145101665,1.7744446174,1.3600127165
C,-3.2494858851,2.6804100412,1.3907678809
H,-2.662810551,3.6019458698,1.427636388
C,-2.5368204742,1.4324180421,1.3455342037
H,-3.134002231,0.5151589651,1.3671241991
C,-1.1884747566,1.2633589468,1.3068026842
C,-0.2238275409,2.3879281722,1.2342014948
C,0.7396568096,2.5835002815,2.2355099697
C,-0.2718959113,3.2960141821,0.1667149288
C,1.6194182139,3.6610160492,2.1754304067
H,0.7929705178,1.8853346746,3.0729057416
C,0.6133009027,4.3721930742,0.1031545553
H,-1.0052892294,3.1472319457,-0.6290535847
C,1.5605391441,4.5595350787,1.1083347072
H,2.3572137833,3.8012004095,2.9684544892
H,0.562463019,5.0651334647,-0.7394603889
H,2.2536710022,5.4020061366,1.060774492
C,-0.7092798241,-0.150483487,1.3204630229
O,-1.4505834701,-1.1129921278,1.4074757541
O,0.6196310248,-0.217963296,1.2110137009
C,1.3302146469,-1.503068173,1.2010418433
C,1.1039941622,-2.2370675634,2.5160903713
C,0.9022929109,-2.3264129452,-0.006797497
C,2.7840078277,-1.0743465873,1.0689933292
H,1.379473394,-1.5939876416,3.3656574389
H,0.0577151902,-2.5464402292,2.6266072621
H,1.7427096571,-3.1325617521,2.5450212904
H,1.0298960911,-1.7431362381,-0.9312364798
H,1.5375131564,-3.2220496899,-0.0777601797
H,-0.1447845766,-2.641857316,0.0747858744

H,3.4357491714,-1.9595690752,1.0456565173
H,2.9367269258,-0.5036763278,0.1412527107
H,3.0818348463,-0.4440596108,1.9198107869

Agilent Technologies

Department of Chemistry, University of Alberta

Recorded on: **u500, Aug 5 2016** Sweep Width(Hz): **6009.62** Acquisiton Time(s): **5** Relaxation Delay(s): **0.1**
Pulse Sequence: **PRESAT** Digital Res.(Hz/pt): **0.09** Hz per mm(Hz/mm): **17.57** Completed Scans **16**

Bryce, BT-07-203-B-F15-19
499.797 MHz H1 PRESAT in cdcl3 (ref. to CDCl3 @ 7.26 ppm), temp 27.7 C -> actual temp = 27.0 C, colddual probe

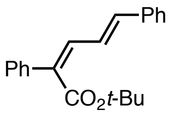

2

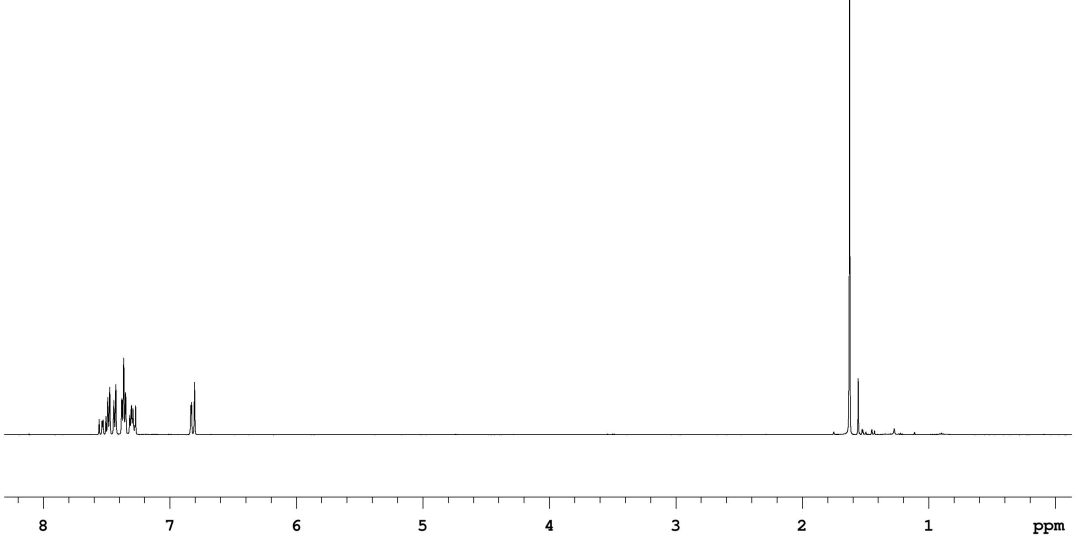

Agilent Technologies

Department of Chemistry, University of Alberta

Recorded on: **u500, Aug 5 2016** Sweep Width(Hz): **33783.8** Acquisiton Time(s): **1** Relaxation Delay(s): **1**
Pulse Sequence: **s2pul** Digital Res.(Hz/pt): **0.26** Hz per mm(Hz/mm): **109.37** Completed Scans **128**

Bryce, BT-07-203-B-F15-19
125.688 MHz C13[H1] 1D in cdcl3 (ref. to CDCl3 @ 77.06 ppm), temp 27.7 C -> actual temp = 27.0 C, colddual probe

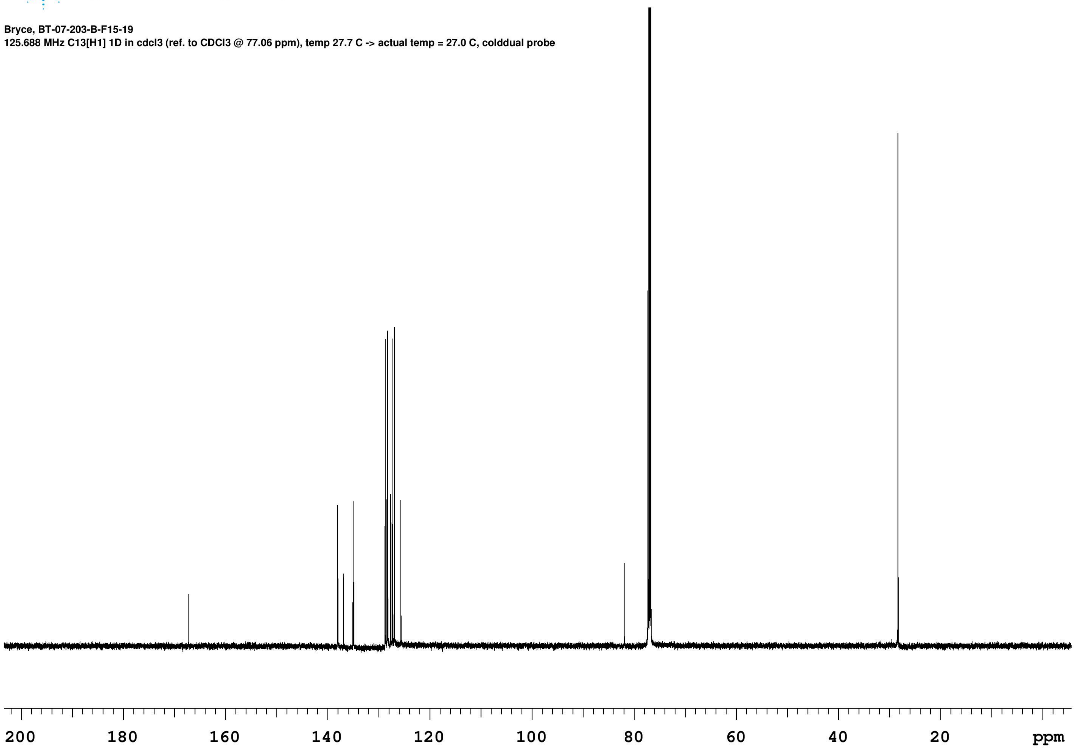

Department of Chemistry, University of Alberta

Agilent Technologies

Recorded on: ibd5, Jan 17 2017	Sweep Width(Hz): 6000.6	Acquisiton Time(s): 5	Relaxation Delay(s): 0.1
Pulse Sequence: s2pul	Digital Res.(Hz/pt): 0.09	Hz per mm(Hz/mm): 16.9	Completed Scans 16

RL-06-71-B
498.118 MHz H1 1D in cdcl3 (ref. to CDCl3 @ 7.26 ppm)
temp 26.9 C -> actual temp = 27.0 C, autoxdb probe

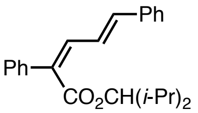

3

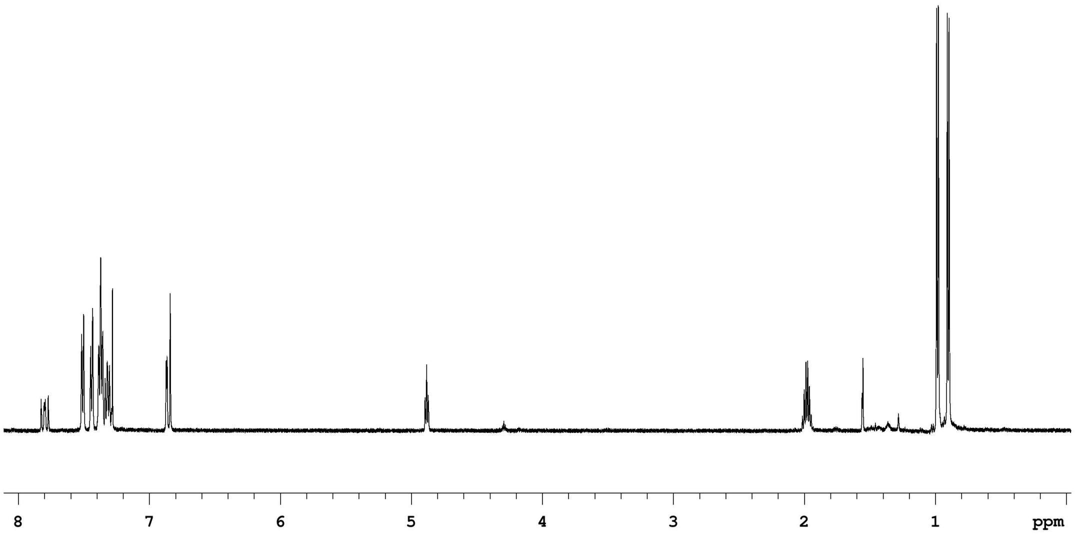

Department of Chemistry, University of Alberta

Agilent Technologies

Recorded on: v700, Jan 17 2017	Sweep Width(Hz): 48076.9	Acquisiton Time(s): 1	Relaxation Delay(s): 1
Pulse Sequence: s2pul	Digital Res.(Hz/pt): 0.37	Hz per mm(Hz/mm): 153.55	Completed Scans 512

Rylan, RL-06-71-B
175.975 MHz C13{H1} 1D in cdcl3 (ref. to CDCl3 @ 77.06 ppm)
temp 27.5 C -> actual temp = 27.0 C, coldid probe

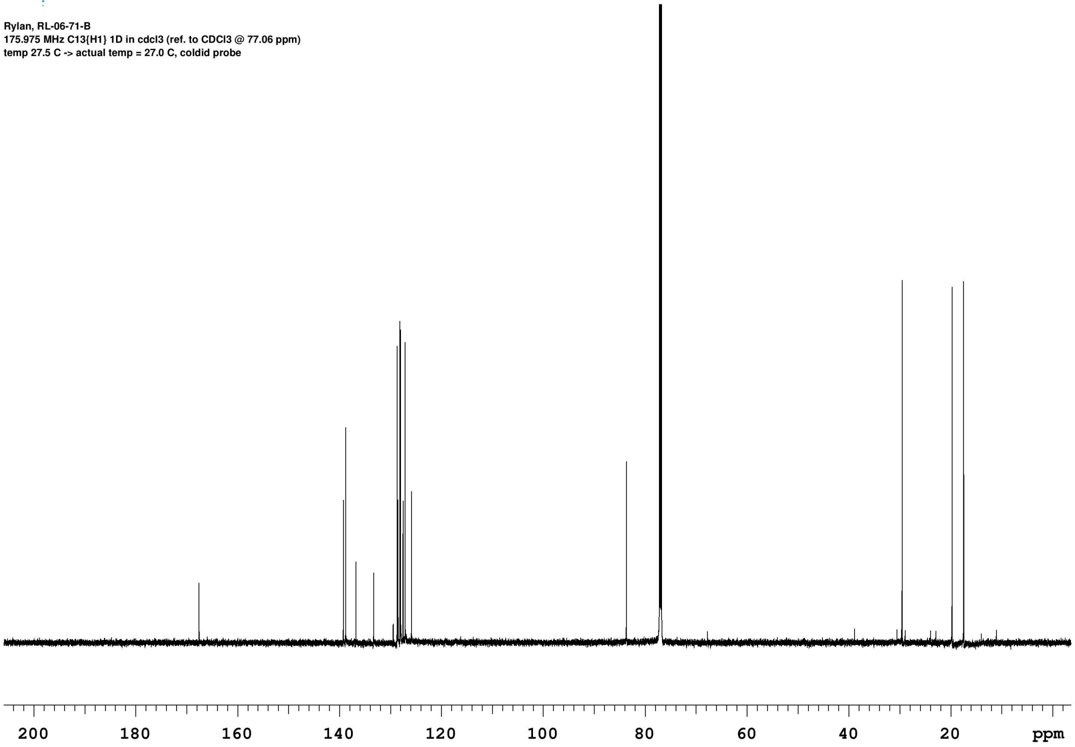

Department of Chemistry, University of Alberta

Agilent Technologies

Recorded on: **m400, Jul 24 2016**	Sweep Width(Hz): **4803.07**	Acquisiton Time(s): **4.997**	Relaxation Delay(s): **0.1**
Pulse Sequence: **PRESAT**	Digital Res.(Hz/pt): **0.07**	Hz per mm(Hz/mm): **14.1**	Completed Scans **32**

Bryce, BT-07-165-A
400.389 MHz H1 PRESAT in cdcl3 (ref. to CDCl3 @ 7.26 ppm), temp 27.0 C -> actual temp = 27.0 C, m400gz probe

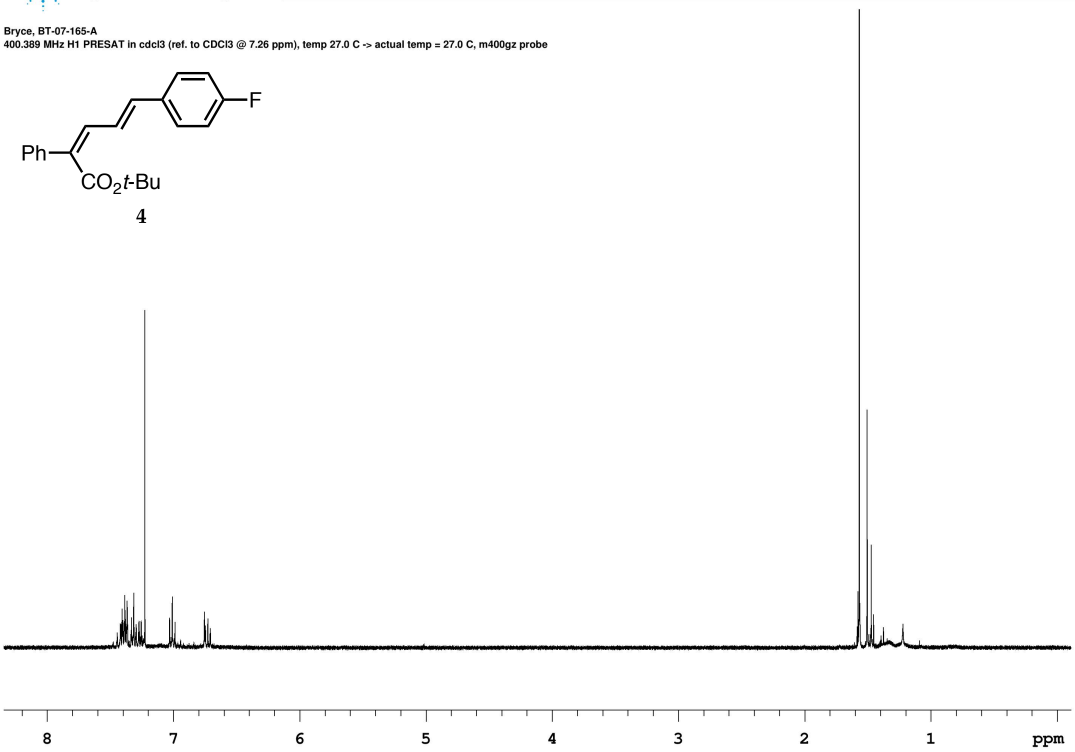

Department of Chemistry, University of Alberta

Agilent Technologies

Recorded on: **u500, Jul 24 2016**	Sweep Width(Hz): **33783.8**	Acquisiton Time(s): **1**	Relaxation Delay(s): **1**
Pulse Sequence: **s2pul**	Digital Res.(Hz/pt): **0.26**	Hz per mm(Hz/mm): **109.29**	Completed Scans **256**

Bryce, BT-07-165-A
125.688 MHz C13[H1] 1D in cdcl3 (ref. to CDCl3 @ 77.06 ppm), temp 27.7 C -> actual temp = 27.0 C, colddual probe

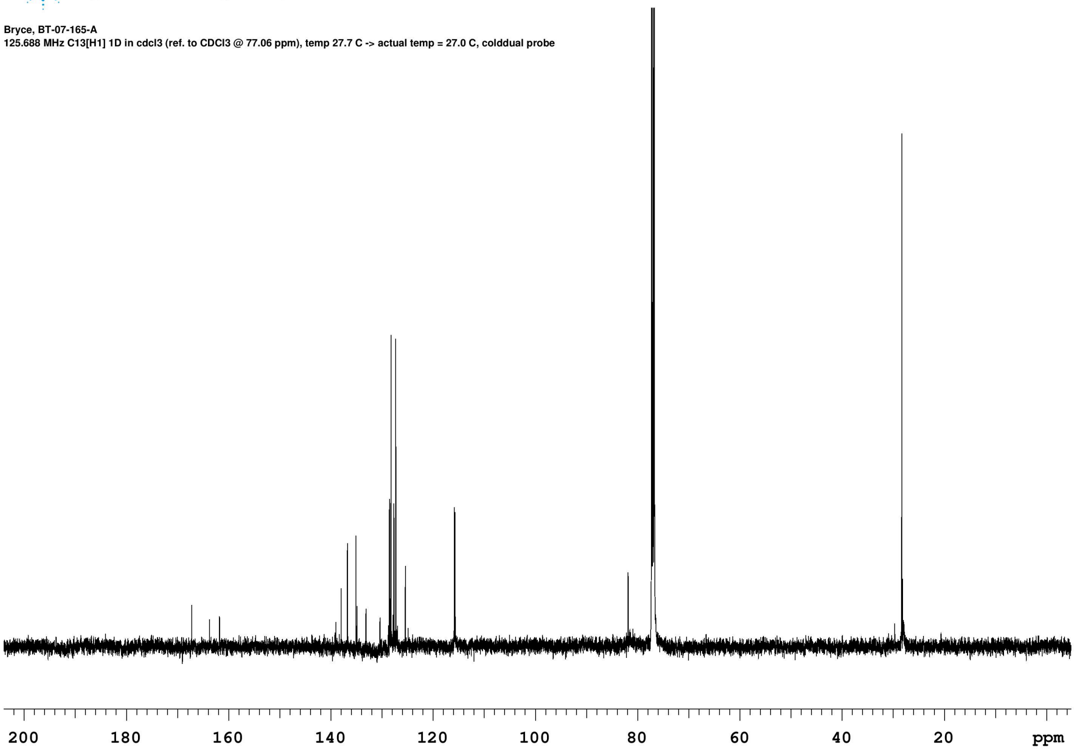

Department of Chemistry, University of Alberta

Recorded on: i600, Feb 4 2017 | Sweep Width(Hz): 7183.91 | Acquisiton Time(s): 5 | Relaxation Delay(s): 0.1
Pulse Sequence: s2pul | Digital Res.(Hz/pt): 0.05 | Hz per mm(Hz/mm): 20.91 | Completed Scans 4

RL-06-87
599.926 MHz H1 1D in cdcl3 (ref. to CDCl3 @ 7.26 ppm)
temp 25.8 C -> actual temp = 27.0 C, autoxid probe

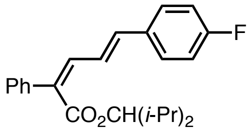

5

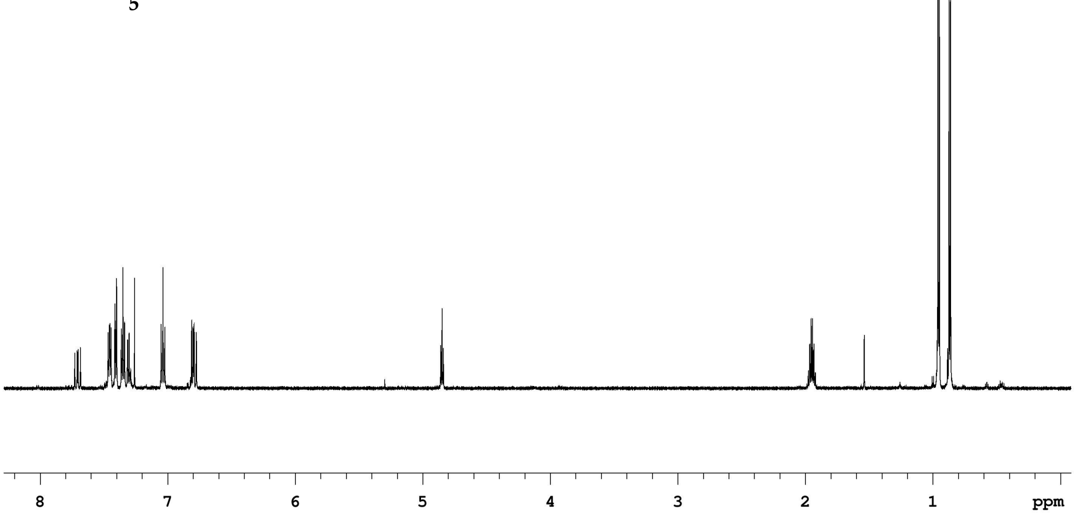

Department of Chemistry, University of Alberta

Recorded on: i600, Feb 4 2017 | Sweep Width(Hz): 40322.6 | Acquisiton Time(s): 1 | Relaxation Delay(s): 1
Pulse Sequence: s2pul | Digital Res.(Hz/pt): 0.31 | Hz per mm(Hz/mm): 128.41 | Completed Scans 1364

RL-06-87
150.868 MHz C13{H1} 1D in cdcl3 (ref. to CDCl3 @ 77.06 ppm)
temp 25.8 C -> actual temp = 27.0 C, autoxid probe

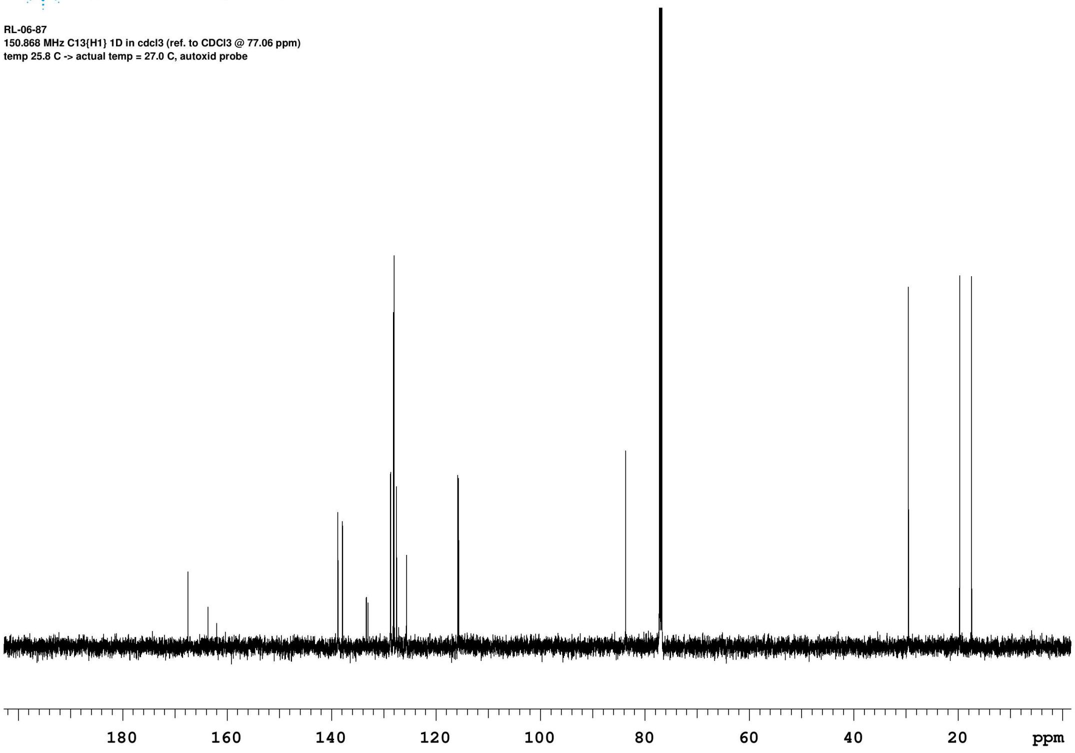

Agilent Technologies

Department of Chemistry, University of Alberta

Recorded on: u500, Aug 10 2016 | Sweep Width(Hz): 6009.62 | Acquisiton Time(s): 5 | Relaxation Delay(s): 0.1
Pulse Sequence: PRESAT | Digital Res.(Hz/pt): 0.09 | Hz per mm(Hz/mm): 17.81 | Completed Scans 16

Bryce, BT-08-023-B
499.797 MHz H1 PRESAT in cdcl3 (ref. to CDCl3 @ 7.26 ppm), temp 27.7 C -> actual temp = 27.0 C, colddual probe

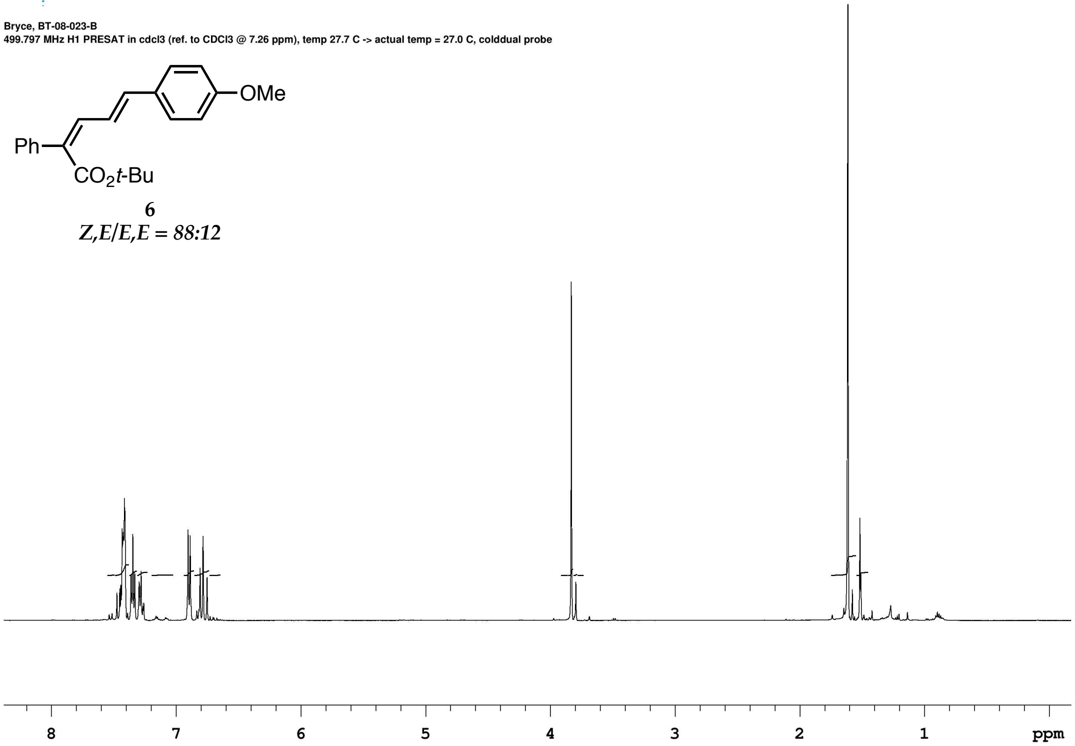

6

Z,E/E,E = 88:12

Agilent Technologies

Department of Chemistry, University of Alberta

Recorded on: u500, Aug 10 2016 | Sweep Width(Hz): 33783.8 | Acquisiton Time(s): 1 | Relaxation Delay(s): 1
Pulse Sequence: s2pul | Digital Res.(Hz/pt): 0.26 | Hz per mm(Hz/mm): 107.74 | Completed Scans 128

Bryce, BT-08-023-B
125.688 MHz C13[H1] 1D in cdcl3 (ref. to CDCl3 @ 77.06 ppm), temp 27.7 C -> actual temp = 27.0 C, colddual probe

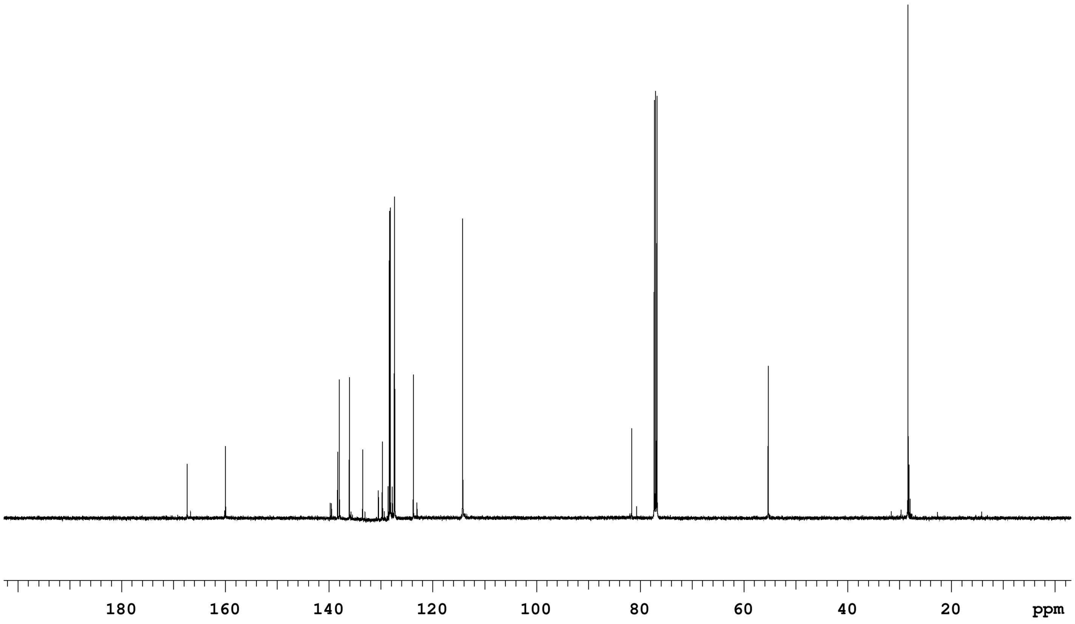

Agilent Technologies

Department of Chemistry, University of Alberta

Recorded on: i600, Feb 16 2017
Pulse Sequence: s2pul
Sweep Width(Hz): 7183.91
Digital Res.(Hz/pt): 0.11
Acquisiton Time(s): 5
Hz per mm(Hz/mm): 21.55
Relaxation Delay(s): 0.1
Completed Scans 16

RL-06-89
599.926 MHz H1 1D in cdcl3 (ref. to CDCl3 @ 7.26 ppm)
temp 25.8 C -> actual temp = 27.0 C, autoxid probe

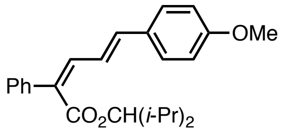

7

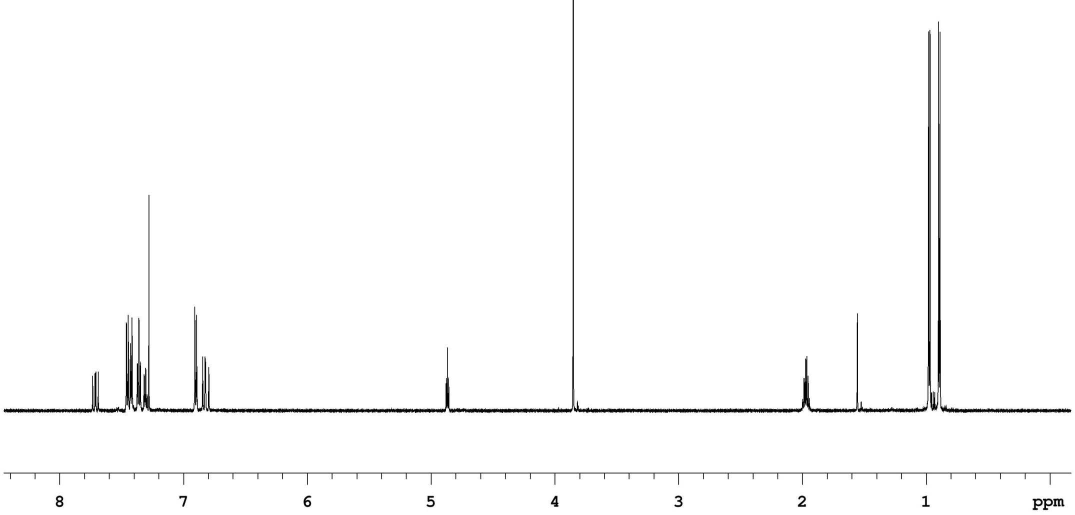

Agilent Technologies

Department of Chemistry, University of Alberta

Recorded on: i600, Feb 16 2017
Pulse Sequence: s2pul
Sweep Width(Hz): 40322.6
Digital Res.(Hz/pt): 0.31
Acquisiton Time(s): 1
Hz per mm(Hz/mm): 144.78
Relaxation Delay(s): 1
Completed Scans 1036

RL-06-89-F1
150.868 MHz C13{H1} 1D in cdcl3 (ref. to CDCl3 @ 77.06 ppm)
temp 25.8 C -> actual temp = 27.0 C, autoxid probe

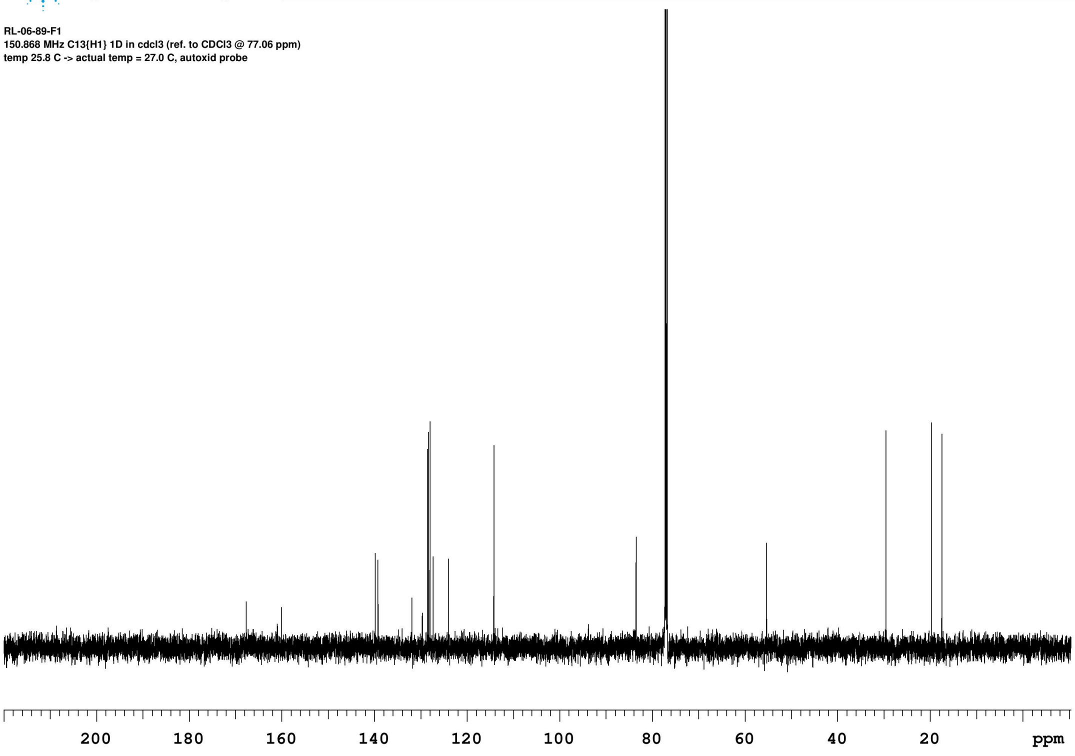

Department of Chemistry, University of Alberta

Agilent Technologies

Recorded on: v700, Oct 5 2016	Sweep Width(Hz): 8389.26	Acquisiton Time(s): 5	Relaxation Delay(s): 0.1
Pulse Sequence: PRESAT	Digital Res.(Hz/pt): 0.13	Hz per mm(Hz/mm): 23.72	Completed Scans 32

Rylan, RL-06-47-P
699.762 MHz H1 PRESAT in cdcl3 (ref. to CDCl3 @ 7.26 ppm), temp 27.5 C -> actual temp = 27.0 C, coldid probe

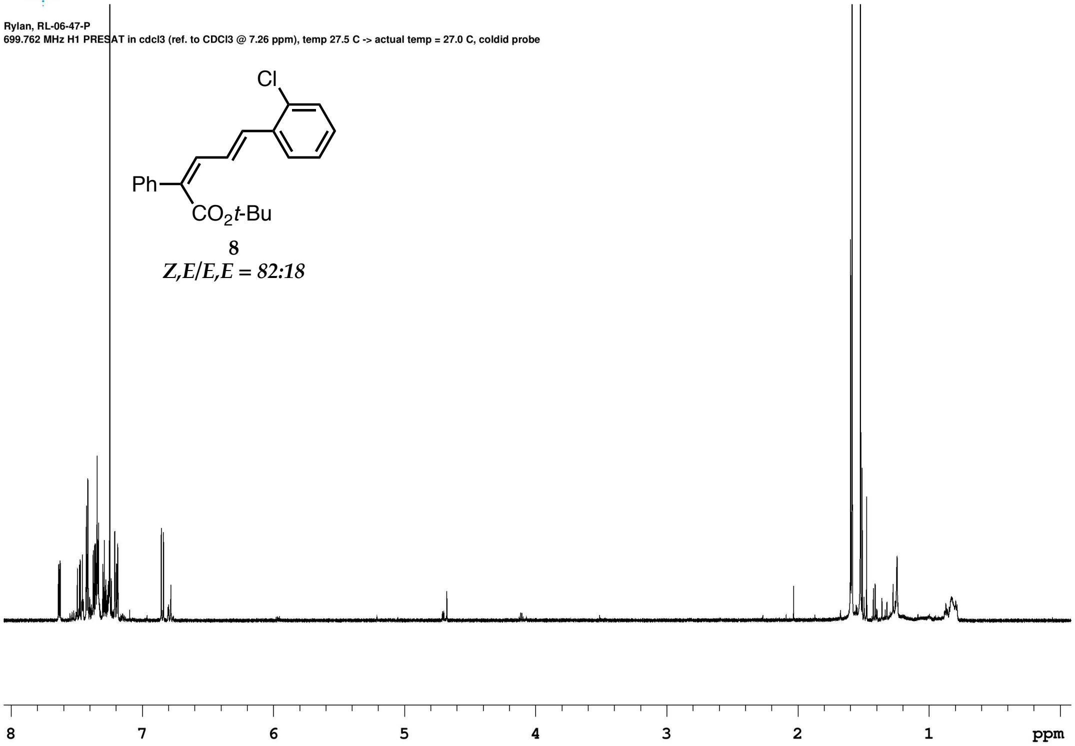

Department of Chemistry, University of Alberta

Agilent Technologies

Recorded on: v700, Jan 7 2017	Sweep Width(Hz): 48076.9	Acquisiton Time(s): 1	Relaxation Delay(s): 1
Pulse Sequence: s2pul	Digital Res.(Hz/pt): 0.37	Hz per mm(Hz/mm): 155.88	Completed Scans 1000

Rylan, RL-06-47-TP
175.975 MHz C13{H1} 1D in cdcl3 (ref. to CDCl3 @ 77.06 ppm)
temp 27.5 C -> actual temp = 27.0 C, coldid probe

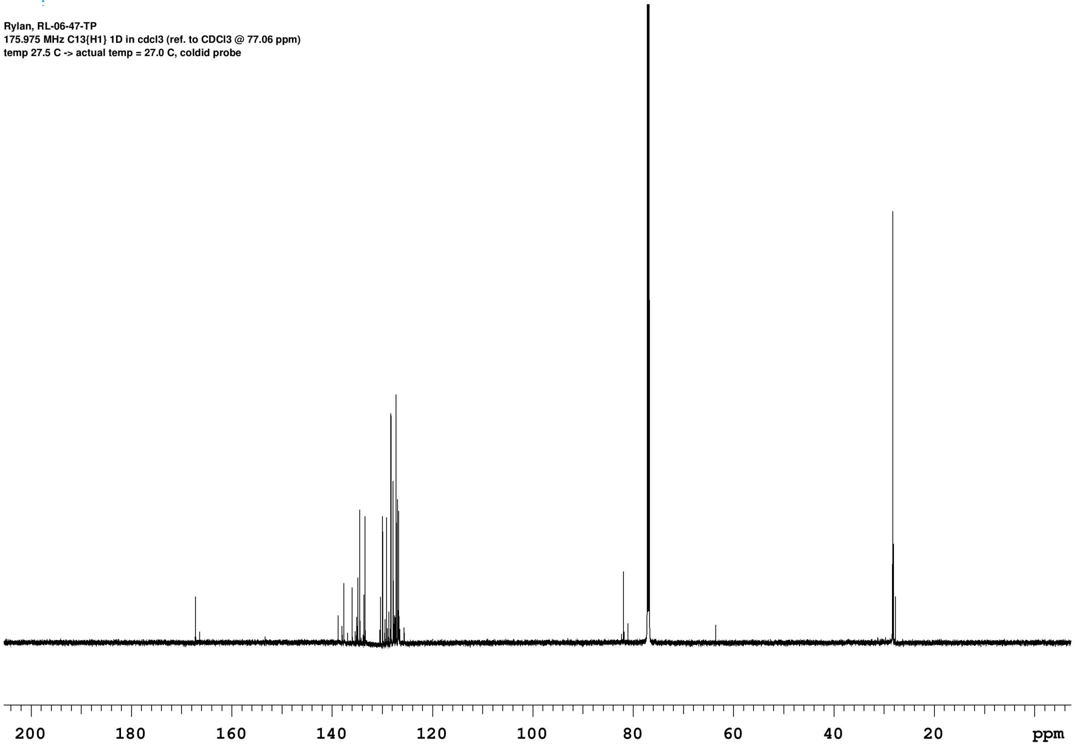

Department of Chemistry, University of Alberta

Agilent Technologies

Recorded on: ibd5, Sep 14 2016	Sweep Width(Hz): 6000.6	Acquisiton Time(s): 5	Relaxation Delay(s): 0.1
Pulse Sequence: s2pul	Digital Res.(Hz/pt): 0.09	Hz per mm(Hz/mm): 18.21	Completed Scans 16

BT-08-031
498.118 MHz H1 1D in cdcl3 (ref. to CDCl3 @ 7.26 ppm), temp 26.4 C -> actual temp = 27.0 C, autoxdb probe

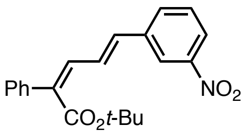

9

Z,E/E,E = 84:16

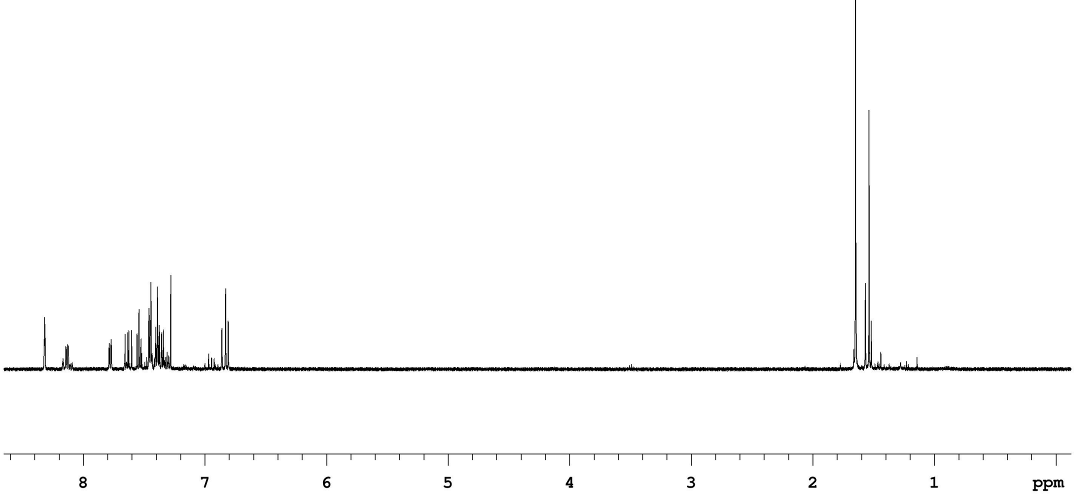

Department of Chemistry, University of Alberta

Agilent Technologies

Recorded on: v700, Jan 7 2017	Sweep Width(Hz): 48076.9	Acquisiton Time(s): 1	Relaxation Delay(s): 1
Pulse Sequence: s2pul	Digital Res.(Hz/pt): 0.37	Hz per mm(Hz/mm): 155.53	Completed Scans 1000

Rylan, BT-08-31
175.975 MHz C13{H1} 1D in cdcl3 (ref. to CDCl3 @ 77.06 ppm)
temp 27.5 C -> actual temp = 27.0 C, coldid probe

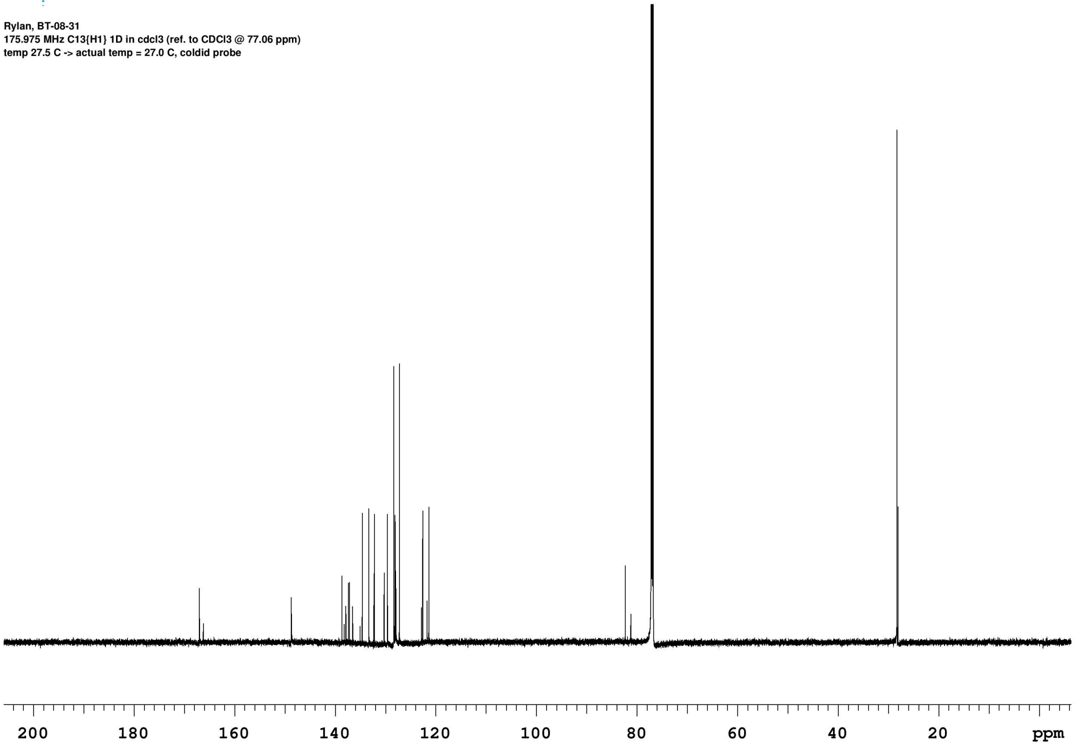

Agilent Technologies

Department of Chemistry, University of Alberta

Recorded on: u500, Oct 19 2016 | Sweep Width(Hz): 6009.62 | Acquisiton Time(s): 5 | Relaxation Delay(s): 0.1
Pulse Sequence: PRESAT | Digital Res.(Hz/pt): 0.09 | Hz per mm(Hz/mm): 17.58 | Completed Scans 16

Bryce, BT-07-167-A-F13-14
499.797 MHz H1 PRESAT in cdcl3 (ref. to CDCl3 @ 7.26 ppm), temp 27.7 C -> actual temp = 27.0 C, colddual probe

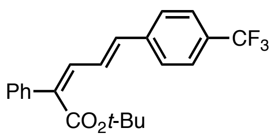

10
Z,E/E,E = 81:19

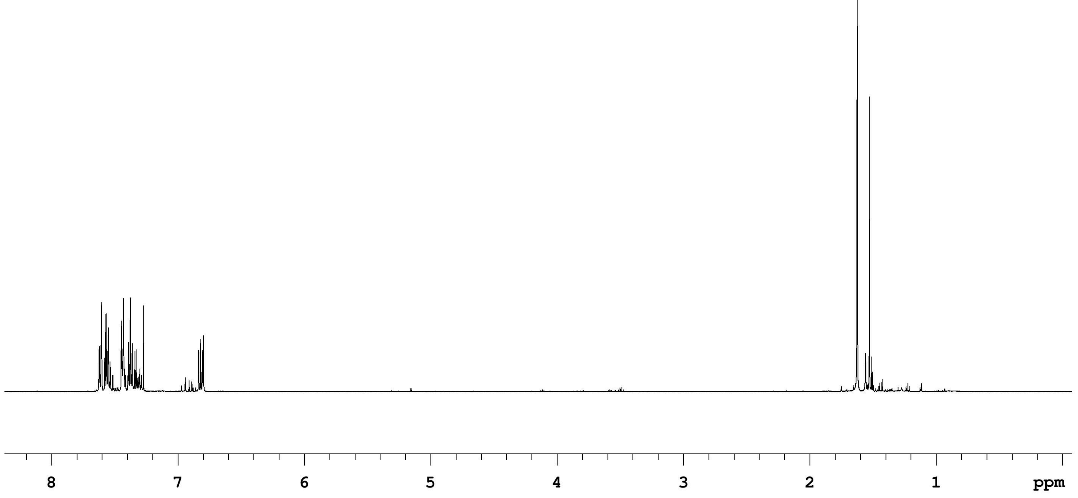

Agilent Technologies

Department of Chemistry, University of Alberta

Recorded on: u500, Aug 30 2016 | Sweep Width(Hz): 33783.8 | Acquisiton Time(s): 1 | Relaxation Delay(s): 1
Pulse Sequence: s2pul | Digital Res.(Hz/pt): 0.26 | Hz per mm(Hz/mm): 106.1 | Completed Scans 128

Bryce, BT-07-167-A-F13-14
125.688 MHz C13[H1] 1D in cdcl3 (ref. to CDCl3 @ 77.06 ppm), temp 27.7 C -> actual temp = 27.0 C, colddual probe

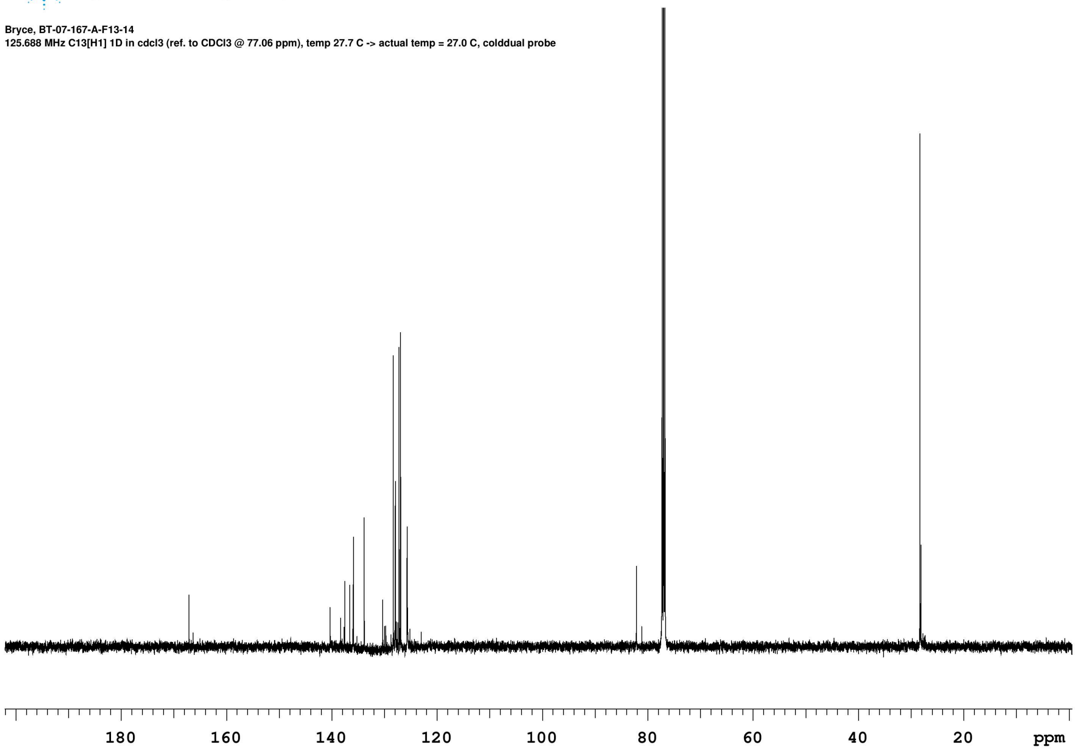

Department of Chemistry, University of Alberta

Agilent Technologies

Recorded on: m400, Jun 17 2016 | Sweep Width(Hz): 4803.07 | Acquisiton Time(s): 4.997 | Relaxation Delay(s): 0.1
Pulse Sequence: PRESAT | Digital Res.(Hz/pt): 0.07 | Hz per mm(Hz/mm): 14.09 | Completed Scans 32

Bryce, BT-07-183-C3
400.389 MHz H1 PRESAT in cdcl3 (ref. to CDCl3 @ 7.26 ppm), temp 27.0 C -> actual temp = 27.0 C, m400gz probe

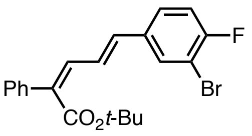

11

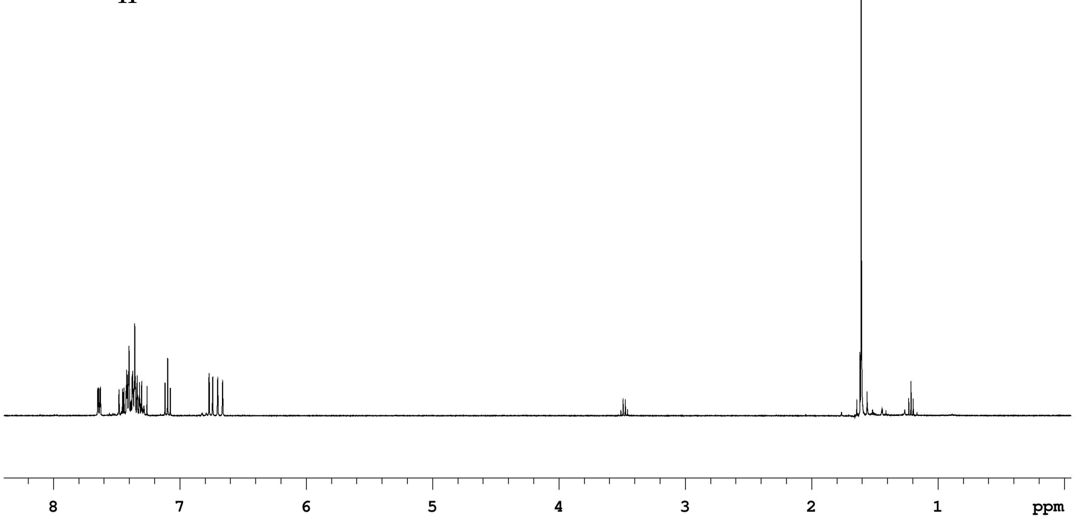

Department of Chemistry, University of Alberta

Agilent Technologies

Recorded on: u500, Jul 24 2016 | Sweep Width(Hz): 31250 | Acquisiton Time(s): 1 | Relaxation Delay(s): 1
Pulse Sequence: s2pul | Digital Res.(Hz/pt): 0.24 | Hz per mm(Hz/mm): 109.11 | Completed Scans 256

Bryce, BT-07-183
125.687 MHz C13[H1] 1D in cdcl3 (ref. to CDCl3 @ 77.06 ppm), temp 27.7 C -> actual temp = 27.0 C, colddual probe

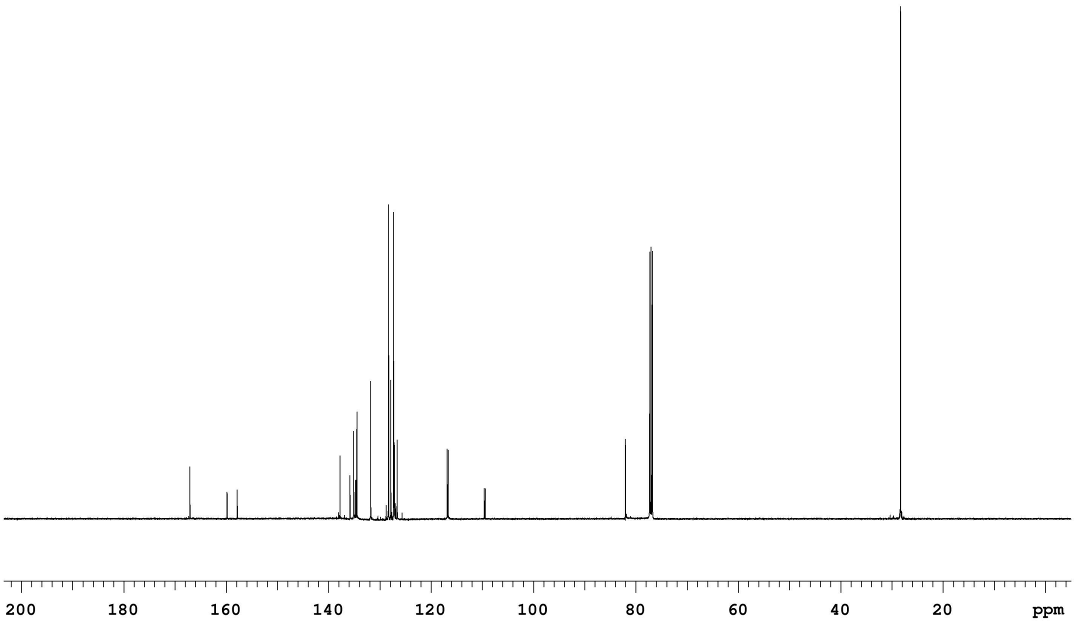

Department of Chemistry, University of Alberta

Agilent Technologies

Recorded on: u500, Sep 15 2016 | Sweep Width(Hz): 6009.62 | Acquisiton Time(s): 5 | Relaxation Delay(s): 0.1
Pulse Sequence: PRESAT | Digital Res.(Hz/pt): 0.09 | Hz per mm(Hz/mm): 17.68 | Completed Scans 16

Bryce, BT-08-055
499.797 MHz H1 PRESAT in cdcl3 (ref. to CDCl3 @ 7.26 ppm), temp 27.7 C -> actual temp = 27.0 C, colddual probe

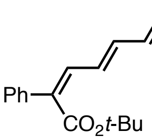

12

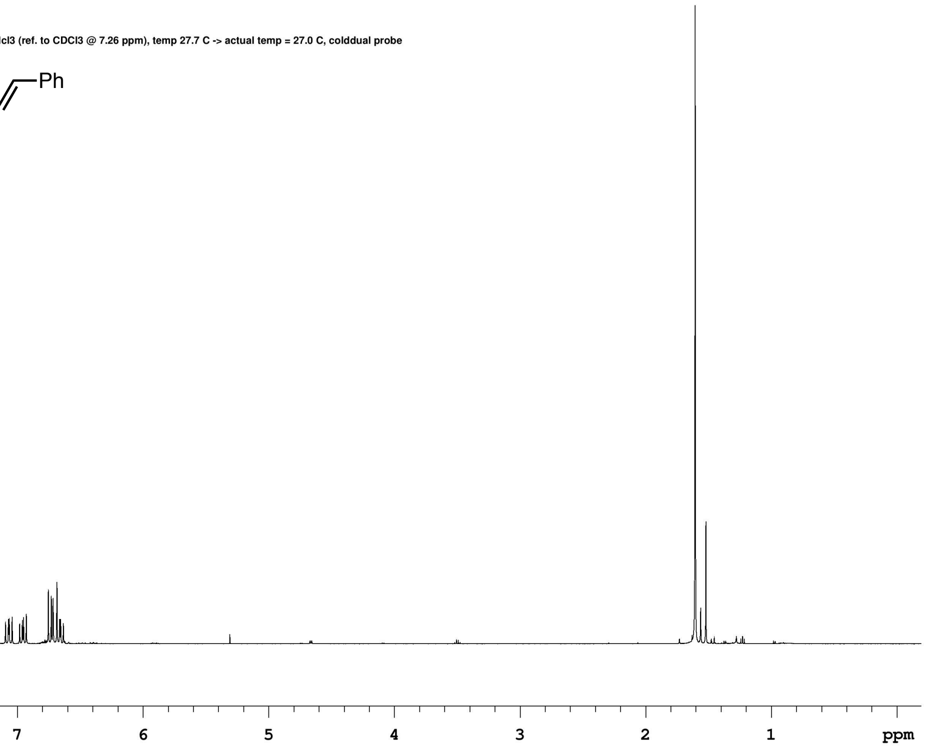

Department of Chemistry, University of Alberta

Agilent Technologies

Recorded on: u500, Sep 15 2016 | Sweep Width(Hz): 33783.8 | Acquisiton Time(s): 1 | Relaxation Delay(s): 1
Pulse Sequence: s2pul | Digital Res.(Hz/pt): 0.26 | Hz per mm(Hz/mm): 108.94 | Completed Scans 112

Bryce, BT-08-055
125.688 MHz C13[H1] 1D in cdcl3 (ref. to CDCl3 @ 77.06 ppm), temp 27.7 C -> actual temp = 27.0 C, colddual probe

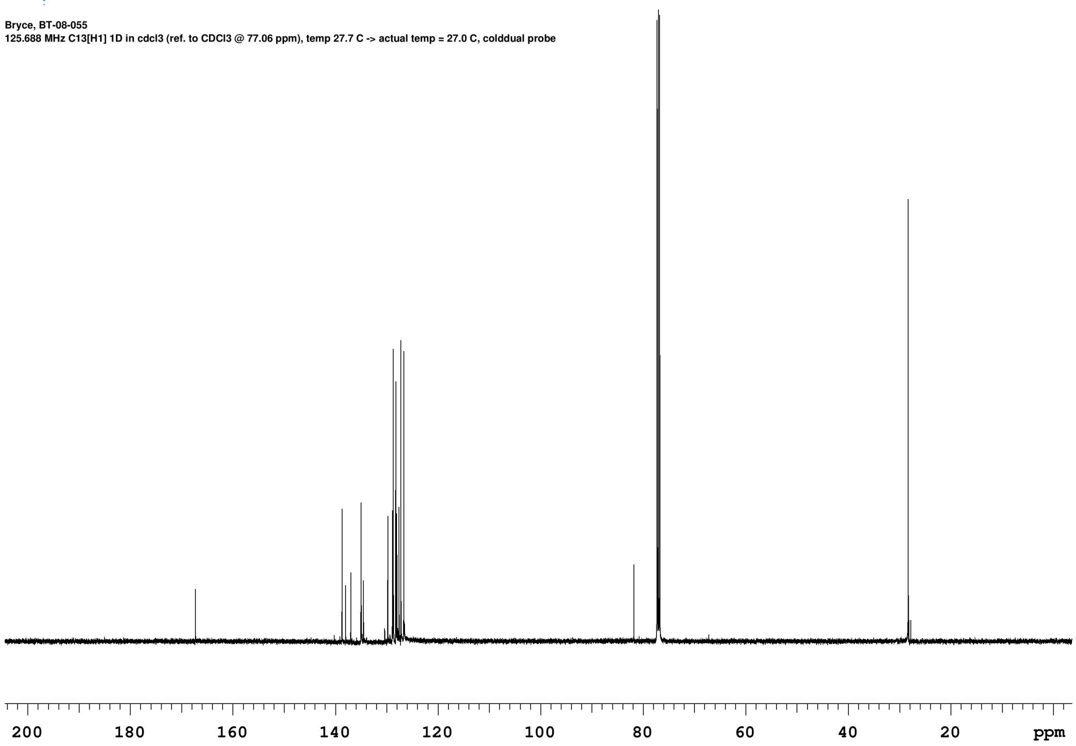

Agilent Technologies

Department of Chemistry, University of Alberta

Recorded on: ibd5, Jan 10 2017 | Sweep Width(Hz): 6000.6 | Acquisiton Time(s): 5 | Relaxation Delay(s): 0.1
Pulse Sequence: s2pul | Digital Res.(Hz/pt): 0.09 | Hz per mm(Hz/mm): 17.48 | Completed Scans 16

RL-06-79
498.118 MHz H1 1D in cdcl3 (ref. to CDCl3 @ 7.26 ppm)
temp 26.9 C -> actual temp = 27.0 C, autoxdb probe

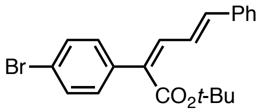

14

Z,E/E,E = 87:13

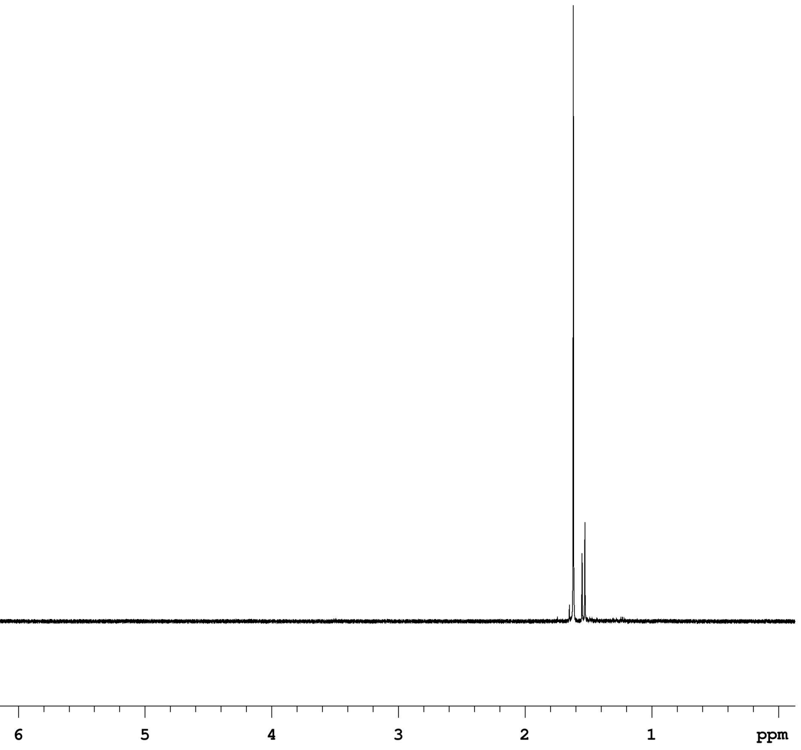

Agilent Technologies

Department of Chemistry, University of Alberta

Recorded on: v700, Jan 10 2017 | Sweep Width(Hz): 48076.9 | Acquisiton Time(s): 1 | Relaxation Delay(s): 1
Pulse Sequence: DEPT_chempack | Digital Res.(Hz/pt): 0.37 | Hz per mm(Hz/mm): 152.04 | Completed Scans 1024

Rylan, RL-06-79
175.974 MHz C13 DEPTq in cdcl3 (ref. to CDCl3 @ 77.06 ppm)
temp 27.5 C -> actual temp = 27.0 C, coldid probe
DEPTq-135, C & CH2 same, CH & CH3 opposite side of solvent signal

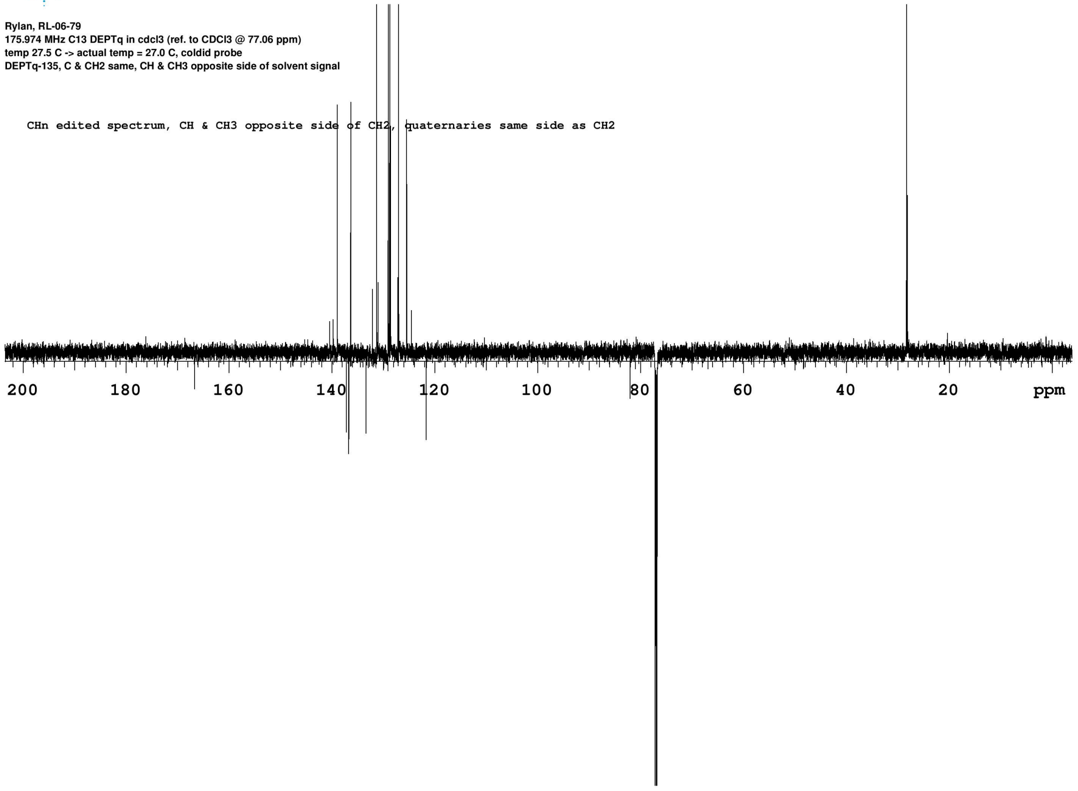

Department of Chemistry, University of Alberta

Recorded on: i600, Sep 23 2016	Sweep Width(Hz): 7183.91	Acquisiton Time(s): 5	Relaxation Delay(s): 0.1
Pulse Sequence: s2pul	Digital Res.(Hz/pt): 0.11	Hz per mm(Hz/mm): 21.1	Completed Scans 8

RL-06-31
599.926 MHz H1 1D in cdcl3 (ref. to CDCl3 @ 7.26 ppm), temp 25.8 C -> actual temp = 27.0 C, autoxid probe

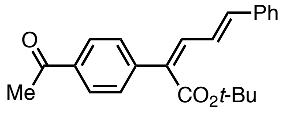

15
Z,E/E,E = 95:5

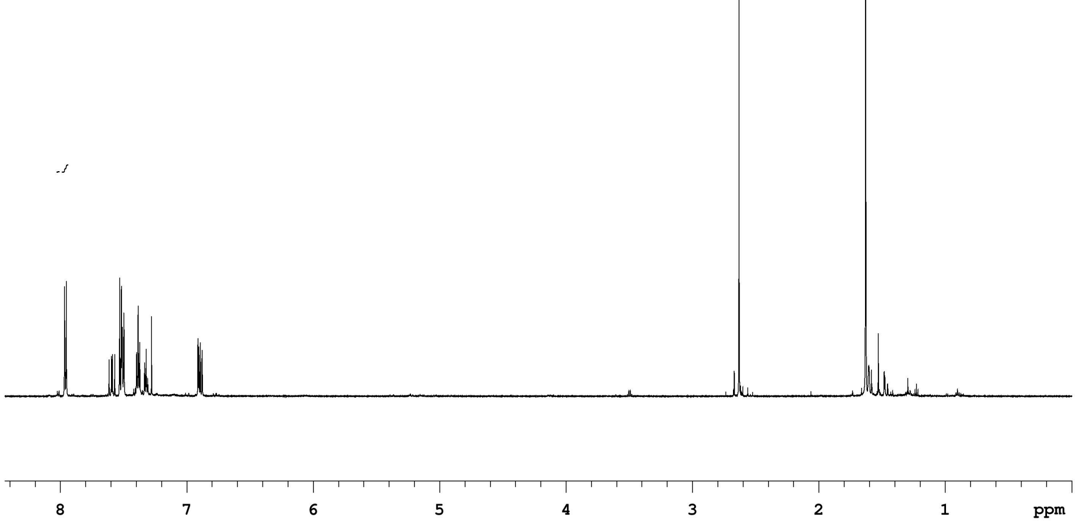

Department of Chemistry, University of Alberta

Recorded on: i600, Sep 23 2016	Sweep Width(Hz): 40322.6	Acquisiton Time(s): 1	Relaxation Delay(s): 1
Pulse Sequence: s2pul	Digital Res.(Hz/pt): 0.31	Hz per mm(Hz/mm): 135.36	Completed Scans 924

RL-06-31
150.868 MHz C13[H1] 1D in cdcl3 (ref. to CDCl3 @ 77.06 ppm), temp 25.8 C -> actual temp = 27.0 C, autoxid probe

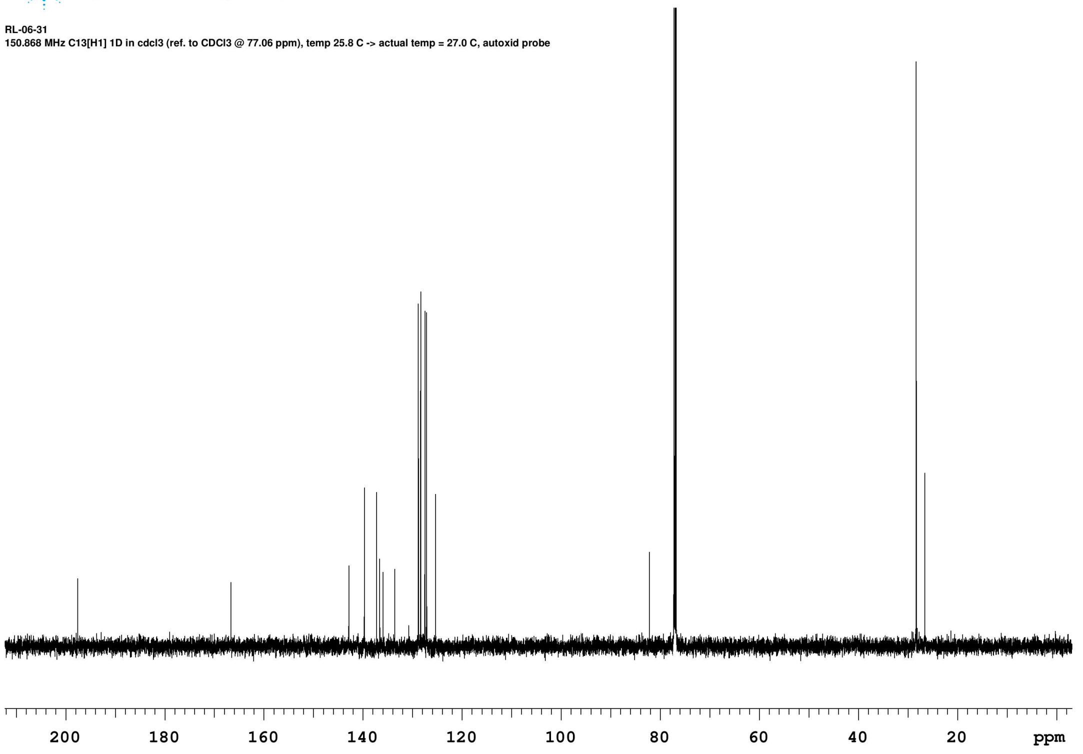

Department of Chemistry, University of Alberta

Agilent Technologies

Recorded on: ibd5, Feb 24 2017 | Sweep Width(Hz): 6000.6 | Acquisiton Time(s): 5 | Relaxation Delay(s): 0.1
Pulse Sequence: s2pul | Digital Res.(Hz/pt): 0.09 | Hz per mm(Hz/mm): 20.74 | Completed Scans 20

PM-10-107
498.118 MHz H1 1D in cdcl3 (ref. to CDCl3 @ 7.26 ppm)
temp 26.9 C -> actual temp = 27.0 C, autoxdb probe

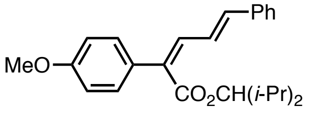

16

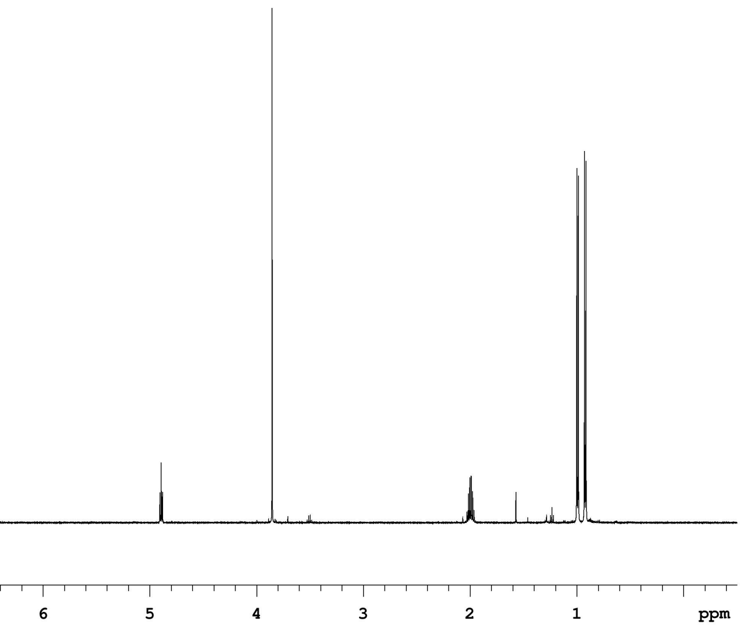

Department of Chemistry, University of Alberta

Agilent Technologies

Recorded on: ibd5, Feb 24 2017 | Sweep Width(Hz): 33826.6 | Acquisiton Time(s): 0.998 | Relaxation Delay(s): 1
Pulse Sequence: s2pul | Digital Res.(Hz/pt): 0.26 | Hz per mm(Hz/mm): 120.18 | Completed Scans 732

PM-10-107
125.266 MHz C13{H1} 1D in cdcl3 (ref. to CDCl3 @ 77.06 ppm)
temp 26.9 C -> actual temp = 27.0 C, autoxdb probe

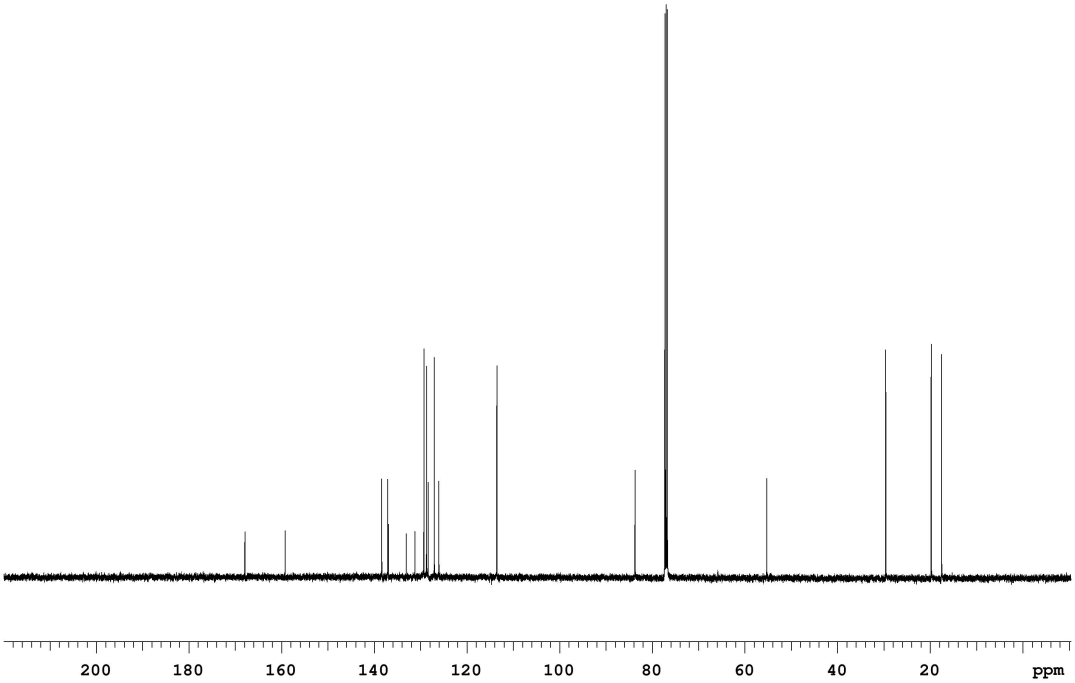

Department of Chemistry, University of Alberta

Agilent Technologies

Recorded on: ibd5, Jan 16 2017 | Sweep Width(Hz): 6000.6 | Acquisiton Time(s): 5 | Relaxation Delay(s): 0.1
Pulse Sequence: s2pul | Digital Res.(Hz/pt): 0.09 | Hz per mm(Hz/mm): 17.29 | Completed Scans 12

RL-06-81-B
498.118 MHz H1 1D in cdcl3 (ref. to CDCl3 @ 7.26 ppm)
temp 26.9 C -> actual temp = 27.0 C, autoxdb probe

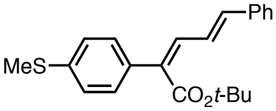

17

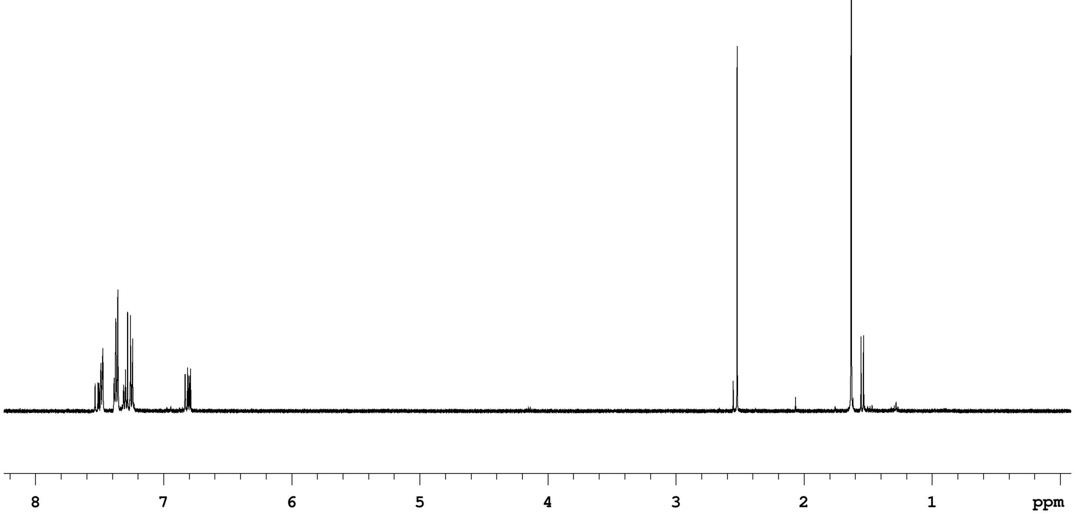

Department of Chemistry, University of Alberta

Agilent Technologies

Recorded on: v700, Jan 16 2017 | Sweep Width(Hz): 48076.9 | Acquisiton Time(s): 1 | Relaxation Delay(s): 1
Pulse Sequence: DEPT_chempack | Digital Res.(Hz/pt): 0.37 | Hz per mm(Hz/mm): 152.74 | Completed Scans 340

Rylan, RL-06-81-B
175.974 MHz C13 DEPTq in cdcl3 (ref. to CDCl3 @ 77.06 ppm)
temp 27.5 C -> actual temp = 27.0 C, coldid probe
DEPTq-135, C & CH2 same, CH & CH3 opposite side of solvent signal

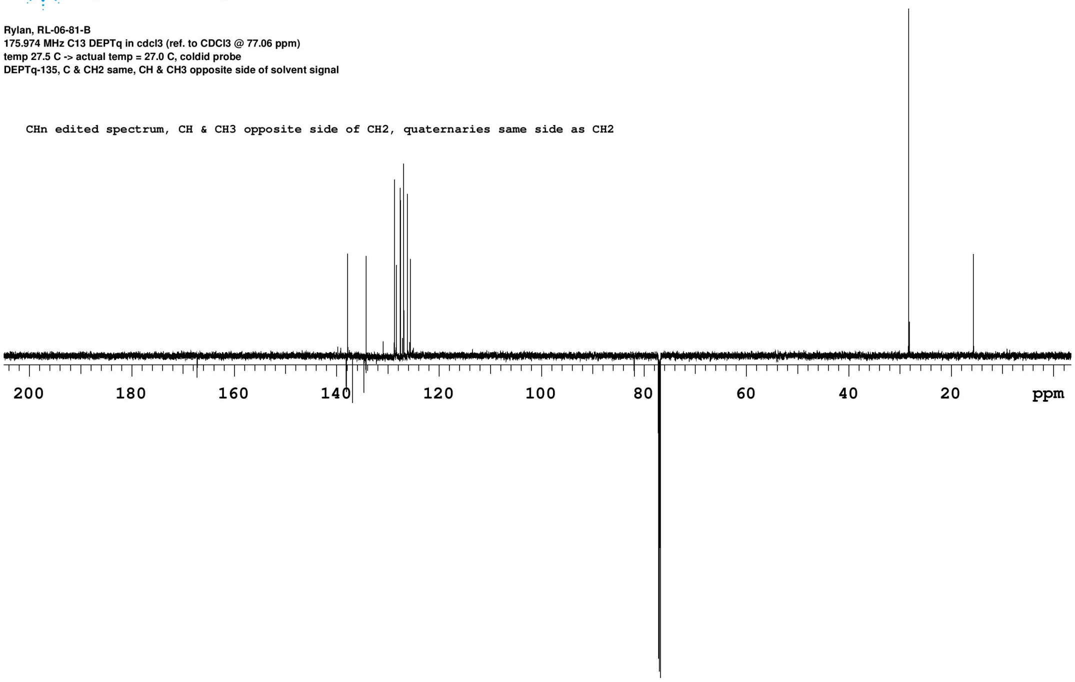

Agilent Technologies

Department of Chemistry, University of Alberta

Recorded on: u500, Aug 30 2016 | Sweep Width(Hz): 6009.62 | Acquisiton Time(s): 5 | Relaxation Delay(s): 0.1

Pulse Sequence: PRESAT | Digital Res.(Hz/pt): 0.09 | Hz per mm(Hz/mm): 17.55 | Completed Scans 16

Rylan, RL-05-199-B

499.797 MHz H1 PRESAT in cdcl3 (ref. to CDCl3 @ 7.26 ppm), temp 27.7 C -> actual temp = 27.0 C, colddual probe

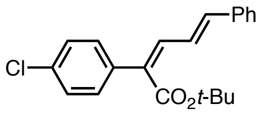

18

Z,E/E,E = 90:10

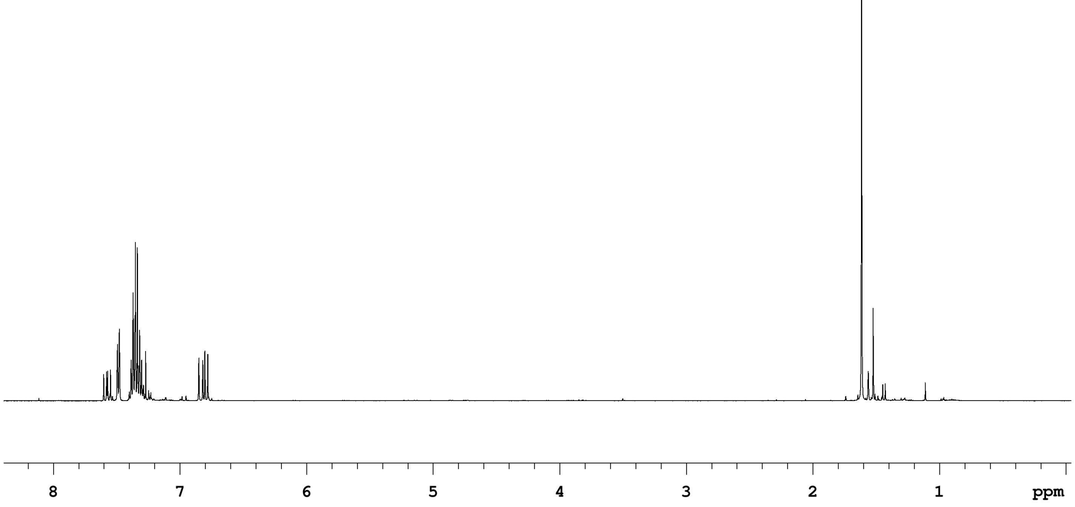

Agilent Technologies

Department of Chemistry, University of Alberta

Recorded on: u500, Aug 30 2016 | Sweep Width(Hz): 33783.8 | Acquisiton Time(s): 1 | Relaxation Delay(s): 1

Pulse Sequence: s2pul | Digital Res.(Hz/pt): 0.26 | Hz per mm(Hz/mm): 102.43 | Completed Scans 512

Rylan, RL-05-199-B

125.688 MHz C13[H1] 1D in cdcl3 (ref. to CDCl3 @ 77.06 ppm), temp 27.7 C -> actual temp = 27.0 C, colddual probe

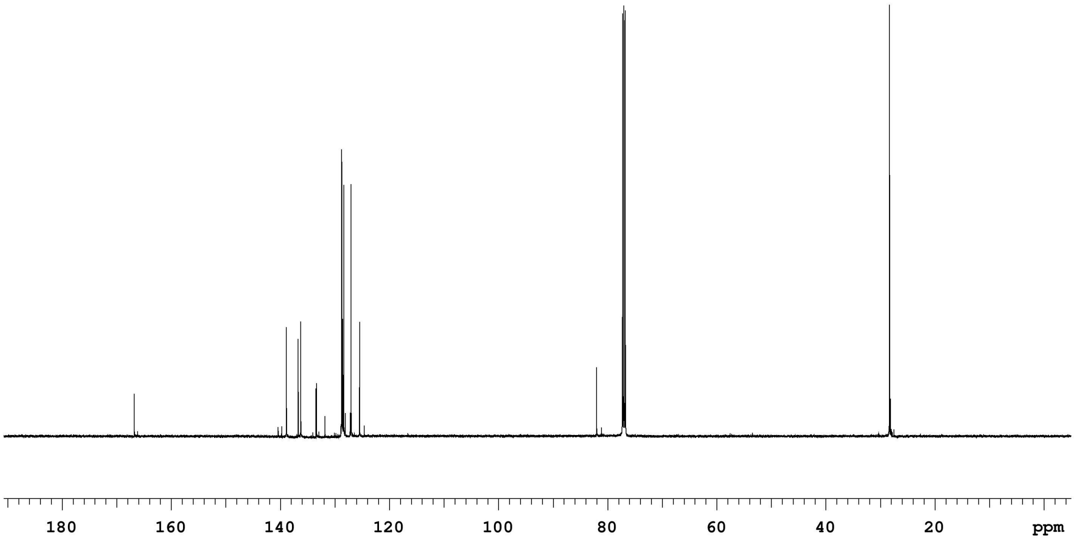

Department of Chemistry, University of Alberta

Agilent Technologies

Recorded on: ibd5, Feb 19 2017
Pulse Sequence: s2pul
Sweep Width(Hz): 6000.6
Digital Res.(Hz/pt): 0.09
Acquisiton Time(s): 5
Hz per mm(Hz/mm): 20.75
Relaxation Delay(s): 0.1
Completed Scans 20

PM-10-089
498.118 MHz H1 1D in cdcl3 (ref. to CDCl3 @ 7.26 ppm)
temp 26.9 C -> actual temp = 27.0 C, autoxdb probe

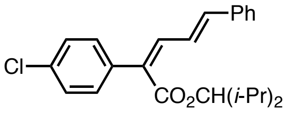

19

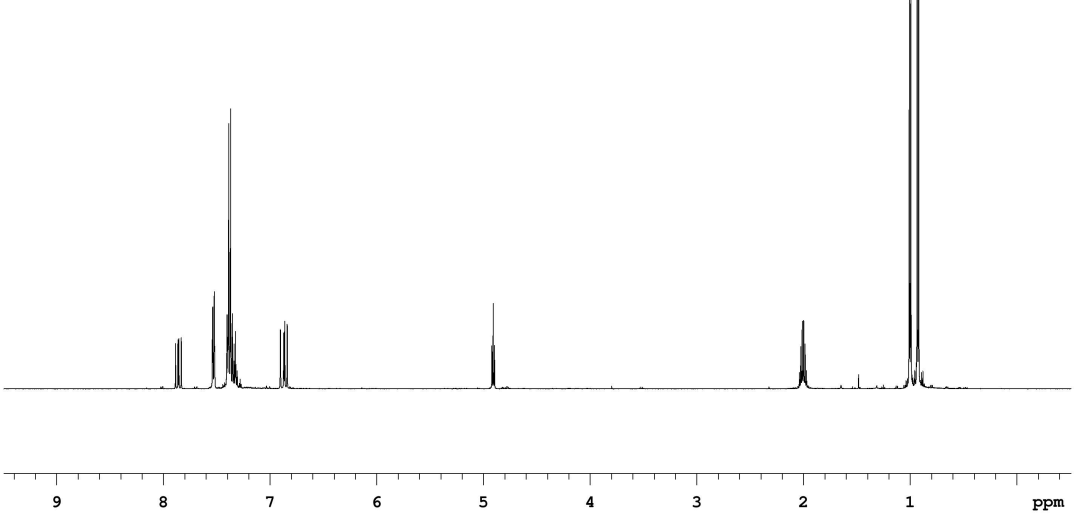

Department of Chemistry, University of Alberta

Agilent Technologies

Recorded on: ibd5, Feb 18 2017
Pulse Sequence: s2pul
Sweep Width(Hz): 33826.6
Digital Res.(Hz/pt): 0.26
Acquisiton Time(s): 0.998
Hz per mm(Hz/mm): 119.95
Relaxation Delay(s): 1
Completed Scans 92

PM-10-089
125.266 MHz C13{H1} 1D in cdcl3 (ref. to CDCl3 @ 77.06 ppm)
temp 26.9 C -> actual temp = 27.0 C, autoxdb probe

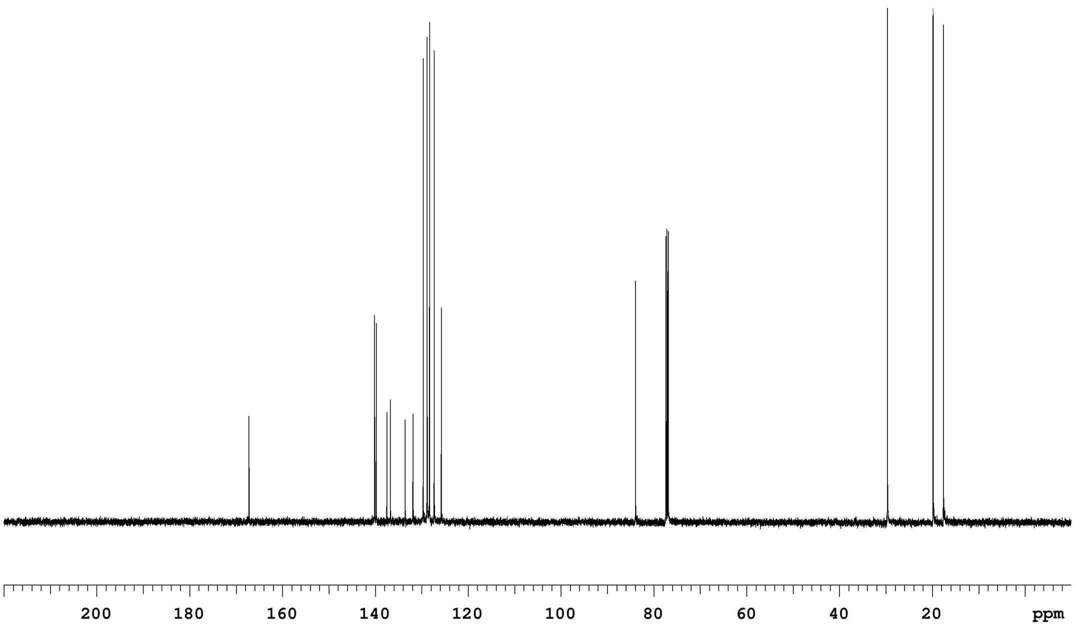

Department of Chemistry, University of Alberta

Agilent Technologies

Recorded on: v700, Feb 3 2017 | Sweep Width(Hz): 8389.26 | Acquisiton Time(s): 5 | Relaxation Delay(s): 0.1

Pulse Sequence: PRESAT | Digital Res.(Hz/pt): 0.13 | Hz per mm(Hz/mm): 24.46 | Completed Scans 16

Rylan, RL-06-85
699.762 MHz H1 1D in cdcl3 (ref. to CDCl3 @ 7.26 ppm)
temp 27.5 C -> actual temp = 27.0 C, coldid probe

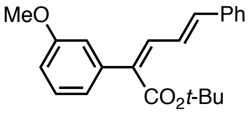

20

Z,E/E,E = 86:14

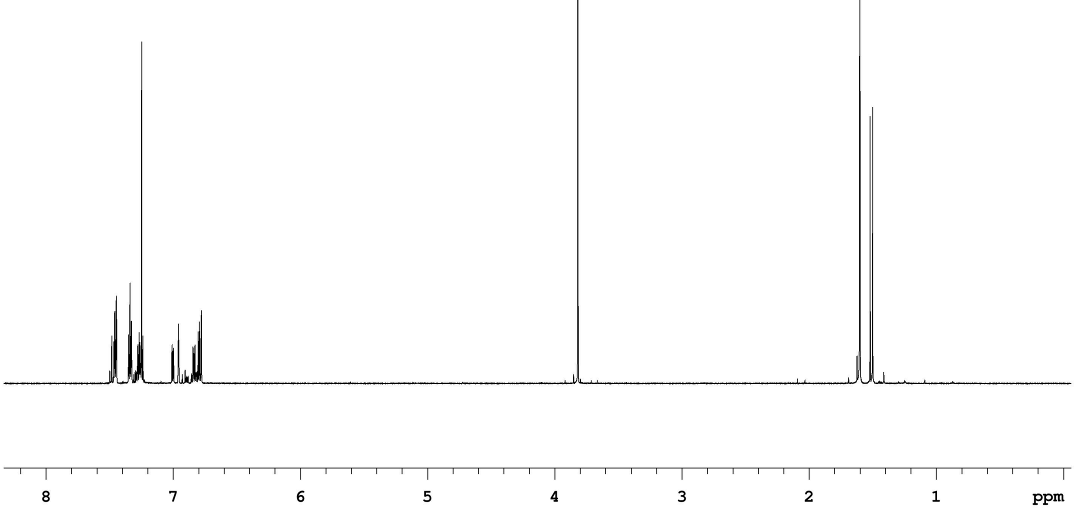

Department of Chemistry, University of Alberta

Agilent Technologies

Recorded on: v700, Feb 3 2017 | Sweep Width(Hz): 48076.9 | Acquisiton Time(s): 1 | Relaxation Delay(s): 1

Pulse Sequence: s2pul | Digital Res.(Hz/pt): 0.37 | Hz per mm(Hz/mm): 155.07 | Completed Scans 512

Rylan, RL-06-85
175.975 MHz C13{H1} 1D in cdcl3 (ref. to CDCl3 @ 77.06 ppm)
temp 27.5 C -> actual temp = 27.0 C, coldid probe

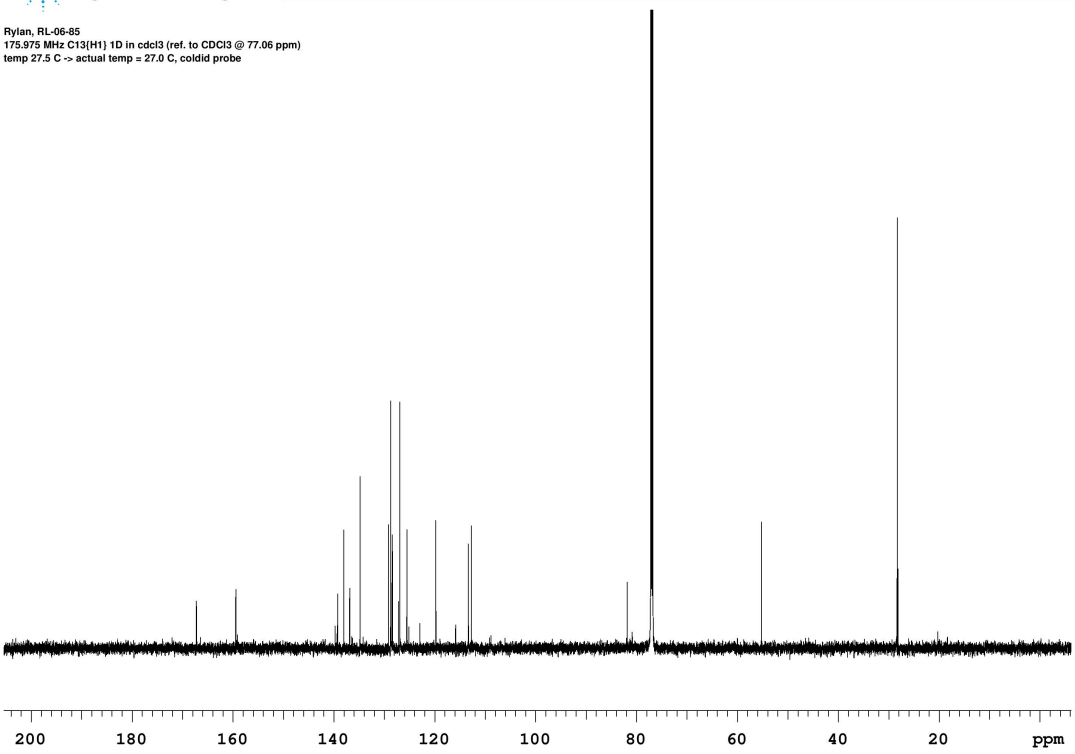

Agilent Technologies

Department of Chemistry, University of Alberta

Recorded on: ibd5, Feb 19 2017	Sweep Width(Hz): 6000.6	Acquisiton Time(s): 5	Relaxation Delay(s): 0.1
Pulse Sequence: s2pul	Digital Res.(Hz/pt): 0.09	Hz per mm(Hz/mm): 20.77	Completed Scans 24

PM-10-085-B
498.118 MHz H1 1D in cdcl3 (ref. to CDCl3 @ 7.26 ppm)
temp 26.9 C -> actual temp = 27.0 C, autoxdb probe

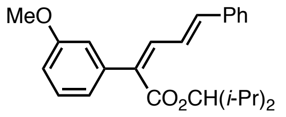

21

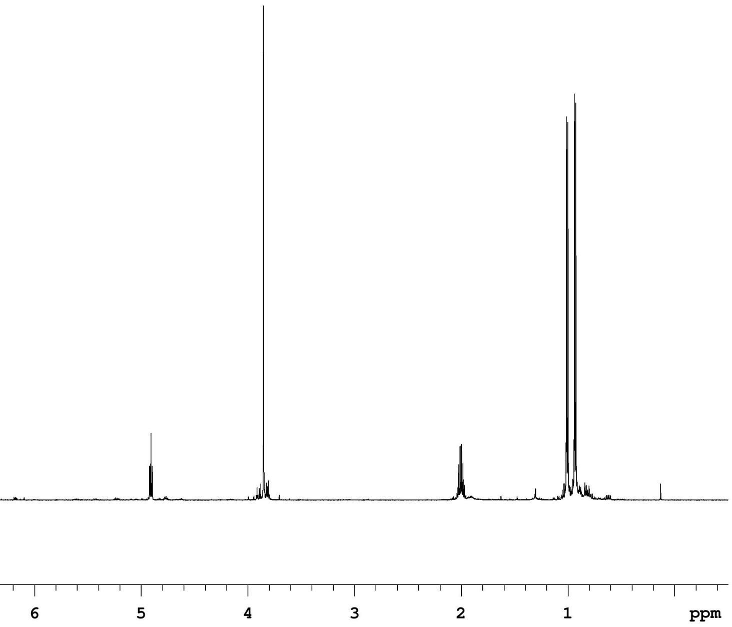

Agilent Technologies

Department of Chemistry, University of Alberta

Recorded on: ibd5, Feb 19 2017	Sweep Width(Hz): 33826.6	Acquisiton Time(s): 0.998	Relaxation Delay(s): 1
Pulse Sequence: s2pul	Digital Res.(Hz/pt): 0.26	Hz per mm(Hz/mm): 120.11	Completed Scans 160

PM-10-085-B
125.266 MHz C13{H1} 1D in cdcl3 (ref. to CDCl3 @ 77.06 ppm)
temp 26.9 C -> actual temp = 27.0 C, autoxdb probe

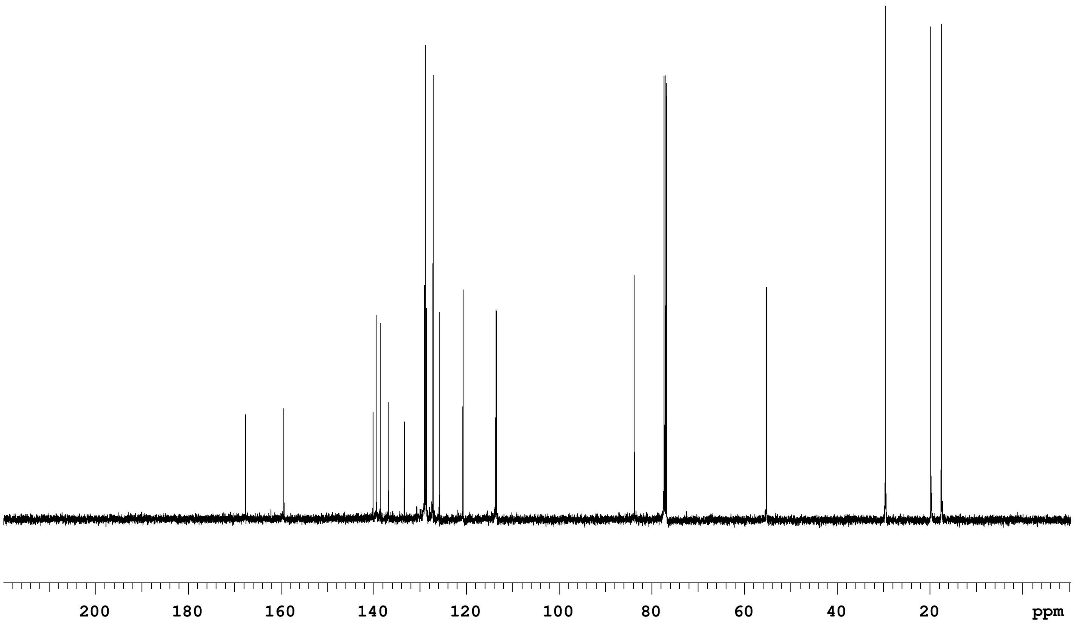

Department of Chemistry, University of Alberta

Agilent Technologies

Recorded on: v700, Sep 26 2016 | Sweep Width(Hz): 8389.26 | Acquisiton Time(s): 5 | Relaxation Delay(s): 0.1
Pulse Sequence: PRESAT | Digital Res.(Hz/pt): 0.13 | Hz per mm(Hz/mm): 25.07 | Completed Scans 16

Rylan, RL-06-35-C
699.762 MHz H1 PRESAT in cdcl3 (ref. to CDCl3 @ 7.26 ppm), temp 27.5 C -> actual temp = 27.0 C, coldid probe

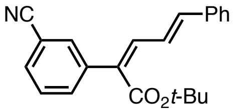

22

Z,E/E,E = 63:37

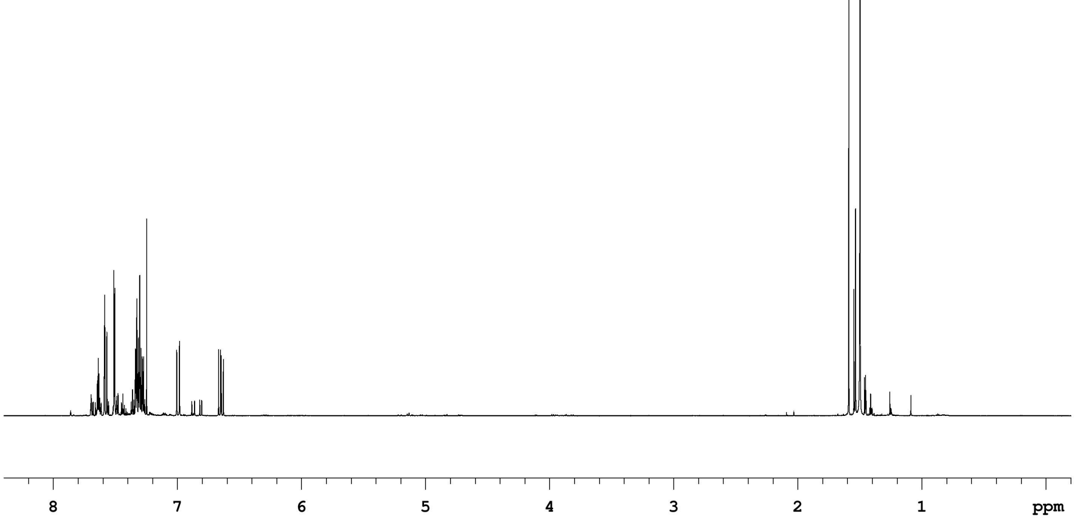

Department of Chemistry, University of Alberta

Agilent Technologies

Recorded on: v700, Sep 26 2016 | Sweep Width(Hz): 48076.9 | Acquisiton Time(s): 1 | Relaxation Delay(s): 1
Pulse Sequence: s2pul | Digital Res.(Hz/pt): 0.37 | Hz per mm(Hz/mm): 155.3 | Completed Scans 512

Rylan, RL-06-35-C
175.975 MHz C13[H1] 1D in cdcl3 (ref. to CDCl3 @ 77.06 ppm), temp 27.5 C -> actual temp = 27.0 C, coldid probe

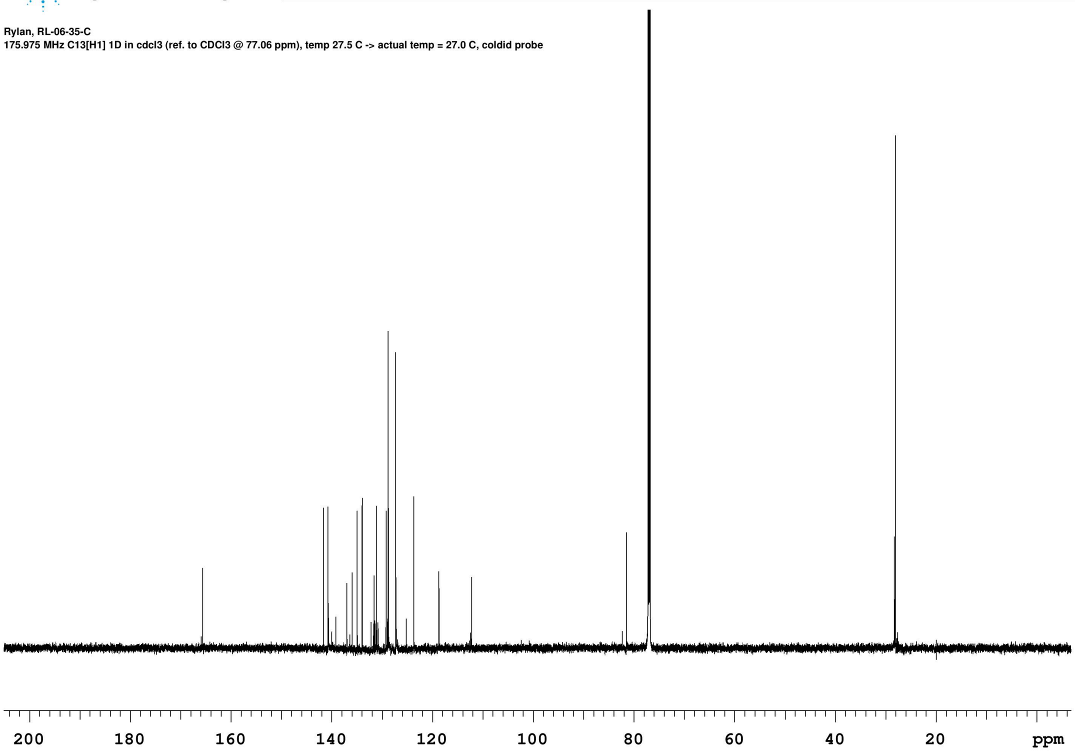

Department of Chemistry, University of Alberta

Agilent Technologies

Recorded on: u500, Aug 30 2016 | Sweep Width(Hz): 6009.62 | Acquisiton Time(s): 5 | Relaxation Delay(s): 0.1
Pulse Sequence: PRESAT | Digital Res.(Hz/pt): 0.09 | Hz per mm(Hz/mm): 17.52 | Completed Scans 16

Bryce, BT-08-021-F29-33
499.797 MHz H1 PRESAT in cdcl3 (ref. to CDCl3 @ 7.26 ppm), temp 27.7 C -> actual temp = 27.0 C, colddual probe

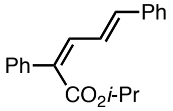

24

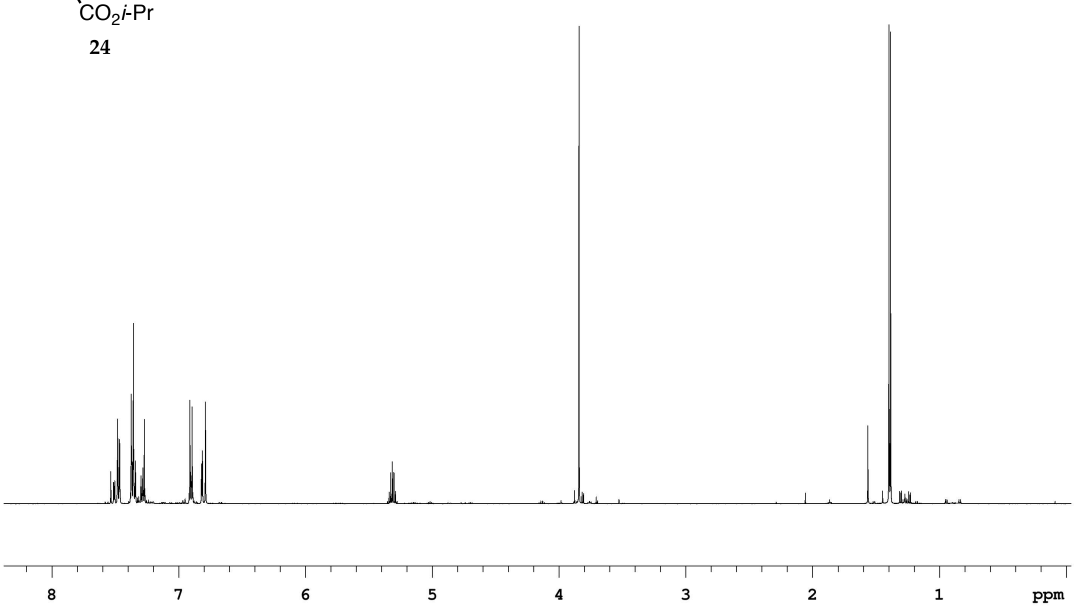

Department of Chemistry, University of Alberta

Agilent Technologies

Recorded on: u500, Aug 30 2016 | Sweep Width(Hz): 33783.8 | Acquisiton Time(s): 1 | Relaxation Delay(s): 1
Pulse Sequence: s2pul | Digital Res.(Hz/pt): 0.26 | Hz per mm(Hz/mm): 106.92 | Completed Scans 128

Bryce, BT-08-021-F29-33
125.688 MHz C13[H1] 1D in cdcl3 (ref. to CDCl3 @ 77.06 ppm), temp 27.7 C -> actual temp = 27.0 C, colddual probe

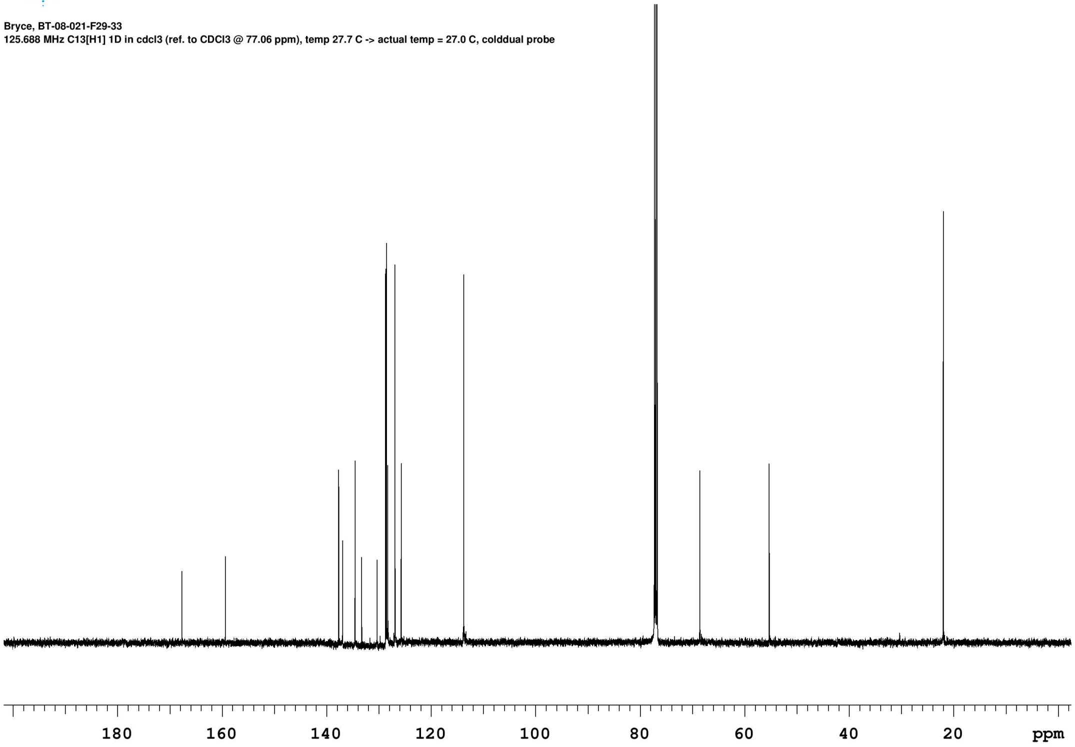

Department of Chemistry, University of Alberta

Agilent Technologies

Recorded on: ibd5, Aug 29 2016 | Sweep Width(Hz): 6000.6 | Acquisiton Time(s): 5 | Relaxation Delay(s): 0.1
Pulse Sequence: s2pul | Digital Res.(Hz/pt): 0.09 | Hz per mm(Hz/mm): 17.71 | Completed Scans 28

RL-05-197
498.118 MHz H1 1D in cdcl3 (ref. to CDCl3 @ 7.26 ppm), temp 26.4 C -> actual temp = 27.0 C, autoxdb probe

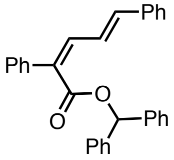

25

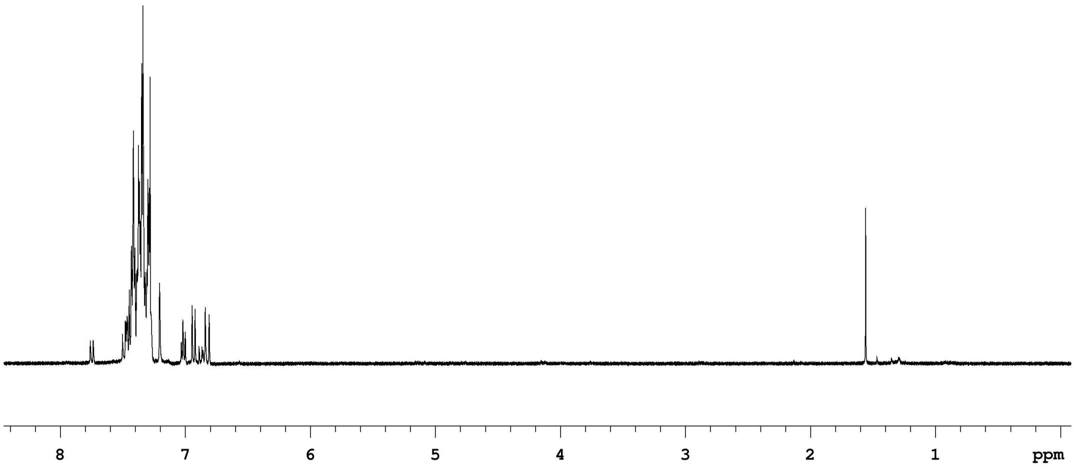

Department of Chemistry, University of Alberta

Agilent Technologies

Recorded on: u500, Aug 29 2016 | Sweep Width(Hz): 33783.8 | Acquisiton Time(s): 1 | Relaxation Delay(s): 1
Pulse Sequence: s2pul | Digital Res.(Hz/pt): 0.26 | Hz per mm(Hz/mm): 110.44 | Completed Scans 512

Rylan, RL-05-197
125.688 MHz C13[H1] 1D in cdcl3 (ref. to CDCl3 @ 77.06 ppm), temp 27.7 C -> actual temp = 27.0 C, colddual probe

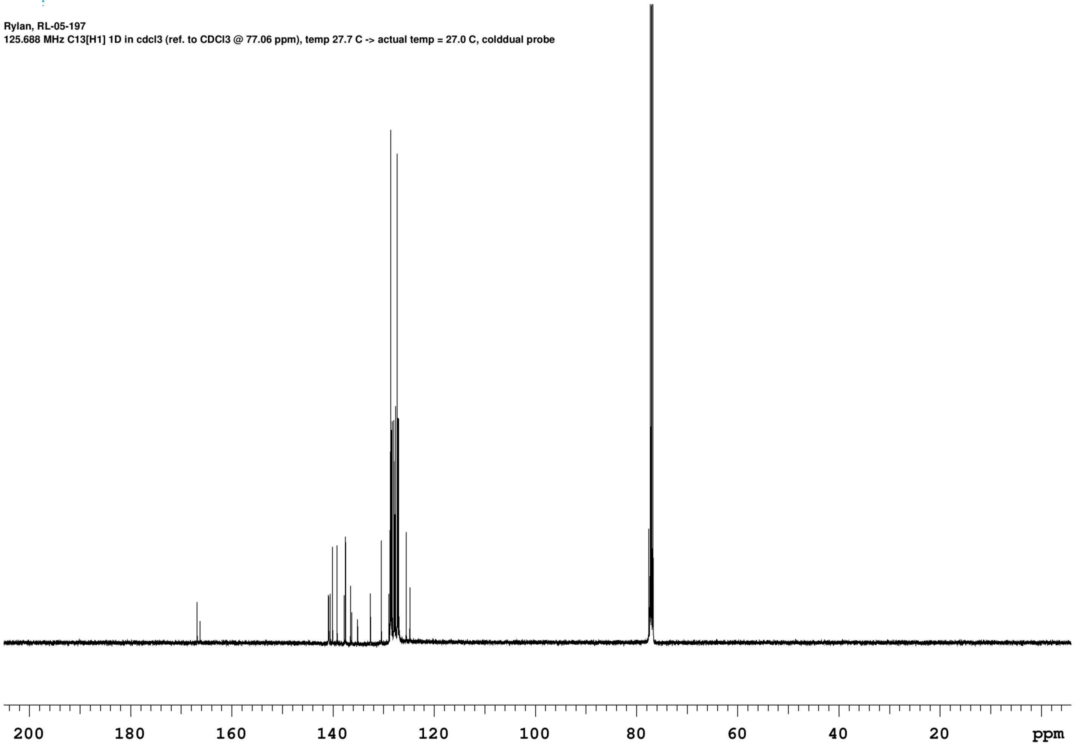

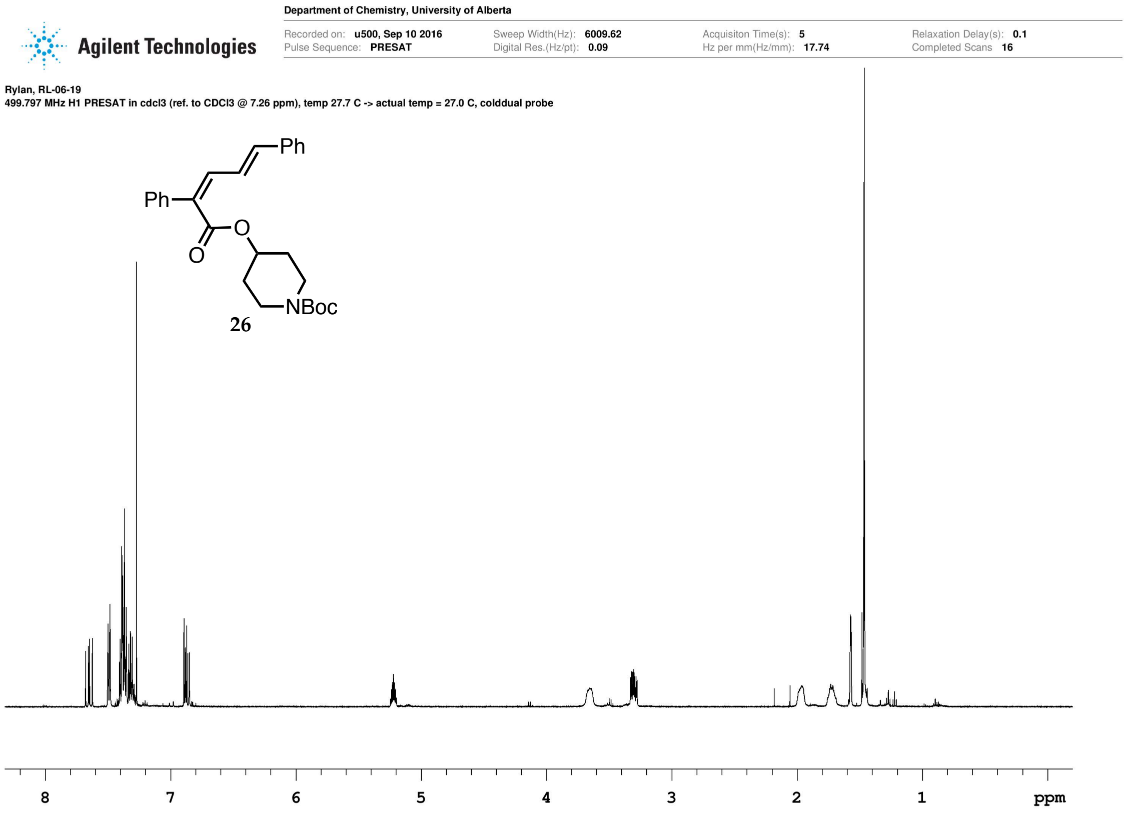
Department of Chemistry, University of Alberta
Agilent Technologies
Recorded on: u500, Sep 10 2016
Pulse Sequence: PRESAT
Sweep Width(Hz): 6009.62
Digital Res.(Hz/pt): 0.09
Acquisiton Time(s): 5
Hz per mm(Hz/mm): 17.74
Relaxation Delay(s): 0.1
Completed Scans 16
Rylan, RL-06-19
499.797 MHz H1 PRESAT in cdcl3 (ref. to CDCl3 @ 7.26 ppm), temp 27.7 C -> actual temp = 27.0 C, colddual probe
Ph
Ph
O
O
NBoc
26
8
7
6
5
4
3
2
1
ppm

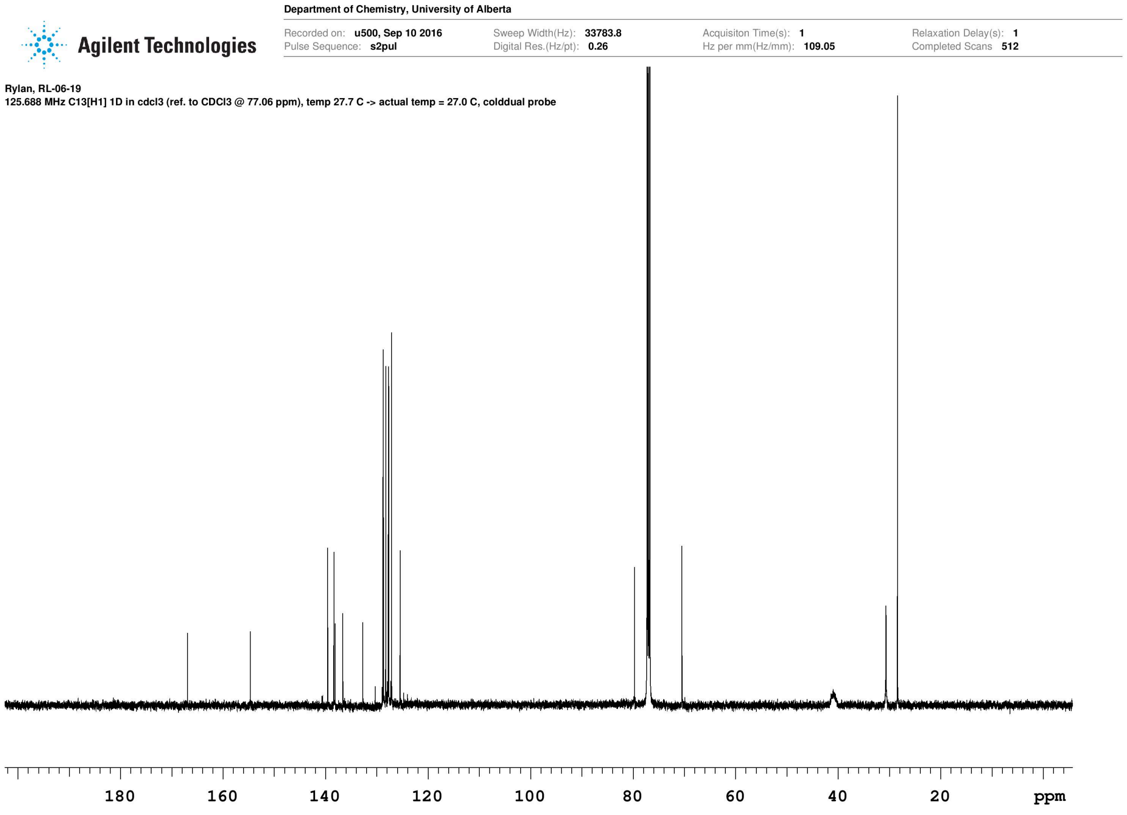
Department of Chemistry, University of Alberta
Agilent Technologies
Recorded on: u500, Sep 10 2016
Pulse Sequence: s2pul
Sweep Width(Hz): 33783.8
Digital Res.(Hz/pt): 0.26
Acquisiton Time(s): 1
Hz per mm(Hz/mm): 109.05
Relaxation Delay(s): 1
Completed Scans 512
Rylan, RL-06-19
125.688 MHz C13[H1] 1D in cdcl3 (ref. to CDCl3 @ 77.06 ppm), temp 27.7 C -> actual temp = 27.0 C, colddual probe
180
160
140
120
100
80
60
40
20
ppm

Agilent Technologies

Department of Chemistry, University of Alberta

Recorded on: **v700, Feb 1 2017**	Sweep Width(Hz): **8389.26**	Acquisiton Time(s): **5**	Relaxation Delay(s): **0.1**
Pulse Sequence: **PRESAT**	Digital Res.(Hz/pt): **0.13**	Hz per mm(Hz/mm): **24.64**	Completed Scans **16**

Rylan, RL-06-83
699.762 MHz H1 1D in cdcl3 (ref. to CDCl3 @ 7.26 ppm)
temp 27.5 C -> actual temp = 27.0 C, coldid probe

** di-octyl phthalate (~4%)*

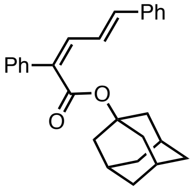

27

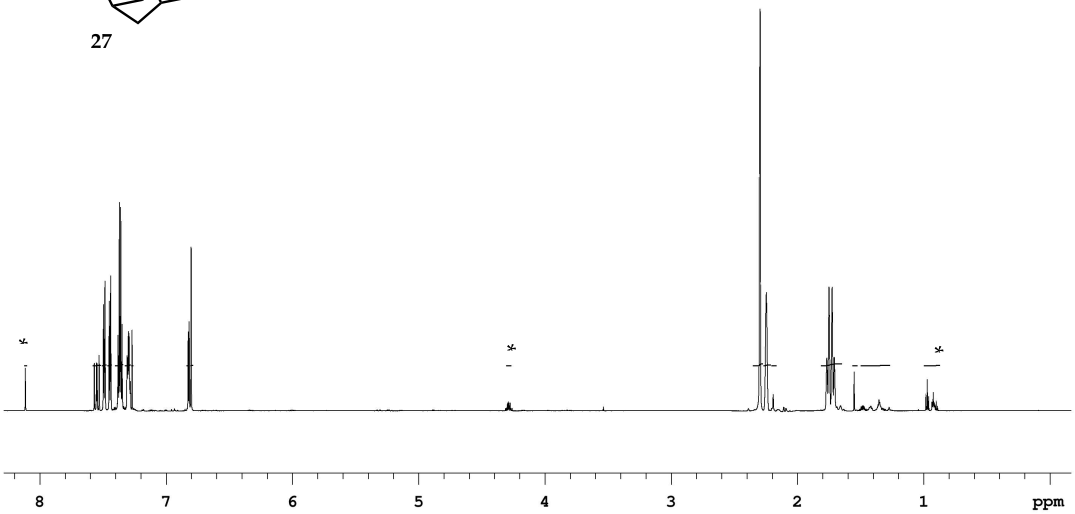

Agilent Technologies

Department of Chemistry, University of Alberta

Recorded on: **v700, Feb 1 2017**	Sweep Width(Hz): **48076.9**	Acquisiton Time(s): **1**	Relaxation Delay(s): **1**
Pulse Sequence: **s2pul**	Digital Res.(Hz/pt): **0.37**	Hz per mm(Hz/mm): **152.39**	Completed Scans **1000**

Rylan, RL-06-83
175.975 MHz C13{H1} 1D in cdcl3 (ref. to CDCl3 @ 77.06 ppm)
temp 27.5 C -> actual temp = 27.0 C, coldid probe

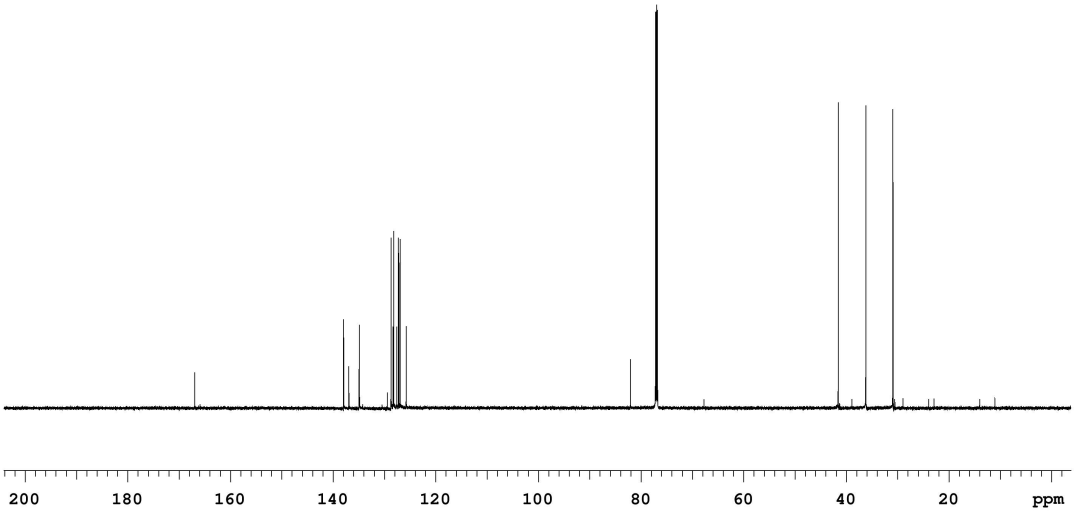

Department of Chemistry, University of Alberta

Agilent Technologies

Recorded on: u500, Aug 5 2016 | Sweep Width(Hz): 6009.62 | Acquisiton Time(s): 5 | Relaxation Delay(s): 0.1
Pulse Sequence: PRESAT | Digital Res.(Hz/pt): 0.09 | Hz per mm(Hz/mm): 17.75 | Completed Scans 16

Bryce, BT-07-203-A
499.797 MHz H1 PRESAT in cdcl3 (ref. to CDCl3 @ 7.26 ppm), temp 27.7 C -> actual temp = 27.0 C, colddual probe

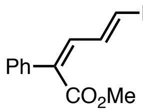

S1

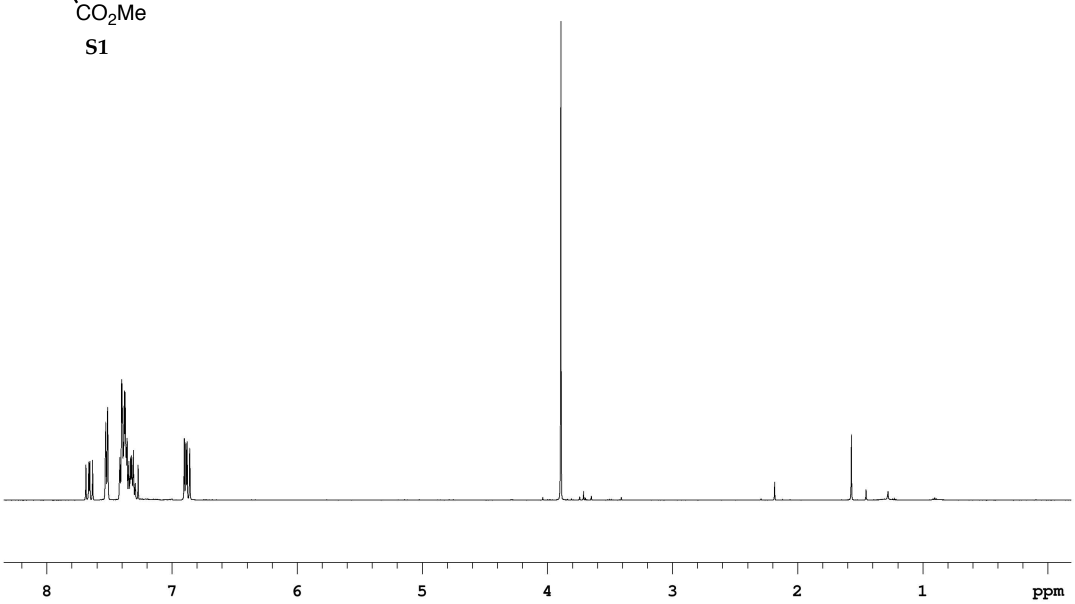

Department of Chemistry, University of Alberta

Agilent Technologies

Recorded on: u500, Aug 5 2016 | Sweep Width(Hz): 33783.8 | Acquisiton Time(s): 1 | Relaxation Delay(s): 1
Pulse Sequence: s2pul | Digital Res.(Hz/pt): 0.26 | Hz per mm(Hz/mm): 107.82 | Completed Scans 128

Bryce, BT-07-203-A
125.688 MHz C13[H1] 1D in cdcl3 (ref. to CDCl3 @ 77.06 ppm), temp 27.7 C -> actual temp = 27.0 C, colddual probe

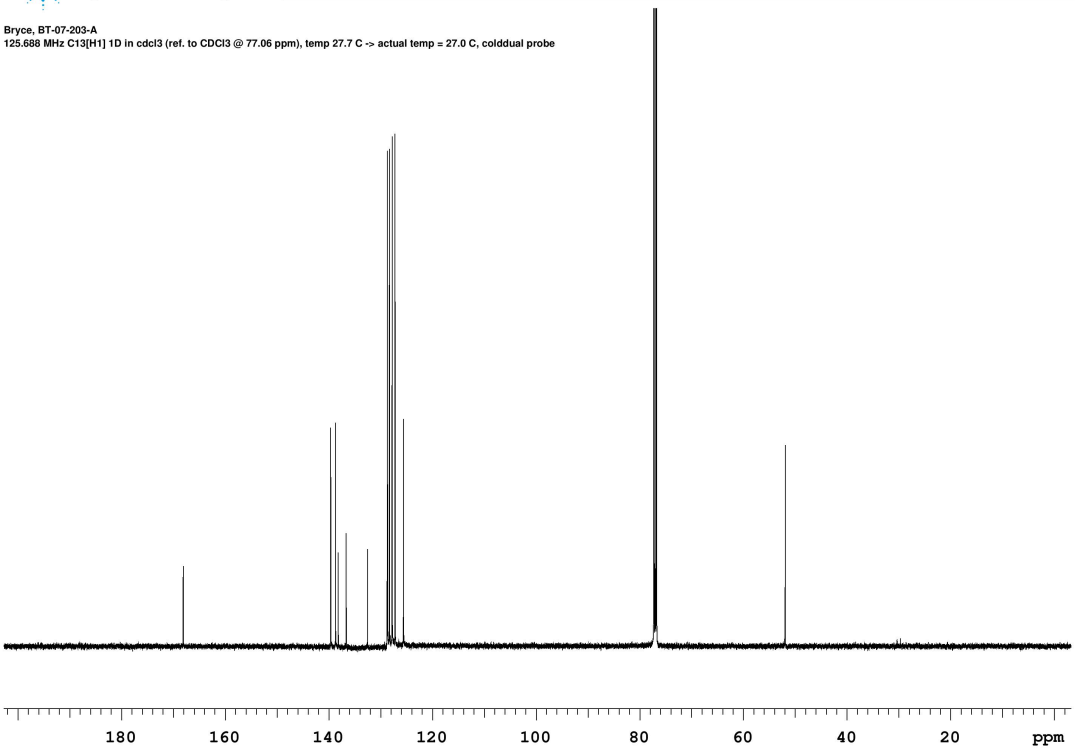

Supplement: Supplementary file 1 [file SC-009-C7SC04283C-s001.pdf]
